# Esterase-responsive albumin-binding PROTAC-mediated BRD4 degradation for cancer immunotherapy

**DOI:** 10.7150/thno.130510

**Published:** 2026-04-23

**Authors:** Hoyeon Lee, Sojin Jeong, Juwon Park, Hye-jeong Son, Seho Kweon, Steve Seung-Young Lee, Nayeon Shim, Yoojeong Oh, Hyein Kang, Chaerin Lee, Jihyeon Lee, Jinseong Kim, Hanhee Cho, Kwangmeyung Kim

**Affiliations:** 1 College of Pharmacy, Graduate School of Pharmaceutical Sciences, Ewha Womans University, Seoul, 03760, Republic of Korea.; 2 Graduate Program in Innovative Biomaterials Convergence, Ewha Womans University, Seoul 03760, Korea.; 3 Medicinal Materials Research Center, Biomedical Research Division, Medicinal Materials Research Center, Korea Institute of Science and Technology (KIST), Seoul 02792, Republic of Korea.; 4 College of Pharmacy, Chonnam National University, Gwangju 61186, Republic of Korea.; 5 Department of Pharmaceutical Sciences, University of Illinois Chicago, Chicago, IL, USA.; 6 Noxpharm Co., LTD., Seoul 03759, Republic of Korea.

**Keywords:** PROTACs, BRD4 degradation, Esterase-cleavable maleimide linker, Albumin-binding, Cancer immunotherapy

## Abstract

**Rationale:**

Proteolysis-targeting chimeras (PROTACs) represent a powerful therapeutic modality for selective protein degradation but often suffer from poor pharmacokinetics and limited tumor-targeting. To overcome these constraints, we developed albumin-binding BRD4-degrading PROTACs (Alb-TACs) with esterase-cleavable maleimide linkers that hitchhike endogenous albumin and enable esterase-responsive BRD4 degradation in tumors.

**Methods:**

Alb-TACs were synthesized by conjugating two esterase-cleavable maleimide linkers, bicyclononyne-polyethylene glycol-maleimide (BCN-PEG_2_-Mal) or N-(2-aminoethyl)maleimide (AE-Mal), to BRD4-degrading PROTAC (ARV-771), resulting in Alb-TAC#1 and Alb-TAC#2, with distinct albumin- and esterase-binding properties. To select effective Alb-TAC, the binding ability to albumin and esterase-specific cleavage of Alb-TACs were carefully assayed using MALDI-TOF, PAGE, and time-course HPLC. Furthermore, the tumor-targeting efficacy of Alb-TACs was assessed by fluorescence imaging in CT26 tumor-bearing BALB/c mice. Next, we investigated the BRD4 degrading efficiency of Alb-TAC in a cell culture system and in CT26 tumor-bearing mice. Finally, the immunogenic cell death (ICD) and reprogrammed immune cells of Alb-TAC-treated tumors were carefully characterized.

**Results:**

Alb-TAC#2 containing the AE-Mal linker exhibited rapid albumin binding, accelerated esterase-responsive activation, and enhanced tumor accumulation compared to ARV-771 and Alb-TAC#1 due to its flexible chemical structure. In the CT26 cell culture system, Alb-TAC#2 efficiently degraded BRD4, resulting in BRD4-deficient cell death. Furthermore, in CT26 tumor-bearing mice, Alb-TAC#2 achieved extensive apoptosis through robust BRD4 degradation, leading to marked downregulation of c-Myc, Bcl-2, and PD-L1. Moreover, Alb-TAC#2 induced hallmarks of ICD (elevated surface CRT, extracellular ATP, and HMGB1) and reprogrammed the tumor microenvironment by enhancing CD8⁺ T cell infiltration, promoting dendritic cell maturation, and reducing regulatory T cell function.

**Conclusions:**

This esterase-responsive albumin-binding PROTAC design could overcome pharmacokinetic barriers of conventional BRD4-targeting PROTACs by enhancing tumor-specific delivery and esterase-responsive BRD4 degradation in solid tumors. In summary, esterase-responsive albumin-binding PROTAC is proven as a promising strategy that effectively modulates the pharmacokinetics and therapeutic performance of PROTACs for cancer immunotherapy.

## Introduction

Targeted protein degradation (TPD) has emerged as a promising therapeutic strategy due to its ability to address proteins previously considered undruggable by conventional small molecules 1, 2. Among various TPD approaches, proteolysis-targeting chimeras (PROTACs) are heterobifunctional molecules that induce selective degradation of target proteins by simultaneously recruiting the protein of interest and an E3 ubiquitin ligase, resulting in ubiquitination and subsequent proteasomal degradation 3, 4. A well-established target of this approach is bromodomain-containing protein 4 (BRD4), a member of the bromodomain and extra-terminal (BET) family that is considered a major driver of cancer cell proliferation and has emerged as a promising therapeutic target in oncology. Importantly, BRD4 acts as a critical epigenetic and transcriptional regulator, is frequently overexpressed in various malignancies, and induces tumor progression by regulating the transcription of oncogenes such as MYC (MYC proto-oncogene, bHLH transcription factor) and BCL2 (B-cell lymphoma 2) 5-10. Consequently, BRD4-targeting PROTACs such as ARV-771 have demonstrated potent antitumor efficacy by selectively degrading BRD4 and suppressing downstream oncogenic signaling pathways 11-13. Despite these promising therapeutic outcomes, conventional PROTACs are often limited to treatment in solid tumors due to suboptimal pharmacokinetic behavior, rapid clearance, and poor tumor targeting 14-16. Therefore, achieving prolonged circulation and tumor-specific delivery remains a critical challenge for the clinical translation of PROTACs for cancer therapy.

Recently, various nanomaterial-based delivery systems have been explored to overcome the intrinsic pharmacokinetic limitations of BRD4-targeting PROTACs. For example, lipid-based nanoparticles, including liposomes and lipid bilayer vesicles, have been used to encapsulate hydrophobic BET degraders such as ARV-825, enabling enhanced permeability and retention (EPR) effect-driven tumor accumulation through nanoscale formulation engineering 17, 18. Various polymeric nanoparticles also improved the tumor targeting efficiency of PROTACs encapsulated in their hydrophobic inner cores through passive or ligand-mediated active targeting 19-21. In addition, polymeric micelle systems containing stimuli-responsive linkages, such as pH- and redox-cleavable bonds, have also been developed to achieve activatable or microenvironment-triggered release of PROTACs in targeted tumor tissues 20, 22. While these strategies could enhance the tumor targeting efficiency of PROTACs, most rely on exogenous nanoparticle-based delivery systems, which may introduce other challenges such as complex manufacturing, potential toxicity, premature drug release (dumping), and immunogenicity 14, 23, 24. Consequently, there remains a pressing need for a biocompatible, long-circulating, and endogenously compatible drug delivery system capable of achieving efficient tumor localization and on-site activation of BRD4-degrading PROTACs for cancer therapy.

Among various delivery systems, albumin, a highly abundant serum protein, has emerged as an ideal endogenous drug carrier owing to its long half-life, biocompatibility, and intrinsic tumor tropism 25-30. In particular, albumin can be accumulated within solid tumors through its EPR effect and gp60/SPARC-mediated transcytosis and is actively taken up by proliferating cancer cells to meet metabolic demands 31-36. The clinical success of Abraxane, an albumin-bound formulation of paclitaxel, further demonstrated the safety and therapeutic potential of albumin-based delivery systems to improve pharmacokinetic stability and tumor targeting efficiency 37, 38. These features comprehensively highlight the potential of albumin to improve bioavailability and tumor targeting as an endogenous drug delivery system of PROTACs. Building on this rationale, we previously developed albumin-binding BRD4-degrading PROTACs (Alb-TAC) with an esterase-cleavable maleimide linker that could enable *in situ* albumin conjugation and esterase-responsive release of BRD4-degrading PROTACs at targeted tumor tissues 39. In this strategy, maleimide-functionalized Alb-TAC selectively formed covalent bonds with the free thiol group, primarily Cys34, of circulating albumin under physiological conditions 40, 41. This site-specific conjugation enables stable and efficient binding of Alb-TAC to endogenous albumin *in vivo*, thereby improving the pharmacokinetic profile and tumor-targeting efficiency. Furthermore, Alb-TAC permitted controlled release of active PROTACs in response to elevated esterase activity within the tumor microenvironment (TME) 37, 42-45. Compared with light- or redox/GSH-responsive strategies, this enzyme-triggered design enables device-independent activation and provides a kinetically regulated, sustained intracellular release of the active PROTAC following tumor-cell uptake 46-51. However, the cleavage kinetics of the initial esterase-cleavable linker were not fully understood, raising concerns about whether albumin binding efficacy and intracellular esterase-specific cleavage are sufficiently rapid or efficient. To address this limitation, alternative esterase-cleavable linkers should be systemically compared, leading to the development of potential Alb-TAC, a next-generation BRD4-targeting PROTAC that maintains albumin-binding capacity while exhibiting accelerated cleavage kinetics and enhanced pharmacodynamic efficacy.

**Scheme 1:**
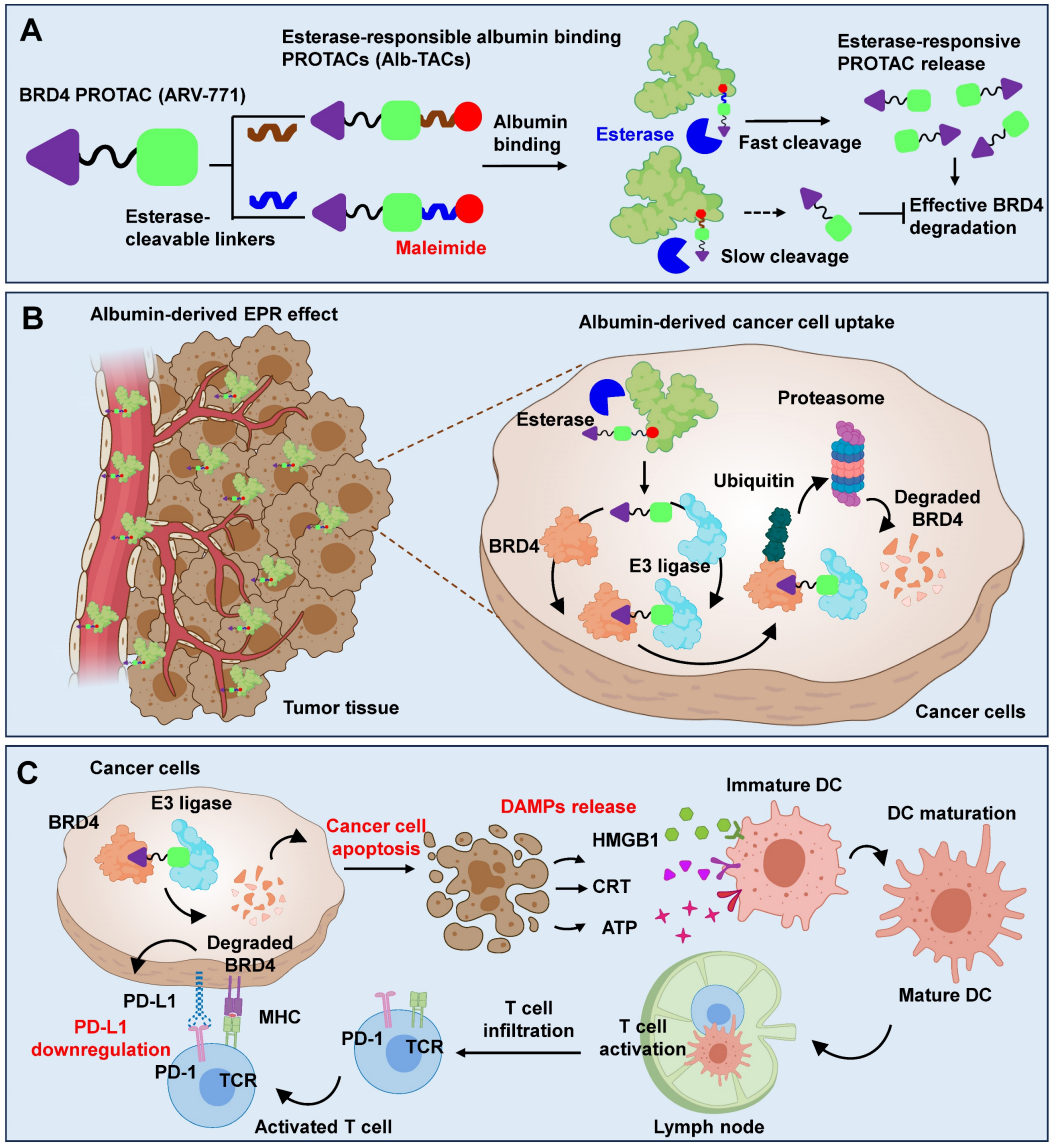
** Schematic illustration for the MOA of Alb-TACs.** (A) *In situ* albumin-binding, esterase-cleavable BRD4-degrading PROTACs (Alb-TACs) are developed by conjugating a maleimide-terminated, esterase-cleavable linker to the ARV-771 molecule. Following intravenous administration, Alb-TACs spontaneously bind to plasma albumin *via* a site-specific thiol-maleimide reaction. Once internalized into cancer cells, the linker is cleaved by intracellular esterase, releasing free ARV-771 to initiate BRD4 degradation. (B) Through *in situ* albumin binding, Alb-TACs exhibit prolonged systemic circulation and preferential tumor accumulation *via* the EPR effect. Albumin-bound Alb-TAC is actively internalized by cancer cells, where esterase-mediated cleavage regenerates free ARV-771. The released ARV-771 simultaneously binds BRD4 and the VHL E3 ligase, inducing BRD4 polyubiquitination and subsequent proteasomal degradation. ARV-771 is then recycled to engage additional BRD4 molecules, enabling catalytic degradation. (C) BRD4 degradation by Alb-TACs suppresses downstream oncogenic targets, induces apoptosis, and promotes the release of damage-associated molecular patterns (DAMPs) such as calreticulin (CRT), ATP, and high-mobility group box 1 (HMGB1). These DAMPs act as immunogenic signals that stimulate dendritic cell (DC) maturation and antigen presentation, leading to enhanced activation and infiltration of cytotoxic CD8+ T cells. In parallel, BRD4 degradation downregulates PD-L1 expression, alleviating T-cell exhaustion and restoring antitumor immunity.

Herein, we rationally synthesized two albumin-binding BRD4-targeting PROTAC candidates (Alb-TACs) with two esterase-cleavable maleimide linkers that have different abilities to bind esterase and albumin, respectively. Specifically, esterase-cleavable maleimide linkers, bicyclononyne-polyethylene glycol-maleimide (BCN-PEG_2_-Mal) and N-(2-aminoethyl)maleimide (AE-Mal), are employed to BRD4-degrading PROTAC, ARV-771, in the resulting ARV-771-BCN-PEG_2_-Mal (Alb-TAC#1) and ARV-771-AE-Mal (Alb-TAC#2), respectively (**Scheme 1A**). These two Alb-TAC candidates are systematically compared to find the best linker scaffold. The site-specific conjugation of esterase-cleavable maleimide linkers enables stable and efficient binding to endogenous albumin *in vivo*, thereby improving the pharmacokinetic profile and tumor-targeting efficiency of intravenously administered Alb-TACs. Furthermore, the esterase-cleavable maleimide linkers in Alb-TACs are designed to respond to elevated esterase activity within the TME, triggering localized release of BRD4-degrading PROTACs 37, 42-45. Following *in situ* albumin conjugation, the resulting Alb-TACs exhibit prolonged systemic circulation and enhanced tumor accumulation *via* the EPR effect (**Scheme 1B**). Once internalized by cancer cells, esterase-mediated cleavage of Alb-TACs facilitates release of BRD4-degrading PROTAC, which induces E3-mediated ubiquitination and proteasomal degradation of BRD4. Beyond its role in driving oncogenic transcription, BRD4 has recently been implicated in regulating the immune landscape of TME 52-56. As illustrated in **Scheme 1C**, BRD4 degradation not only suppresses PD-L1 expression on cancer cells 57, alleviating T cell exhaustion, but also induces immunogenic cancer cell death (ICD). Recent studies have elucidated that BRD4 degradation triggers a transcriptional breakdown of the c-Myc axis, which subsequently induces acute endoplasmic reticulum (ER) stress and upregulates Death Receptor 5 (DR5) 13, 52, 55, 58-60. This sequential BRD4-c-Myc-ER stress-DR5 signaling axis serves as a critical bridge to ICD, characterized by the release and exposure of damage-associated molecular patterns (DAMPs), including high-mobility group box 1 (HMGB1), adenosine triphosphate (ATP), and calreticulin (CRT) 61. These DAMPs promote dendritic cell maturation and antigen presentation, leading to enhanced T cell activation and infiltration into tumor tissues. Thus, BRD4 degradation facilitates both intrinsic oncogenic suppression and extrinsic immune activation, collectively reshaping the TME toward an immunostimulatory phenotype as a monotherapy. Taken together, this study introduces potential Alb-TAC, a structurally optimized esterase-responsive albumin-binding PROTAC that integrates targeted BRD4 protein degradation at targeted tumor tissues. In this work, the albumin-binding efficiency, esterase-responsive cleavage kinetics, and BRD4 degradation capability of Alb-TAC candidates were systematically investigated *in vitro* to elucidate their pharmacological activation profile. Furthermore, the pharmacokinetic behavior, tumor-targeting efficacy, and *in vivo* therapeutic performance of the optimized Alb-TAC were comprehensively evaluated in CT26 tumor-bearing mice, confirming its potent antitumor and immunomodulatory activity.

## Methods

### Reagents

Common reagents, solvents, fluorescent probes, and antibodies (anti-BRD4, anti-c-Myc, anti-Bcl-2, and anti-β-actin) were identical to those reported in our previous study 39. Reagents newly employed in this work are listed below. Triethylamine (TEA; 99.5%), 1-(2-aminoethyl)maleimide hydrochloride, and succinic acid (99%) were purchased from Sigma-Aldrich (St. Louis, MO, USA) and Tokyo Chemical Industry (TCI; Toshima, Tokyo, Japan), respectively. Anti-PD-L1 antibody was obtained from Abcam (Cambridge, UK). Penicillin and streptomycin were included in culture media sourced from WELGENE Inc. (Daegu, Republic of Korea). CT26 mouse colorectal carcinoma cells were obtained from ATCC (Manassas, VA, USA). Female BALB/c mice (5 weeks old) were purchased from NaraBio, Inc. (Seoul, Republic of Korea). All reagents were used as received.

### Synthesis of albumin-binding PROTACs (Alb-TAC#1 and Alb-TAC#2)

**Alb-TAC#1:** Alb-TAC#1 was synthesized according to our previously reported two-step procedure 39. Briefly, ARV-771 was first conjugated with 5-azidovaleric acid *via* Steglich esterification to yield the azide-functionalized intermediate (N_3_-PROTAC), which was subsequently reacted with BCN-PEG_2_-Mal through copper-free click chemistry. The final product was purified by preparative RP-HPLC and characterized by LC-MS, confirming identical purity and molecular weight to those reported previously.

**Alb-TAC#2:** Succinic acid (23.94 mg, 0.20 mmol) was weighed into an Eppendorf tube and solubilized in 250 µL of THF. DMAP (61.91 mg, 0.51 mmol) and EDC-HCl (78.67 mg, 0.51 mmol) were combined in DCM (500 μL) and agitated on a shaker at ambient temperature for 10 min. A solution of ARV-771 (20 mg, 0.02 mmol) in DCM (250 μL) was subsequently introduced. After stirring for 1 h, the crude reaction mixture was evaporated and subjected to C18 column purification (Sep-Pak C18 cartridges 5 g; Waters Corporation, Milford, MA, USA) with an eluent of ACN/H_2_O (70/30, *v/v*) to obtain the acid PROTAC, and then the isolated acid-PROTAC was subsequently freeze-dried. For the synthesis of Alb-TAC, acid PROTAC (22.03 mg, 0.02 mmol) and HATU (11.56 mg, 0.03 mmol) were dissolved in 500 µL of DMF. DIPEA (14.16 µL, 0.08 mmol) was then introduced, and the solution was agitated for 15 min. The maleimido amine-HCl salt was subsequently introduced, and the coupling reaction proceeded at ambient temperature for 1 h. After completion, the mixture was purified on a C18 preparative RP-HPLC column eluted with ACN/H_2_O (70/30, *v/v*), and the collected fractions were freeze-dried to afford pure Alb-TAC. Each intermediate and final product was characterized by RP-HPLC (1200 Series, Agilent Technologies, Palo Alto, CA, USA) and LC-MS (Q-TOF 5600, AB SCIEX, Framingham, MA, USA).

### Molecular docking of albumin-binding PROTAC with PLE5

The 3D structure of pig liver esterase (PLE5, PDB ID: 5FV4) was obtained from the RCSB Protein Data Bank and prepared using UCSF Chimera (version 1.18) by removing water molecules, assigning atomic charges, and adding hydrogen atoms. Ligand structures were drawn in ChemDraw, energy-minimized with the MMFF94 force field, and converted to PDBQT format using Open Babel (version 3.1.0) for subsequent docking simulations. Docking was conducted using Autodock Vina (version 1.1.2) with a grid box measuring 45 × 55 × 52 Å, centered on the active site. Numerous poses were evaluated utilizing the default parameters. For each ligand, the highest-ranking pose was chosen, and the docking score (expressed as ΔG in kcal/mol) was recorded. The theoretical inhibition constant (Ki) was determined from the binding free energy ΔG and is represented in molarity (M). The root-mean-square deviation (RMSD) between each docked position and the reference ligand conformation was computed to evaluate pose reliability.

### Physiochemical characterization and albumin conjugation of albumin-binding PROTACs

Albumin-binding kinetics of Alb-TAC#1 and Alb-TAC#2 were evaluated using a time-dependent RP-HPLC assay. Briefly, BSA (100 µM in PBS) was mixed with each Alb-TAC candidate (70 µM in PBS) at a 1:1 (*v/v*) ratio and incubated at 37 ℃ under gentle agitation. At predetermined time points (0, 5, 30, and 60 min), the reaction was quenched by adding 4-maleimidobutyric acid to block remaining free thiols. Each sample was immediately analyzed by RP-HPLC using a gradient elution mode of ACN/H_2_O (70/30, *v/v*) over 30 min. The albumin-binding rate was quantified by monitoring the decrease in the Alb-TAC peak area and the concurrent appearance of the broadened BSA-conjugate peak over time.

The molecular weight of BSA, BSA-conjugated Alb-TAC#1, and BSA-conjugated Alb-TAC#2 was analyzed using a matrix-assisted laser desorption/ionization time of flight (MALDI-TOF, AB Sciex TOF/TOF 5800 System, USA) with cyano-4-hydrocinnamic acid (CHCA) matrix after incubating BSA (100 µM in PBS) with Alb-TAC candidate (70 µM) at a 1:1 (*v/v*) ratio. Hydrodynamic diameters of unconjugated and Alb-TAC-bound BSA (0.5 mg/mL) were recorded in saline at 37 ℃ by DLS (Zetasizer Nano ZS, Malvern Instruments, Worcestershire, UK). The morphology of the Alb-TAC candidate was observed in distilled water (5 mM) *via* transmission electron microscopy (TEM) using Talos F200X (Thermo Fisher Scientific, Waltham, MA, USA). For TEM sample preparation, a drop of colloidal solution was applied onto 200 square mesh copper grids with a carbon film (Electron Microscopy Science, PA, U.S.A.), followed by negative staining with a 2% w/v uranyl acetate solution, and then air-dried for 1 h. TEM images were captured using a charge-coupled device (CCD) camera and imaging software.

Esterase-mediated hydrolysis of Alb-TAC#1 and Alb-TAC#2, along with the concomitant liberation of parent ARV-771, was examined under cell-free conditions. To generate albumin-conjugated Alb-TAC, each candidate was combined with albumin at equimolar ratio in deionized water and allowed to react at 37 ℃ for 1 h. Unreacted Alb-TAC was removed by 24 h dialysis (5 kDa MWCO) against methanol/water (1:1, *v/v*), and the retentate was freeze-dried to yield powdered albumin-Alb-TAC conjugate. Following 1 h of pre-equilibration at 37 ℃ in boric acid buffer (10 mM, pH 8.0), the conjugate solution (100 µM) was aliquoted (0.2 mL) and supplemented with porcine liver esterase (30 U/mL). Hydrolysis was tracked by withdrawing samples at 0, 1, 3, 6, and 12 h for RP-HPLC and LC-MS detection of released ARV-771.

Thiol-maleimide-specific conjugation between Alb-TAC and albumin was evaluated by RP-HPLC. BSA (100 µM in PBS) was mixed with Alb-TAC (70 µM in PBS) at a 1:1 (*v/v*) ratio and incubated at 37 ℃. Four conditions were prepared: Alb-TAC alone, BSA alone, Alb-TAC + BSA, and Alb-TAC + BSA pretreated with a thiol-blocking reagent. For thiol blocking, BSA was incubated with 4-maleimidobutyric acid (1:3 molar ratio) for 30 min prior to Alb-TAC addition. Samples were analyzed on a C18 column using a linear gradient of ACN/H_2_O (70/30, *v/v*) over 30 min. Albumin binding was quantified by monitoring the disappearance of the Alb-TAC peak and the corresponding broadening/shift of the BSA peak. Additionally, LC-MS spectra of free BSA and BSA-bound Alb-TAC were compared to confirm the binding ability of Alb-TAC.

For assessment of thiol-selective albumin conjugation, a Cy5.5-maleimide probe was prepared by coupling Cy5.5-NHS ester with maleimido amine-HCl (equimolar, 2 h, RT). BSA (100 μM in PBS) was then reacted with Cy5.5-maleimide (70 μM) at equimolar ratio for 1 h at RT to form the Alb-Cy5.5-maleimide conjugate. As a control, Alb-Cy5.5-maleimide was pretreated with 4-maleimidobutyric acid at a 1:3 molar ratio to block maleimide conjugation sites prior to reaction. Samples were analyzed *via* native 10% SDS-PAGE gel. The gels were observed by trans-UV using the iBright^TM^ Imaging System (Invitrogen; Waltham, Massachusetts, U.S.) and then stained with Coomassie blue for visualizing proteins.

To evaluate the drug-release profile, Alb-TAC was first covalently conjugated to BSA under the conditions described above. Briefly, Alb-TAC was incubated with BSA at 37 ℃ for 1 h to generate albumin-Alb-TAC conjugates. The resulting albumin-Alb-TAC conjugate solution was then divided into two groups. For enzymatic activation, one portion received esterase (30 U/mL), while the other served as an untreated (intact conjugate) control. Free ARV-771 was used as an additional control. Subsequently, intact albumin-Alb-TAC conjugates, esterase-treated albumin-Alb-TAC conjugates, and free ARV-771 were loaded into dialysis tubes (25 kDa MWCO) and incubated at 37 ℃ under gentle agitation. The cumulative appearance of ARV-771 in the dialysate was quantified by HPLC for up to 48 h.

To assess the long-term structural stability of Alb-TAC under physiological conditions, mouse serum was used as a culture medium. First, whole blood was collected by cardiac puncture form 6-week-old male BALB/c mice. The collected blood was allowed to clot for 30 min at room temperature and then centrifuged at 3,000 rpm for 15 min at 4 ℃ to obtain the supernatant (mouse serum). Alb-TAC (0.25 mM) was then added to freshly prepared mouse serum and gently stirred (100 rpm) at 37 ℃. The hydrodynamic diameter and size distribution of the Alb-TAC in mouse serum were measured using DLS at predetermined time points (0, 24, 48, 72, and 96 h). Simultaneously, the chemical integrity of Alb-TAC in mouse serum was evaluated by LC-MS at predetermined time points (0, 24, 48, 72, and 96 h) to detect peaks corresponding to Alb-TAC.

### *In vitro* cellular uptake and cell viability of Alb-TAC

Cellular internalization and cytotoxic activity of ARV-771 and Alb-TAC were evaluated in CT26 mouse colon carcinoma cells. Cells (1 × 10^5^) were plated onto 35 mm glass-bottom confocal dishes and maintained in RPMI 1640 for 24 h. Once confluent, cells were exposed to Cy5.5-conjugated ARV-771 (5 µM) or Cy5.5-labeled albumin-bound Alb-TAC (5 µM) at 37 ℃ for 1, 6, 12, and 24 h. Cells were rinsed three times with PBS, fixed in 4% formaldehyde (15 min), and counterstained with DAPI (10 min) for nuclear visualization. DAPI and Cy5.5 fluorescence were captured by CLSM to monitor time-resolved intracellular accumulation of each compound. For subcellular trafficking analysis, cells were incubated with Cy5.5-ARV-771 or Cy5.5-albumin-bound Alb-TAC (each 5 µM) at 37 ℃ over the same time course. At each interval, lysosomes were labeled by co-incubation with 2 µM LysoTracker^TM^ Red DND-99 (Thermo Fisher Scientific). After three PBS washes, cells were fixed (4% formaldehyde, 15 min) and nuclei were highlighted with DAPI (10 min). Multi-channel CLSM imaging of DAPI, LysoTracker (green), and Cy5.5 fluorescence was used to evaluate time-dependent uptake kinetics and lysosomal co-localization patterns. Intracellular entry of albumin-bound Alb-TAC and subsequent esterase-triggered release of free ARV-771 were verified as follows. CT26 cells (1 × 10^5^ per well) were seeded in 6-well plates, allowed to attach for 24 h, and then treated with albumin-conjugated Alb-TAC (5 µM). At 3, 6, 12, and 24 h, cells were collected and lysed in RIPA buffer containing protease inhibitor cocktail (100:1, v/v). Cell extracts were then subjected to LC-MS analysis.

Dose-response cytotoxicity was determined by the CCK-8 assay. CT26 cells were seeded into 96-well plates at a density of 3 × 10^3^ cells/well and maintained at 37 ℃ under 5% CO_2_ for 24 h. Cells were subsequently exposed to serial concentrations (0.001-200 µM) of ARV-771 or Alb-TAC for 48 h. Following treatment, cells were exposed to 10% CCK-8-containing culture media for 30 min, and viability of cells was measured using a microplate reader. The dose-response curves were plotted, and IC50 values were determined using GraphPad Prism 8 software (GraphPad Software, San Diego, CA, USA). The Alb-TAC-induced apoptosis was assessed by Annexin V/PI staining. CT26 cells (1 × 10^6^ per well) were seeded in 6-well plates and allowed to adhere for 24 h. Cells were then treated with Alb-TAC or ARV-771 (5 µM) for 48 h. Staining was performed by incubating cell suspensions with Annexin V (10 µL) and PI (5 µL) in 200 µL of binding buffer for 20 min at 37 ℃ in the dark. After the addition of 400 µl binding buffer, samples were acquired within 1 h using flow cytometry (BD FACSVerse, BD bioscience, USA) and analyzed using the FlowJo software. Live/Dead viability assay was further assessed. CT26 cells (2 × 10^5^ per well) were plated in 6-well plates and stabilized for 24 h, then treated with 5 µM of Alb-TAC or ARV-771 for 48 h. After PBS washes, cells were incubated with calcein-AM (live) and 7-aminoactinomycin D (7-AAD; dead) for 30 min. Fluorescence images were obtained using a confocal laser scanning microscope with identical acquisition settings across groups.

### Western blot analysis

The degradation of BRD4 and its downstream targets, including PD-L1, c-Myc, and Bcl-2, following treatment with ARV-771 or Alb-TAC, was evaluated by Western blot analysis. CT26 cells (1 × 10^5^ cells per well) were seeded in 6-well plates and cultured at 37 ℃ and 5% CO_2_ for 24 h. For concentration-dependent degradation, cells were treated with increasing concentrations of ARV-771 or Alb-TAC (0, 0.1, 0.5, 1, 5, and 10 µM) for 24 h. For time-dependent degradation, cells were treated with a fixed concentration of 10 µM ARV-771 or Alb-TAC and harvested at predetermined time points (0, 1, 3, 6, 24, and 48 h). After treatment, cells were washed three times with PBS and lysed with 1% protease-containing lysis buffer. Lysates were centrifuged at 12,000 rpm for 24 min at 4 ℃, and supernatants were collected for protein quantification using a BCA assay. Equal amounts of protein were resolved on 6% SDS-polyacrylamide gels and transferred onto nitrocellulose membranes. Membranes were blocked with 5% BSA in TBS containing 0.1% Tween-20 (TBS-T) for 1 h and incubated overnight at 4 ℃ with primary antibodies against BRD4, PD-L1, c-Myc, and Bcl-2 (1:1000 dilution). After washing three times with TBS-T, membranes were incubated with HRP-conjugated secondary antibodies and developed using an enhanced chemiluminescence (ECL) system. Band intensities were quantified to assess the degradation kinetics of BRD4 and its downstream proteins.

To verify that Alb-TAC-induced BRD4 degradation occurs through the ubiquitin-proteasome pathway, CT26 cells were co-treated with the VHL inhibitor VH298 (50 µM), proteasome inhibitor Bortezomib (10 nM), or NAE inhibitor Pevonedistat (5 µM), which blocks cullin-dependent ubiquitination. CT26 cells (1 × 10^5^ cells per well) were seeded in 6-well plates and cultured for 24 h at 37 ℃ under 5% CO_2_. Subsequently, cells were treated with 10 µM Alb-TAC in the presence or absence of each inhibitor and harvested at predetermined time points (0, 1, 3, 6, 24, and 48 h). After treatment, cells were washed with PBS and lysed with 1% protease-containing lysis buffer. The lysates were centrifuged at 12,000 rpm for 24 min at 4 ℃, and the supernatants were analyzed by Western blotting as described above. Membranes were probed with primary antibodies against BRD4 and PD-L1 to evaluate the attenuation of Alb-TAC-induced degradation upon inhibition of each pathway.

### *In vitro* tumor microenvironment profiling of Alb-TAC

To determine whether Alb-TAC treatment induced immunogenic cell death (ICD), damage-associated molecular pattern (DAMP) markers were analyzed. CT26 cells (1 × 10^5^) were selected into 35-mm glass-bottom dishes and allowed to adhere overnight. Cells were then treated with ARV-771 (10 µM) or Alb-TAC (10 µM) and incubated for 24 h at 37 ℃ under 5% CO_2_. For the detection of surface-exposed calreticulin (CRT), cells were incubated with Alexa Fluor 647-conjugated anti-CRT antibodies at 4 ℃ for 1 h. After staining, cells were washed three times with PBS, fixed with 4% paraformaldehyde for 15 min, and their nuclei were stained with DAPI for 10 min. CRT expression was visualized by confocal laser scanning microscopy (CLSM). Quantitative analysis of surface CRT expression was performed by flow cytometry using a BD FACSVerse (BD bioscience, USA). The culture supernatants were collected after ARV-771 or Alb-TAC treatment and analyzed for extracellular ATP and HMGB1 release. ATP levels were quantified using an ATP luminescence assay kit according to the manufacturer's protocol, and HMGB1 expression was assessed by Western blot analysis.

To evaluate the immunomodulatory effects of Alb-TAC on the tumor microenvironment, bone marrow-derived macrophages (BMDMs) and dendritic cells (BMDCs) were generated from bone marrow cells isolated from 6-week-old male BALB/c mice. For BMDM differentiation, bone marrow cells were cultured in RPMI-1640 medium supplemented with 10% (*v/v*) FBS, 1% penicillin-streptomycin, and M-CSF (20 ng/mL) for 7 days, with cytokines replenished on days 1, 2, 3, 4, and 6, and half of the medium replaced on days 4 and 6. For BMDC differentiation, cells were cultured for 8 days in the presence of GM-CSF (20 ng/mL), IL-4 (20 ng/mL), and 0.1% β-mercaptoethanol under identical base media conditions. To assess dendritic cell maturation in response to Alb-TAC-induced immunogenic cell death (ICD), CT26 cells (1 × 10^6^) were seeded into 100-mm dishes and treated with ARV-771 or Alb-TAC (10 µM, ARV-771 equiv.) for 24 h. Following treatment, CT26 cells were co-cultured with BMDCs for 24 h, and the proportion of mature dendritic cells (CD11c^+^ CD40^+^ CD86^+^) was quantified by flow cytometry. Similarly, to evaluate macrophage polarization, CT26 cells treated under the same conditions were co-cultured with BMDMs for 24 h. The proportion of M1-like macrophages (CD45^+^ F4/80^+^ CD86^+^) was analyzed using flow cytometry.

For the phagocytosis assay, CT26 cells (1 × 10^6^) were seeded in 100-mm dishes and treated with 10 µM ARV-771 or Alb-TAC for 24 h. After treatment, CT26 cells were labeled with pHrodo^TM^ Deep Red dye to visualize engulfment, while BMDMs were stained with CellTracker^TM^ Green CMFDA dye according to the manufacturer's instructions. Labeled macrophages and cancer cells were co-cultured at a 1:3 ratio (1 × 10^5^ BMDMs: 3 × 10^5^ CT26 cells) for 2 h at 37 ℃. Following incubation, cells were washed gently with PBS and fixed with 4% paraformaldehyde for 15 min. Phagocytosis was visualized using CLSM, and the extent of CT26 cell uptake by BMDMs was quantified based on the overlap of red (cancer cell) and green (macrophage) fluorescence signals.

### *In vivo* tumor targeting ability of Alb-TAC in CT26 tumor-bearing tumor models

All animal experiments assessing the tumor-targeting, retention, and theragnostic effects of Alb-TAC were conducted in accordance with the Institutional Animal Care and Use Committee (IACUC) guidelines of Ewha Womans University (approval no. EWHA IACUC 22-073-7). All mice were fed and bred under specific-pathogen-free (SPF) facilities at the College of Pharmacy, Graduate School of Pharmaceutical Sciences, Ewha Womans University. To evaluate the effects of albumin binding on systemic circulation and pharmacokinetic profiles, *in vivo* pharmacokinetic studies were performed using tumor-free BALB/c mice (6-week-old, male, n = 3 per group). Mice were randomly assigned to receive a single intravenous injection *via* the tail vein of Cy5.5-labeled ARV-771, Cy5.5-labeled Alb-TAC#1, or Cy5.5-labeled Alb-TAC#2 at an equiv. dose of 10 mg/kg (equiv. to ARV-771). Blood samples were collected in heparinized tubes at predetermined time points (20 and 40 minutes and 1, 3, 6, 24, 48, 72, and 96 hours) after injection. Plasma was separated by centrifugation at 3,000 rpm for 15 min at 4 ℃. The fluorescence intensity of Cy5.5 in plasma was measured using a microplate reader. Pharmacokinetic parameters, including plasma half-life (*t*_1/2_), area under the curve (AUC), and peak plasma concentration (*C_max_*)were calculated based on the fluorescence-time profiles. To evaluate the *in vivo* biodistribution and tumor-targeting properties of Alb-TAC, CT26 tumor-bearing BALB/c mice were used. Six-week-old male BALB/c mice were subcutaneously inoculated with CT26 colon carcinoma cells (1 × 10^6^ cells) into the left thigh. Tumor growth was monitored until tumor volumes reached approximately 150-200 mm³. For longitudinal *in vivo* biodistribution and tumor-targeting analysis, mice were randomly assigned to receive a single intravenous injection of Cy5.5-labeled Alb-TAC or Cy5.5-labeled ARV-771 at an equivalent dose (3 mg/kg, equiv. to each compound; n = 3 per group). Whole-body NIRF imaging was performed at predetermined time points (0, 1, 3, 6, 9, 24, 48, 72, and 96 h post-injection) using an IVIS Lumina Series III imaging system (PerkinElmer, Waltham, MA, USA). Tumor-associated fluorescence signals were quantified using Living Image software (PerkinElmer) to assess time-dependent tumor accumulation and systemic retention profiles.

To corroborate the *in vivo* imaging results and avoid interference with longitudinal imaging, *ex vivo* biodistribution studies were performed using an independent cohort of CT26 tumor-bearing mice (n = 3 per group). Mice were intravenously administered Cy5.5-labeled Alb-TAC or Cy5.5-labeled ARV-771 at the same dose (3 mg/kg) and euthanized at 24 h post-injection. Major organs (heart, liver, lung, spleen, and kidney) and tumor tissues were harvested and subjected to *ex vivo* fluorescence imaging using the IVIS Lumina Series III system. Mean fluorescence intensities in each organ and tumor were quantified using Living Image software to compare biodistribution and tumor-specific accumulation between treatment groups. For intratumoral distribution analysis, excised tumor tissues from the *ex vivo* cohort were immediately embedded and cryosectioned. Cryosectioned tumor tissues were first directly imaged by confocal laser scanning microscopy to visualize Cy5.5 fluorescence and assess intratumoral distribution patterns. Subsequently, the same cryosectioned tumor sections were immunostained with an anti-CD31-FITC antibody to label endothelial cells and counterstained with DAPI to visualize nuclei. Confocal fluorescence images were acquired to evaluate the spatial relationship between drug distribution and tumor vasculature. Cross-sectional fluorescence intensity profiling was performed to assess the extent of interstitial diffusion beyond CD31-positive vascular regions.

### *In vivo* therapeutic efficacy of Alb-TAC against tumor-bearing mice

Antitumor efficacy of ARV-771 and Alb-TAC was assessed in CT26 tumor-bearing mice. CT26 cells (1 × 10^6^) were implanted subcutaneously into the left flank of each mouse. Once tumors reached approximately 50 ± 20 mm^3^, animals were randomly allocated to five groups (n = 5 each): saline, ARV-771 (10 mg/kg), Alb-TAC at 10 mg/kg ARV-771 equivalent (early-response cohort, sacrificed on day 15), Alb-TAC at 5 mg/kg, and Alb-TAC at 10 mg/kg ARV-771 equivalent (dose-response and extended-monitoring cohort through day 28). Drugs were injected intravenously on a 3-day cycle (days 0, 3, 6, and 9), with tumor dimensions and body weights recorded every other day. On day 15, the early-evaluation cohort (saline, ARV-771, Alb-TAC 10 mg/kg) was euthanized; tumors and vital organs (liver, heart, lung, kidney, and spleen) were harvested for downstream analyses. The two remaining Alb-TAC cohorts (5 and 10 mg/kg) continued to be monitored without further dosing until day 28 to evaluate durability of the therapeutic response. Excised day-15 tumors were photographed for gross size comparison and processed for Western blot (BRD4, PD-L1, Bcl-2, and c-Myc), TUNEL apoptosis staining, and immunofluorescence labeling of BRD4 and PD-L1. Vital organs and tumors were additionally sectioned and stained with H&E to examine possible histopathological abnormalities.

### *In vivo* tumor microenvironment profiling

To investigate the enhancement of immune response induced by the Alb-TAC, tumor tissues were harvested from CT26 tumor-bearing mice on day 7 following treatment initiation. Single-cell suspensions were prepared using a tumor dissociation kit, and the isolated cells were subsequently counted. To prevent nonspecific antibody binding, the cells were incubated with Fc receptor blocking reagent for 10 min at 4 ℃ prior to staining. To access immunogenic cell death (ICD)-related damage-associated molecular patterns (DAMPs), tumor lysates were analyzed by Western blot using anti-HMGB1 antibodies. For flow cytometric analysis, multi-parameter staining was performed at 4 ℃ for 60 min to characterize immune and tumor cell subsets within the tumor microenvironment. Data were acquired using a flow cytometry (BD FACSVerse, BD bioscience, USA) and analyzed using the FlowJo software. For all samples, viable mononuclear cells were first identified using FSC-H versus SSC-H parameters, effectively discriminating intact cells from debris. Subsequent gating was tailored for each target population as follows. First, for the PD-L1-expressing tumor cells, singlets were isolated using SSC-A versus SSC-H within the viable population. Tumor cells were identified as the CD45-negative (CD45^-^) fraction, and the expression of PD-L1 (PD-L1^+^) was quantified within this population. Next, for the cytotoxic T cells (CD8^+^ T cells), leukocytes were identified as CD45-positive (CD45^+^) after selecting viable singlets *via* FSC-A versus FCS-H. Total T cells were gated as CD3-positive (CD3^+^), and cytotoxic T cells were subsequently defined as CD8-positive (CD8^+^). Moreover, for the regulatory T cells (Tregs), T lymphocytes were identified as CD3-positive (CD3^+^) following the initial viable cell gate (FSC-H/SSC-H). Within the T cell population, T cells were gated as CD4-positive (CD4^+^), and Tregs were finally defined as the CD25-positive (CD25^+^) subset. Finally, the dendritic cells (DCs) were identified as CD11c-positive (CD11c^+^) from the viable mononuclear gate. The maturation status was assessed by sequential gating of co-stimulatory markers, defined as the CD40-positive (CD40^+^) and CD86-positive (CD86^+^) population.

To evaluate the systemic immune response and functional cytokine profiling, blood samples were collected from CT26 tumor-bearing mice on day 7 following treatment initiation. Plasma was separated from whole blood by centrifugation at 3,000 rpm for 15 min at at 4 ℃. The concentrations of key cytokines in the plasma, including TGF-β (LEGEND MAX^TM^ Mouse Latent TGF-β ELISA Kit, BioLegend), IL-6 (Mouse IL-6 ELISA Kit, Invitrogen), and IFN-γ (LEGEND MAX^TM^ Mouse IFN-γ ELISA Kit, BioLegend), were quantified using the respective enzyme-linked immunosorbent assay (ELISA) kits according to the manufacturers' instructions. Absorbance was measured using a microplate reader, and cytokine concentrations were determined based on standard curves.

### Systemic toxicity of AlbTAC in CT-26 tumor-bearing mice

To assess systemic safety, blood samples were collected from the orbital sinus on day 15 prior to sacrifice, and hematological and serum biochemical parameters were analyzed. Hematology included measurements of total white blood cell (WBC) count, red blood cell (RBC) count, hemoglobin (HGB), and hematocrit (HCT). Serum biochemical analysis included assessment of hepatic function markers (aspartate aminotransferase, AST; alanine aminotransferase, ALT; and alkaline phosphatase, ALP) and the renal function marker (blood urea nitrogen, BUN). All parameters were measured using an automated hematology analyzer (AU480, Beckman Coulter, USA), and results were compared with normal physiological reference ranges to evaluate potential Alb-TAC-induced toxicity.

### Statistical analysis

Results are expressed as mean ± SD of at least three independent experiments. Two-group comparisons were made using Student's t-test, while multi-group comparisons were performed by one-way ANOVA followed by Tukey-Kramer post-hoc analysis. Significance thresholds are denoted in figures as *p < 0.05, **p < 0.01, ***p < 0.001, and ****p < 0.0001.

## Results and Discussion

### Synthesis of esterase-responsive albumin-binding BRD4-degrading PROTACs (Alb-TACs)

**Figure 1:**
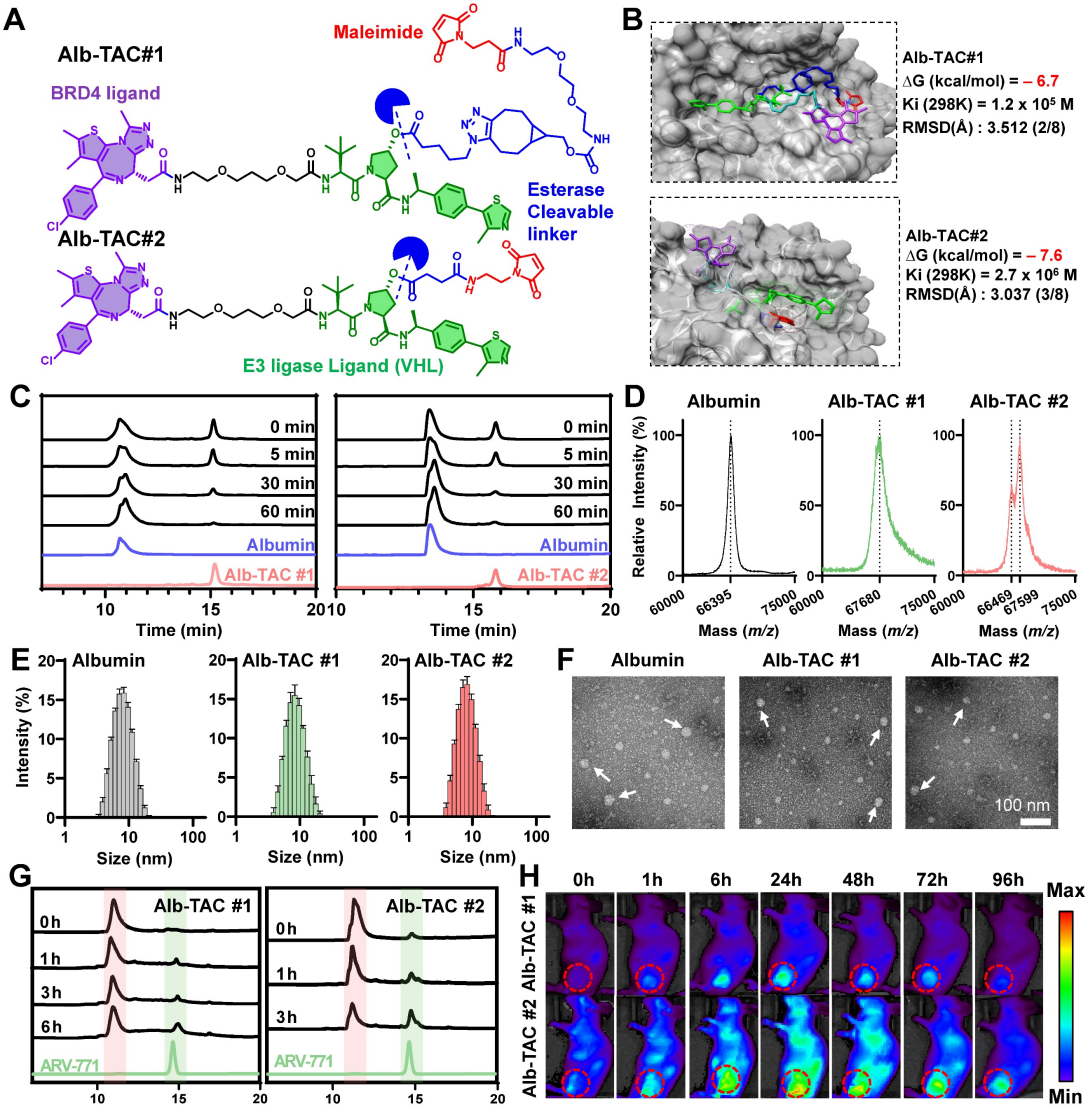
** Comparative design and characterization of albumin-binding BRD4 PROTACs (Alb-TAC #1 and Alb-TAC #2).** (A) Chemical structures of Alb-TAC#1 and Alb-TAC#2 showing the BRD4 ligand (purple), VHL E3-ligase ligand (green), esterase-cleavable linker (blue), and maleimide for *in situ* albumin binding (red). (B) Molecular docking and their binding parameters of Alb-TAC #1 or Alb-TAC #2 with esterase. (C) Reverse-phase HPLC (RP-HPLC) analysis of Alb-TAC#1 and Alb-TAC#2 incubated with BSA to evaluate thiol-dependent albumin conjugation kinetics. (D) MALDI-TOF mass spectra of BSA (66.5 kDa) and BSA incubated with Alb-TAC #1 or Alb-TAC #2 (1:1 *v/v* ratio). (E) Hydrodynamic diameters of BSA, BSA-Alb-TAC #1, and BSA-Alb-TAC #2 measured by dynamic light scattering (DLS) at 37 ℃. (F) Transmission electron microscopy (TEM) images of BSA, BSA-Alb-TAC #1, and BSA-Alb-TAC #2 (scale bar, 100 nm). (G) Time-course HPLC analysis of Alb-TAC#1 and Alb-TA #2 under esterase treatment conditions (30 U/mL). (H) Non-invasive NIRF images of the whole body of CT26-bearing xenograft mice (n = 3 per group) intravenously injected with Cy5.5-labeled Alb-TAC #1 or Alb-TAC #2. (In (G), the dotted red circles indicate the tumor region.)

To investigate how linker architecture influences albumin conjugation efficiency, esterase accessibility, and pharmacokinetic behavior, two PROTAC candidates, Alb-TAC#1 and Alb-TAC#2, were designed with a BRD4-targeting ligand (purple), a VHL-based E3 ligase ligand (green), a maleimide moiety for Cys34-selective albumin binding (red), and structurally distinct esterase-cleavable linkers (blue) (**Figure 1A**). The key structural distinction lies in the linker architecture, enabling a direct assessment of differences in esterase-responsive cleavage and albumin conjugation efficiency, respectively. To establish an albumin-mediated delivery platform for the BRD4-degrading PROTAC, ARV-771, two esterase-responsive albumin-binding linkers of bicyclononyne-polyethylene glycol-maleimide (BCN-PEG_2_-Mal) and N-(2-aminoethyl)maleimide (AE-Mal) were employed. Alb-TAC#1 was constructed *via* a two-step strategy involving Steglich esterification of ARV-771 with 5-azidovaleric acid to yield azide-functionalized intermediate (N_3_-PROTAC), followed by copper-free click conjugation with BCN-PEG_2_-Mal to yield ARV-771-BCN-PEG_2_-Mal (Alb-TAC#1) (**Figure S1**). In parallel, Alb-TAC#2 was generated by first producing an acid-functionalized ARV-771 intermediate (acid-PROTAC) through EDC/DMAP-mediated esterification with succinic acid, which was subsequently conjugated to N-(2-aminoethyl)maleimide *via* amide bond formation, resulting in ARV-771-AE-Mal (Alb-TAC#2) (**Figure S2**). Both crude products were purified using C18 reverse-phase column chromatography and characterized by HPLC and LC-MS. This confirmed the successful synthesis of Alb-TACs with high chemical purity (Alb-TAC#1: 62.9% yield, 98.2% purity; Alb-TAC#2: 81.5% yield and 98.85% purity). The observed *m/z* values (Alb-TAC#1: 794 [M + 2H]^2+^, 1587 [M + H]^+^; Alb-TAC#2: 604 [M + 2H]^2+^, 1208 [M + H]^+^, 1230 [M + Na]^+^) closely matched their calculated molecular weights (Alb-TAC#1: 1587.32 Da and Alb-TAC#2: 1208.85 Da), thereby validating accurate molecular construction (**Figure S3**). Collectively, these results confirmed the successful synthesis and molecular characterization of two structurally distinct esterase-responsive albumin-binding PROTAC candidates, establishing a robust platform for their subsequent comparative evaluation. To assess whether structural variations surrounding the ester bond of Alb-TACs can affect esterase-mediated recognition and hydrolysis, molecular docking simulations were performed in the presence of esterase (**Figure 1B**). The results indicated that Alb-TAC#1 exhibited a binding free energy of ∆G = -6.7 kcal/mol and Ki (298 K) = 1.2 × 10^5^ M, and RMSD = 3.512 Å for esterase. In contrast, Alb-TAC#2 showed a stronger binding affinity with ∆G = -7.6 kcal/mol and Ki (298 K) = 2.7 × 10^6^ M, and RMSD = 3.037 Å for esterase. This difference is likely driven by steric effects, as the bulky BCN moiety in Alb-TAC#1 may hinder productive accommodation of the ester bond near the catalytic residues, whereas the more streamlined linker in Alb-TAC#2 reduces such interference. These results suggest that the linker configuration in Alb-TAC#2 enables a more favorable fit within the esterase catalytic pocket, leading to enhanced binding stability and accessibility compared to Alb-TAC#1.

To empirically confirm these computational predictions, the albumin-binding and esterase-cleavage characteristics of Alb-TACs were subsequently investigated. As a first step, we evaluated the thiol-specific albumin binding of Alb-TACs in serum albumin solution. Serum albumin contains 35 cysteine residues, 34 of which are known to form disulfide bonds that are important for maintaining tertiary structure. Importantly, a single free thiol at Cys34 remains solvent-exposed and redox-active, accounting for approximately 80% of the free thiols in human plasma 62. Based on this feature, Alb-TAC was expected to covalently conjugate to albumin *in vivo* through maleimide-thiol chemistry at Cys34. To assess the albumin-binding properties of the two candidates, Alb-TAC#1 and Alb-TAC#2 were incubated with serum albumin at a 1:1 (*v/v*) ratio at 37 ℃. The chemical conjugation between albumin and Alb-TACs was subsequently analyzed by time-course HPLC analysis (**Figure 1C**). For both compounds, the HPLC peak corresponding to free Alb-TACs gradually decreased in a time-dependent manner, accompanied by a concomitant increase in a broadened peak attributed to albumin-Alb-TAC conjugates, which confirmed efficient albumin conjugation. Quantitative analysis of the free Alb-TAC peak area, expressed as a relative percentage of total Alb-TAC, revealed that Alb-TAC#2 exhibited a more pronounced reduction at early time points (5-30 min) compared with Alb-TAC#1 (**Figure S4**). Specifically, at 30 min, approximately 25% of free Alb-TAC#2 remained, whereas ~36% of free Alb-TAC#1 remained, clearly indicating faster conjugation kinetics for Alb-TAC#2. This faster conjugation rate of Alb-TAC#2 is likely attributable to the reduced steric hindrance and greater accessibility of the AE-Mal linker for albumin binding compared to the BCN-PEG_2_-Mal linker of Alb-TAC#1. To further validate the thiol-mediated covalent conjugation, MALDI-TOF mass spectrometry was performed following incubation of serum albumin (~66.5 kDa; 100 µM in PBS) with Alb-TAC#1 or Alb-TAC#2 (70 µM) at a 1:1 (*v/v*) ratio (**Figure 1D**). Native albumin showed a sharp peak at 66,395* m/z*, consistent with its theoretical molecular weight. Conjugation with Alb-TAC#1 or Alb-TAC#2 resulted in broader peaks at 67,680 and 67,599 *m/z*, respectively. These peaks were approximately 1,200 Da higher than native albumin, confirming successful 1:1 PROTAC conjugation *via* maleimide-thiol chemistry. To examine whether albumin binding of Alb-TACs affects the colloidal stability or induces large particle aggregation, dynamic light scattering (DLS) measurements were conducted in PBS at 37 ℃ (**Figure 1E**). The hydrodynamic diameters were 7.54 ± 0.10 nm for native albumin, 8.63 ± 0.12 nm for albumin-Alb-TAC#1, and 7.71 ± 0.12 nm for albumin-Alb-TAC#2. The slight increases in size confirmed successful binding without significant aggregation or structural destabilization. The monodisperse size distributions of Alb-TACs indicated preserved colloidal stability after conjugation. Morphological characteristics were further evaluated using transmission electron microscopy (TEM), which revealed predominantly dispersed nanoscale features with a size of approximately 5-10 nm, consistent with monomeric albumin-bound forms (**Figure 1F**). Occasional larger particles (indicated by white arrows) were observed and are attributed to drying-induced aggregation during TEM sample preparation, rather than intrinsic particle aggregation in solution. Collectively, these results demonstrate that both Alb-TAC#1 and Alb-TAC#2 efficiently conjugate to albumin under physiological conditions *via* maleimide-thiol chemistry while preserving colloidal stability and structural integrity.

We next compared the esterase-responsive cleavage behavior of Alb-TAC#1 and Alb-TAC#2. To mimic their expected circulating forms, albumin-bound Alb-TACs were subjected to time-course esterase treatment at 37 ℃, and the cleavage kinetics were subsequently analyzed by HPLC (**Figure 1G and S5**). Alb-TAC#1 underwent gradual hydrolysis, characterized by a stepwise reduction in the intact-compound peak over 6 h. This was accompanied by a corresponding increase in the free ARV-771 peak (~14.5 min), with near-complete conversion achieved by 6 h (~55.2% hydrolysis). In contrast, Alb-TAC#2 displayed markedly faster cleavage kinetics. The intact peak diminished rapidly within 3 h (~74.6% hydrolysis), while the ARV-771 peak was clearly observed with high intensity after 3 h post-incubation. These differences align with the molecular docking results, indicating that the AE-Mal-based linker in Alb-TAC#2 provides greater esterase accessibility due to reduced steric hindrance compared to the BCN-PEG_2_-Mal linker in Alb-TAC#1. Collectively, these *in silico* and *in vitro* results demonstrate that linker architecture critically governs esterase responsiveness. Alb-TAC#2, specifically, enables more efficient PROTAC release, thereby suggesting enhanced potential for intracellular activation within the tumor microenvironment. To determine whether these *in vitro* kinetic differences translated into distinct *in vivo* behaviors, we next evaluated the biodistribution and tumor-targeting properties of Cy5.5-labeled Alb-TAC#1 and Alb-TAC#2 in CT26 tumor-bearing mice. Non-invasive near-infrared fluorescence (NIRF) imaging was performed over 96 h following intravenous administration of each Alb-TAC (10 mg/kg) (**Figure 1H**). Both Alb-TACs exhibited detectable tumor accumulation; however, Alb-TAC#2 produced stronger and more persistent fluorescence signals throughout the observation period. Tumor-associated fluorescence of Alb-TAC#2 increased from 1 h, peaked at 24 h, and remained detectable for up to 96 h. In contrast, Alb-TAC#1 showed comparatively modest and transient accumulation in tumor tissues (**Figure S6**). *Ex vivo* fluorescence analysis at 24 h further confirmed these findings. Alb-TAC#2 accumulated in tumors at markedly higher levels, approximately two-fold greater mean fluorescence intensity, compared with Alb-TAC#1, but both Alb-TACs exhibited minimal accumulation in major normal organs (liver, lung, spleen, and heart) (**Figure S7**). In addition, a large amount of Alb-TAC#2 was accumulated in the kidney, wherein Alb-TAC#2 is slowly excreted in the urine due to the prolonged circulating property of Alb-TAC#2 in blood. *In vivo* pharmacokinetic (PK) studies further confirmed that both Alb-TAC#1 and Alb-TAC#2 exhibited significantly prolonged systemic circulation compared to free ARV-771 (**Figure S8**). The plasma half-lives (t_1/2_) of Alb-TAC#1 and Alb-TAC#2 were 15.48 and 15.58 h, respectively, representing an 11.64- and 11.71-fold increase compared to free ARV-771 (t_1/2_ = 1.33 h), which was rapidly cleared from circulation within a few hours. Furthermore, the total drug exposure (AUC) of Alb-TAC#1 and Alb-TAC#2 (219.17 and 219.20 µg/mL·h) was 18.85-fold higher than that of ARV-771 (11.63 µg/mL·h). Notably, Alb-TAC#2 exhibited a higher *C*_max_ (11.59 µg/mL) compared to Alb-TAC#1 (9.68 µg/mL) and ARV-771 (8.62 µg/mL), consistent with its superior tumor accumulation. These results identified Alb-TAC#2 as the optimized lead candidate due to its superior albumin conjugation dynamics, enhanced esterase accessibility, and improved tumor-targeting performance.

### *In vitro* esterase-responsive cytotoxicity of Alb-TAC in a cell culture system

**Figure 2:**
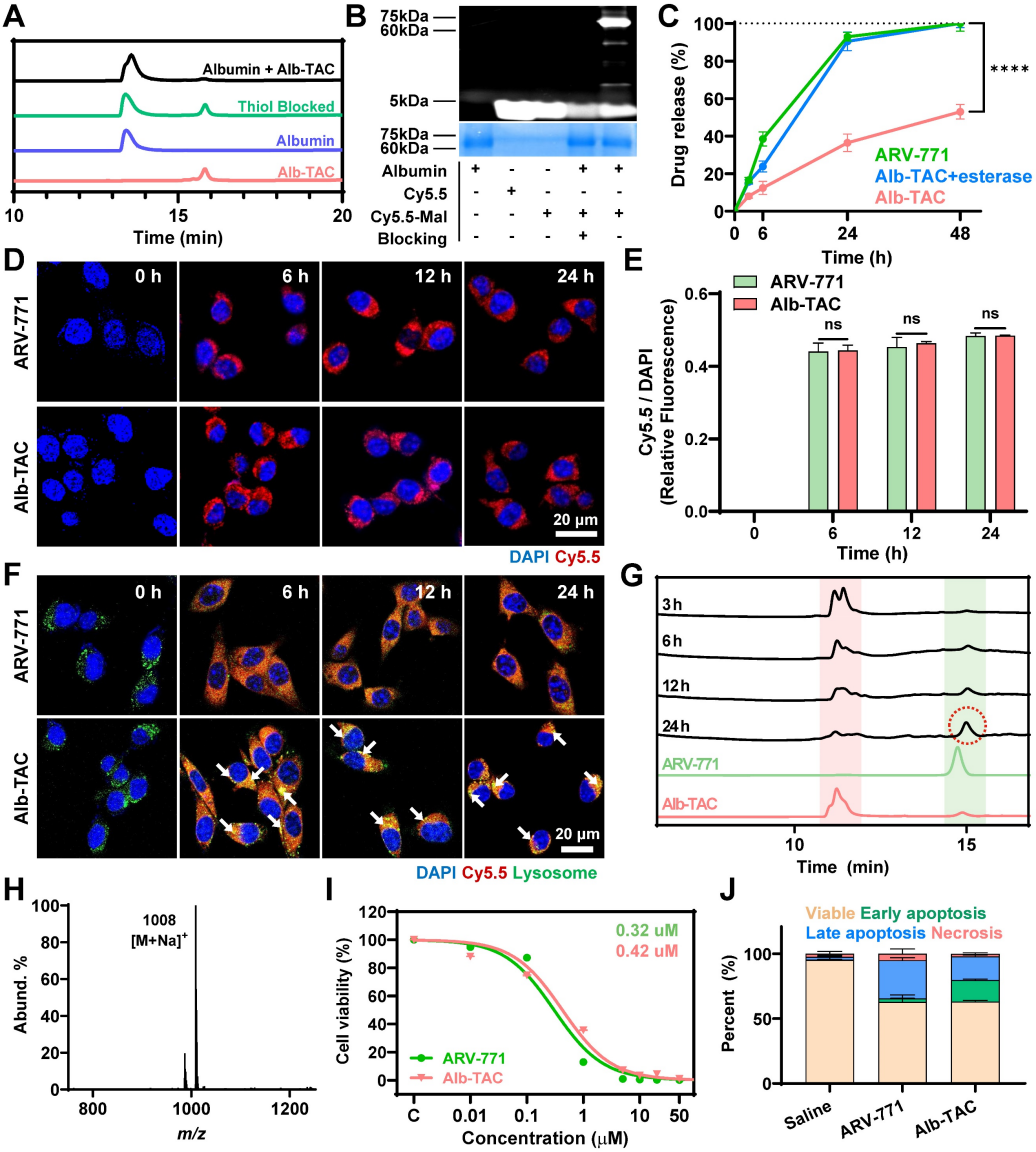
***In vitro* characterization of Alb-TAC.** (A) Reverse-phase HPLC (RP-HPLC) analysis of Alb-TAC incubated with BSA to evaluate thiol-maleimide-specific conjugation between Alb-TAC and BSA; a parallel condition used BSA pretreated with the thiol-blocking agent 4-maleimidobutyric acid to assess binding specificity. (B) Native PAGE analysis using a Cy5.5-labeled maleimide probe to examine thiol-maleimide conjugation between albumin and Alb-TAC. Samples included free Cy5.5, Cy5.5-maleimide, thiol-blocked albumin, and albumin incubated with Cy5.5-maleimide. Fluorescent and Coomassie-stained bands were visualized to assess conjugation specificity. (C) Drug release profile of Alb-TAC (intact), esterase-treated Alb-TAC, and free ARV-771 using dialysis (MWCO 25 kDa) at 37 ℃. (D, E) Confocal laser scanning microscopy (CLSM) images (D) and quantitative fluorescence analysis (E) of CT26 cells treated with Cy5.5-labeled ARV-771 or Cy5.5-labeled BSA-bound Alb-TAC over 24 h (scale bar, 20 μm). (F) CLSM images of CT26 cells treated with Cy5.5-labeled ARV-771 or Cy5.5-labeled Alb-TAC and co-stained with LysoTracker^TM^ over 24 h, showing intracellular co-localization with lysosomal compartments. The white arrows indicate the lysosomal co-localizations (yellow color) of Cy5.5-labeled Alb-TAC. (G) LC-MS chromatograms of CT26 cell lysates collected at 3, 6, 12, and 24 h following treatment with Alb-TAC (10 μM). The signal corresponding to released ARV-771 (red dotted circle) increased progressively over time and reached its maximum intensity at 24 h, confirming intracellular esterase-mediated cleavage of Alb-TAC. (H) LC-MS analysis of the 24 h peak (red-dotted circle on Figure 2G) showed a major ion at *m/z* 1008 [M + Na]^+^, matching the molecular weight of ARV-771 and confirming the identity of the released product. (I) Concentration-dependent curves for ARV-771 and Alb-TAC in CT26 cells over 48 h with half-maximal inhibitory concentrations (IC_50_) values. (J) Quantitative analysis of Annexin V/PI staining in CT26 cells after 48 h of treatment with 5 μM ARV-771 or Alb-TAC. Data are presented as mean ± SD. Statistical significance was determined by Student's t-test for (C, E) (ns, not significant; ****p < 0.0001).

Following the identification of Alb-TAC#2 (hereafter referred to as 'Alb-TAC') as the lead candidate, a series of biophysical and cellular assays were conducted to characterize its functional properties and therapeutic potential. Prior to these evaluations, the chemical structure of Alb-TAC was rigorously confirmed and verified by ^1^H and ^13^C NMR spectroscopy. In the ^1^H NMR spectrum (**Figure S9**), the maleimide proton was confirmed at 6.66 ppm (label k), while the methyl-bearing protons of the thiophene (labels e, f) and triazole (label d) moieties of JQ1 were observed at 2.3-2.8 ppm. The methyl protons of the thiazolidine ring of the VHL ligand appeared at 1.70 ppm (label c), and the tert-butyl protons were confirmed at 1.08 ppm (label a). In the ^13^C NMR spectrum (**Figure S10**), carbonyl carbons corresponding to the ester bond and maleimide were observed at 170.05-172.87 ppm (labels f, g, h), and the maleimide alkene carbon was confirmed at 134.37 ppm (label e). The methyl carbons of the thiophene moieties of JQ1 appeared at 11.39 and 11.54 ppm (labels a, b), while the triazole carbon of JQ1 and the methyl carbon of the VHL thiazolidine ring were observed at 14.56-14.81 ppm (labels c, d). The tert-butyl carbon of the VHL ligand was confirmed at 26.72 ppm (label i). To experimentally verify that Alb-TAC undergoes thiol-maleimide-specific conjugation with albumin, RP-HPLC analysis was performed using four samples: Alb-TAC alone, albumin alone, Alb-TAC incubated with albumin, and Alb-TAC incubated with albumin pretreated with a thiol-blocking reagent, respectively (**Figure 2A**). Free Alb-TAC produced a sharp peak at ~15-16 min, while native albumin showed its characteristic broad peak at an earlier retention time. When Alb-TAC was incubated with albumin (70 µM Alb-TAC: 100 µM albumin, 1:1 *v/v*) at 37 ℃, the Alb-TAC peak progressively diminished and ultimately disappeared. Concurrently, the free albumin peak broadened and exhibited a shifted retention profile, indicating covalent conjugation *via* thiol-maleimide chemistry. In contrast, when albumin was treated with the thiol blocking agent (4-maleimidobutyric acid), the Alb-TAC peak remained unchanged, and no alteration in the albumin peak was observed. This result confirms that conjugation does not occur in the absence of a free thiol group. Native PAGE analysis using a Cy5.5-labeled maleimide probe further corroborated these findings (**Figure 2B**). A strong fluorescence signal was observed at the albumin band only when incubated with Cy5.5-maleimide (lane 5), confirming thiol-maleimide conjugation. In contrast, lanes containing free Cy5.5 (lane 2) or Cy5.5-maleimide alone (lane 3) exhibited only background fluorescence, with no detectable Coomassie-positive albumin band. Pretreatment of albumin with a thiol blocker (lane 4) resulted in a Coomassie-positive albumin band but no fluorescence signal, verifying that the conjugation occurred specifically *via* the free thiol of albumin. Collectively, these results support the specificity and efficiency of Alb-TAC binding to albumin *via* thiol-maleimide conjugation under physiological conditions, corroborating the findings from HPLC analysis.

After establishing thiol-dependent albumin conjugation of Alb-TAC under physiological conditions, the next step was to determine whether the esterase-cleavable maleimide linker can enable drug release by the esterase-specific cleavage mechanism. For this purpose, Alb-TAC was first covalently conjugated to serum albumin, and the resulting albumin-Alb-TAC conjugates were subjected to drug-release analysis either in the absence or presence of esterase. Free ARV-771 was incubated as a control. All samples were loaded into dialysis tubes (MWCO 25 kDa) and incubated at 37 ℃ under gentle agitation. The cumulative appearance of ARV-771 in the dialysate was quantified by HPLC over 48 h (**Figure 2C**). Under sink conditions, free ARV-771 was rapidly released from the dialysis tube, achieving 92.9% release within 24 h. However, intact albumin-Alb-TAC conjugates exhibited a slow-release profile, with only 36.4% of ARV-771 released at 24 h, consistent with restricted diffusion due to the high molecular weight of the albumin-Alb-TAC conjugate. Notably, esterase-treated albumin-Alb-TAC conjugates showed a markedly accelerated release profile, with 90.4% of ARV-771 released within 24 h, closely resembling that of free ARV-771. These results indicate that albumin-Alb-TAC conjugates remain stable in the absence of esterase, thereby limiting premature, non-specific drug release in normal tissue. Concurrently, it enables on-demand release upon enzymatic activation, a unique PROTAC release mechanism expected to be advantageous for tumor-localized PROTAC activation *in vivo*. To further evaluate the long-term stability of Alb-TAC under physiological conditions, Alb-TAC was incubated in mouse serum over 96 h. DLS analysis confirmed that Alb-TAC maintained a constant hydrodynamic diameter of approximately 8-9 nm without significant aggregation or size changes throughout the incubation period (**Figure S11A**). LC-MS analysis further confirmed the chemical stability of Alb-TAC, with purity remaining consistent over 96 h. No evidence of premature ARV-771 release or linker degradation was observed (**Figure S11B**). These results demonstrate that Alb-TAC is resistant to nonspecific enzymatic degradation in systemic circulation, ensuring its long-term stability of in the bloodstream. To evaluate the tumor-selective activation potential of Alb-TAC, esterase concentration-dependent cleavage was assessed at three concentrations (3.75, 7.5, and 15.0 U/mL) by monitoring ARV-771 release over 6 and 12 h (**Figure S12**). At the lowest esterase concentration (3.75 U/mL), cleavage remained minimal (~10.5% at 6 h and ~13.2% at 12 h), indicating high stability under low-esterase conditions. In contrast, at 15.0 U/mL, cleavage increased substantially (~28.0% at 6 h and ~40.0% at 12 h), reflecting more efficient activation at elevated esterase levels. Although precise quantitative values of tumor esterase activity remain unreported in the literature, esterase activity in tumor tissues is generally recognized to be significantly higher than in normal tissues, suggesting that Alb-TAC is preferentially activated in the tumor microenvironment while remaining stable in systemic circulation 63, 64.

Given albumin's known role in enhancing tumor targeting through passive interstitial accumulation and active uptake by proliferating cancer cells, we next evaluated the intracellular behavior of Alb-TAC in CT26 mouse colon carcinoma cells. Cellular uptake was visualized using Cy5.5-conjugated ARV-771 and Cy5.5-labeled Alb-TAC. Confocal microscopy demonstrated efficient internalization of both samples over 24 h (**Figure 2D**). Quantitative fluorescence analysis revealed comparable uptake kinetics for Alb-TAC and ARV-771, with similar intracellular fluorescence intensities observed by 6 h and maintained up to 24 h (**Figure 2E**). These findings suggest that albumin conjugation of Alb-TAC in culture media does not significantly alter cellular uptake kinetics in the cell culture system. However, albumin conjugation primarily benefits *in vivo* delivery by enhancing tumor accumulation rather than altering cellular uptake kinetics directly. To further elucidate the intracellular trafficking pathway, co-localization analysis was performed using Cy5.5-labeled ARV-771 or Cy5.5-labeled Alb-TAC with LysoTracker^TM^ over 24 h. Confocal microscopy revealed that a substantial amount of Alb-TAC (red color) was sequestered within lysosomes (green color) from 6 to 24 h, whereas ARV-771(red color) was primarily distributed throughout the cytoplasm over the 24 h period (**Figure 2F**), suggesting that albumin conjugation directs Alb-TAC into the endo-lysosomal pathway 42, 65, 66. After 24 h post-incubation, the lysosomal co-localization of Cy5.5-labeled Alb-TAC was 7 times higher than that of Cy5.5-labeled ARV-771 (**Figure S13**). The lysosomal enrichment of Alb-TAC (yellow color; white arrows) is particularly significant, as the high esterase activity within lysosomes facilitates efficient linker cleavage and the subsequent release of active ARV-771 into the cytoplasm. To investigate whether intracellular esterase-mediated cleavage occurs following albumin-mediated endocytosis, CT26 cells were treated with Alb-TAC, and cell lysates were then collected at various time points (3, 6, 12, and 24 h) for LS-MS analysis (**Figure 2G**). Over time, the Alb-TAC peak diminished, while a new peak matching the retention time of native ARV-771 progressively emerged for 24 h. This conversion from Alb-TAC to ARV-771 confirms efficient intracellular cleavage and release of the active PROTAC in the cell culture system. The strongest ARV-771 signal (red-dotted circle) of Alb-TAC-treated CT26 cells was detected at 24 h, confirming sustained esterase-responsive PROTAC release of Alb-TAC in the cell culture system. LC-MS analysis of the 24 h peak revealed a major ion at *m/z* 1008 [M + Na]^+^, consistent with the molecular weight of ARV-771 (**Figure 2H**).

Finally, the unique function of Alb-TAC was further evaluated through cytotoxicity and protein degradation assays. Since BRD4 regulates the expression of oncogenes such as c-Myc and Bcl-2, its degradation is expected to suppress tumor cell proliferation 5-10. Concentration-dependent cytotoxicity assays over 48 h yielded half-maximal inhibitory concentrations (IC_50_) of 0.30 µM for Alb-TAC and 0.42 µM for ARV-771 (**Figure 2I**). These results suggest that Alb-TAC preserves and may enhance the cytotoxicity of ARV-771 in a cell culture system. To further characterize Alb-TAC-induced cell death, CT26 cells were treated with 5 µM Alb-TAC or 5 µM ARV-771, and the cell death was subsequently analyzed by Annexin V/PI staining after 48 h (**Figure 2J and S14**). Both treatments markedly decreased viable cell fractions (from 95.3% to 62.8% for ARV-771 and 63.1% for Alb-TAC). Early apoptosis (Annexin V+/PI-) increased to 2.71% (ARV-771) and 16.6% (Alb-TAC), while late apoptosis (Annexin V+/PI+) rose to 29.6% and 18.4%, respectively. Compared with ARV-771, Alb-TAC treatment resulted in a higher proportion of early apoptotic cells and a relatively lower proportion of late apoptotic cells. This pattern suggests that Alb-TAC efficiently induces substantial apoptosis but delays apoptosis progression. This is likely attributable to the delayed esterase-mediated cleavage of Alb-TAC prior to BRD4-degrading PROTAC release. A complementary live/dead viability assay also showed consistent increases in non-viable cells for both Alb-TAC and ARV-771 treatments (**Figure S15**). Collectively, these results confirmed that Alb-TAC efficiently conjugates to albumin, remains stable under physiological conditions, and undergoes esterase-responsive cell death in a cell culture system.

### Esterase-responsive BRD4 degradation and immunogenic cell death of Alb-TAC in a cell culture system

**Figure 3:**
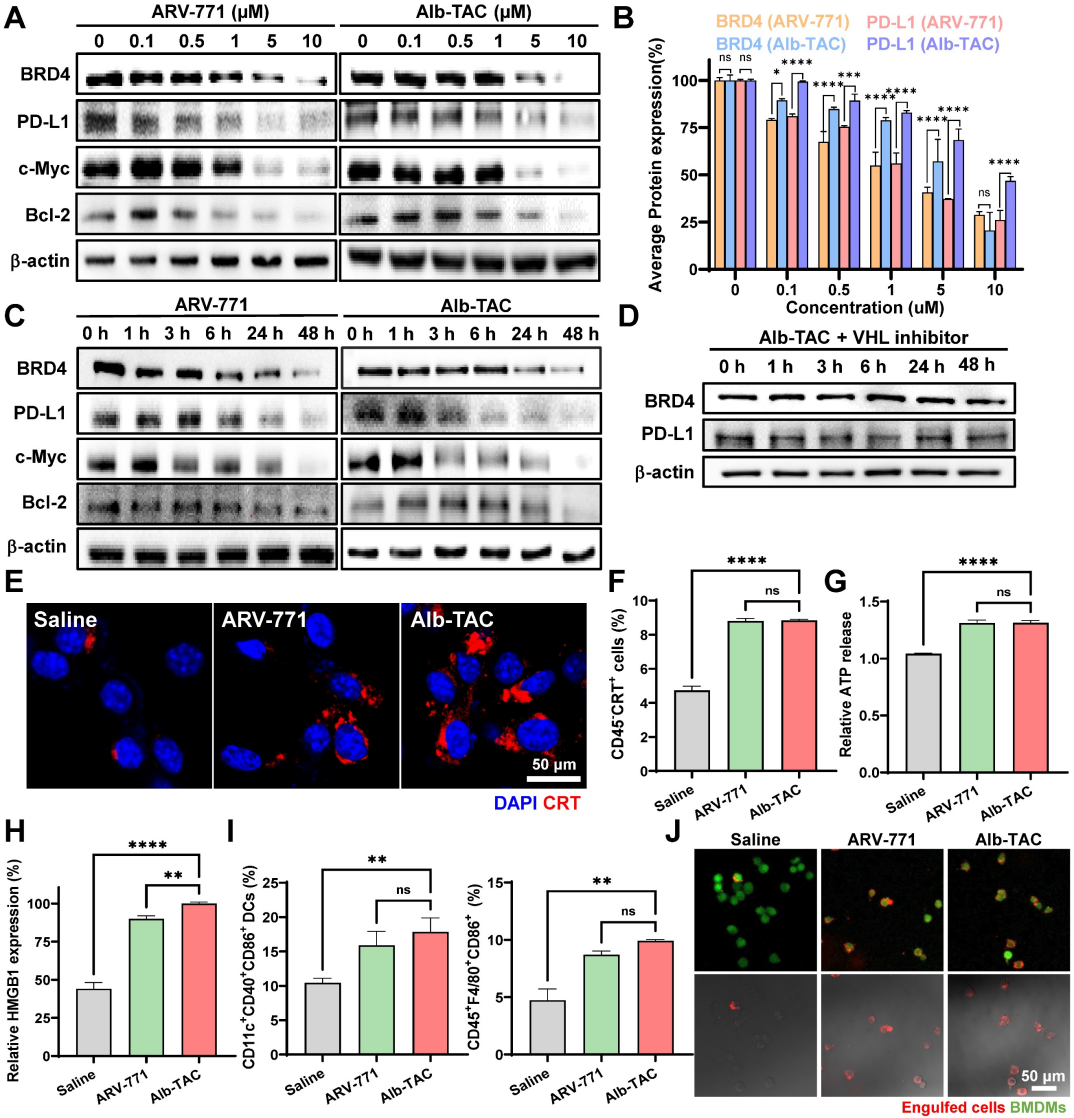
***In vitro* immunomodulatory characterization of Alb-TAC.** (A) Western blot analysis of CT26 cells treated with increasing concentrations (0, 0.1, 0.5, 1, 5, and 10 μM) of ARV-771 or Alb-TAC for 24 h to evaluate BRD4 degradation and changes in its downstream proteins (PD-L1, c-Myc, and Bcl-2). (B) Quantitative analysis of BRD4 and PD-L1 band intensities from (A). (C) Western blot analysis of CT26 cells treated with 10 μM ARV-771 or Alb-TAC for different incubation times (0, 1, 3, 6, 24, and 48 h) to assess time-dependent degradation kinetics of BRD4 and its downstream proteins (PD-L1, c-Myc, and Bcl-2). (D) Western blot analysis of CT26 cells co-treated with Alb-TAC (10 μM) and the VHL inhibitor VH298 to verify VHL-dependent BRD4 degradation over time. (E) CLSM images of CT26 cells treated with 10 μM ARV-771 or Alb-TAC for 24 h, stained with Alexa Fluor 647-conjugated anti-CRT antibody and DAPI to visualize cell-surface CRT exposure (scale bar, 50 μm). (F) Flow-cytometry quantification of surface CRT exposure in CT26 cells treated with 10 μM ARV-771 or Alb-TAC for 24 h (CD45^-^ CRT^+^). (G) Extracellular ATP levels in CT26 culture supernatants measured by a luminescence assay after 24 h treatment with 10 μM ARV-771 or Alb-TAC. (H) Western blot analysis of HMGB1 released into the culture supernatant from CT26 cells treated with 10 μM ARV-771 or Alb-TAC. (I) Flow-cytometric analysis of bone marrow-derived dendritic cells (BMDMs) and macrophages (BMDMs) co-cultured with CT26 cells pretreated with 10 μM ARV-771 or Alb-TAC for 24 h, showing mature DCs (CD11c^+^ CD40^+^ CD86^+^) and M1-like macrophages (CD45^+^ F4/80^+^ CD86^+^). (J) CLSM images of phagocytosis assays using BMDMs (green, CellTracker Green CMFDA) co-cultured with CT26 cells (red, pHrodo Deep Red) pretreated with 10 μM ARV-771 or Alb-TAC for 24 h. The upper panel shows fluorescent images (red and green channels), and the lower panel shows merged DIC/fluorescence images (scale bar, 50 μm). Data are presented as mean ± SD. Statistical significance was determined by two-way ANOVA with Tukey's post-hoc test for (B), and one-way ANOVA with Tukey's post-hoc test for (F-I) (ns, not significant; *p < 0.05; **p < 0.01; ***p < 0.001; ****p < 0.0001).

Western blot analysis was conducted to confirm the esterase-responsive targeted protein degradation of Alb-TAC within the cell culture system. To monitor protein degradation kinetics, CT26 cells were subjected to two regimens: increasing concentrations of ARV-771 (0 - 10 µM) or Alb-TAC (0 - 10 µM) for 24 h, or treatment with 10 µM of each compound and harvesting at different time points (0 - 48 h). In dose-dependent analysis, both ARV-771 and Alb-TAC induced substantial BRD4 degradation at higher concentrations (**Figure 3A**). At 10 µM, BRD4 protein levels were reduced by 71.3% upon ARV-771 treatment and by 79.5% following Alb-TAC treatment. In parallel, PD-L1 protein levels were reduced by 73.9% with ARV-771 and by 53.2% with Alb-TAC (**Figure 3B**). Consistent with BRD4 depletion, its downstream effectors, such as c-Myc, Bcl-2, and PD-L1, were markedly reduced. This is consistent with the important role of BRD4 in the transcriptional regulation of oncogenic targets, including c-Myc, Bcl-2, and PD-L1 5-10, 57. In addition, c-Myc and Bcl-2 exhibited marked reductions upon treatment with both compounds, with protein levels reduced by 60.2% and 71.0% for ARV-771 and by 77.9% and 84.1% for Alb-TAC, respectively. During dose-dependent degradation analysis, no hook effect was observed within the tested concentration range. The prodrug format of Alb-TAC enables sustained intracellular release of active ARV-771 following esterase-mediated cleavage, preventing abrupt increases in free PROTAC concentration and potential impairment of ternary complex formation 67. Time course analysis revealed progressive and substantial protein degradation following treatment with 10 µM of each compound (**Figure 3C and Figure S16**). At 48 h, BRD4 protein levels were reduced by 80.5% upon ARV-771 treatment and by 63.4% following Alb-TAC treatment. In parallel, PD-L1 expression showed pronounced suppression over time, with protein levels reduced by 76.3% for ARV-771 and by 83.3% for Alb-TAC at 48 h. In addition, c-Myc protein levels were robustly decreased, with reductions of 90.3% upon ARV-771 treatment and 85.4% following Alb-TAC treatment. Bcl-2 expression was similarly suppressed, decreasing by 73.1% with ARV-771 and by 65.7% with Alb-TAC at 48 h. Although BRD4 and c-Myc suppression was slightly less pronounced with Alb-TAC than with ARV-771, PD-L1 downregulation was comparable or greater, potentially reflecting the role of BRD4 as a critical transcriptional regulator of CD274 57. Comparable dose- and time-dependent BRD4 degradation was also observed in 4T1 breast cancer cells, demonstrating that the esterase-responsive degradation mechanism extends across distinct solid tumor models (**Figure S17**). These results demonstrate that both ARV-771 and Alb-TAC effectively induce BRD4 degradation in a dose- and time-dependent manner, accompanied by the coordinated suppression of key BRD4-regulated oncogenic proteins, including PD-L1, c-Myc, and Bcl-2. Notably, the comparable degradation kinetics of Alb-TAC and free ARV-771 suggest that the intracellular activation step of the Alb-TAC does not impair the efficiency of the downstream degradation cascade, confirming that ternary complex formation is highly efficient even after drug release on demand. To further verify that this degradation proceeds through the classical ubiquitin-proteasome system (UPS), each step of the PROTAC mechanism was systematically interrogated using specific inhibitors. The PROTAC mechanism involves sequential VHL E3 ligase recruitment, ubiquitination of the target protein, and proteasomal degradation. CT26 cells were co-treated with the VHL inhibitor VH298 and analyzed in a time-dependent manner (**Figure 3D and Figure S18**). In the presence of VH298, Alb-TAC did not induce BRD4 degradation across all examined time points, indicating effective disruption of PROTAC-mediated E3 ligase recruitment. Quantitative band analysis revealed that BRD4 protein levels remained largely unchanged throughout the time course, with variations within 4% compared to 0 h. Consistently, PD-L1 expression also remained stable over time, exhibiting deviations within 6%, indicating that Alb-TAC-mediated PD-L1 downregulation is dependent on VHL-driven BRD4 depletion. To further confirm ubiquitination- and proteasome-dependent degradation, CT26 cells were co-treated with Alb-TAC and either a ubiquitination inhibitor (Pevonedistat, a NAE inhibitor that blocks cullin-dependent ubiquitination) or a proteasome inhibitor (Bortezomib) in a time-dependent manner up to 48 h (**Figure S19**). In both cases, BRD4 and PD-L1 levels were maintained near baseline throughout the incubation period. Inhibition of each step effectively abolished Alb-TAC-mediated BRD4 degradation, demonstrating that Alb-TAC functions through the complete UPS pathway, with each step being strictly dependent on VHL recruitment, ubiquitination, and proteasomal activity.

Given the observed suppression of PD-L1, the immunogenic cell death (ICD) potential of Alb-TAC was subsequently evaluated. ICD is a regulated form of cell death characterized by the release or exposure of DAMPs, including HMGB1, ATP, and CRT. These DAMPs perform distinct and complementary roles in immune activation, in which CRT serves as an 'eat-me' signal for dendritic cell-mediated phagocytosis, ATP acts as a 'find-me' signal to recruit immune cells, and HMGB1 promotes DC maturation *via* TLR4 signaling 68, 69. Their coordinated induction stimulates dendritic cells and promotes adaptive antitumor immunity through cumulative complementary signals rather than any single mediator. To assess whether Alb-TAC induces ICD, hallmark DAMP markers were examined in CT26 cells. Surface exposure of CRT was first evaluated in CT26 cells treated with 10 µM ARV-771 or Alb-TAC for 24 h using CLSM and flow cytometry. CLSM images revealed increased CRT localization on the cell surface following both treatments, with a visibly stronger signal in the Alb-TAC-treated group (**Figure 3E**). Consistently, flow cytometric analysis demonstrated a significant increase in CRT-positive cell populations, reaching approximately 1.18-fold relative to saline-treated controls, which confirmed effective induction of ICD-associated CRT exposure (**Figure 3F**). Additional ICD hallmarks were further quantified. Extracellular ATP levels, measured by a luminescence-based assay, were elevated by 1.27-fold following treatment with either ARV-771 or Alb-TAC compared with saline-treated controls (**Figure 3G**). In contrast, HMGB1 release into the extracellular milieu, assessed by immunoblotting of culture supernatants, exhibited a marked compound-dependent difference. Alb-TAC treatment resulted in a 2.26-fold increase in extracellular HMGB1 relative to saline-treated control cells and a 1.11-fold increase compared with ARV-771, thus indicating more robust induction of ICD by Alb-TAC (**Figure 3H and Figure S20**). Together, these results demonstrate that Alb-TAC robustly induces multiple hallmark features of immunogenic cell death, including CRT exposure, ATP secretion, and HMGB1 release, thereby providing a mechanistic basis for subsequent immune cell activation. Next, to determine whether Alb-TAC-induced ICD translated into innate immune activation, bone marrow-derived dendritic cells (BMDCs) and bone marrow-derived macrophages (BMDMs) from BALB/c mice were co-cultured with CT26 cells previously treated with 10 µM ARV-771 or 10 µM Alb-TAC at 37 ℃ for 24 h. To quantify the effect of Alb-TAC-treated cancer cells on DC maturation and macrophage M1 polarization, FACS analysis was performed. Flow cytometric analysis revealed that Alb-TAC-treated tumor cells significantly enhanced immune cell activation (**Figure 3I**). The proportion of mature DCs (CD11c^+^CD40^+^CD86^+^) increased by 1.71-fold relative to saline-treated control and by 1.12-fold compared with ARV-771. Similarly, M1-like macrophages (CD45^+^F4/80^+^CD86^+^) were elevated by 2.09-fold relative to saline-treated control and by 1.14-fold relative to ARV-771, thus demonstrating a stronger innate immune-activating effect of Alb-TAC. To further visualize the functional consequence of ICD-induced immune priming, a phagocytosis assay was performed using BMDMs (green) co-cultured with CT26 cells (red) pretreated with 10 µM ARV-771 or 10 µM Alb-TAC at 37 ℃ for 24 h. Confocal microscopy revealed pronounced phagocytosis of CT26 cells by BMDMs, with enhanced phagocytosis observed in the Alb-TAC group (**Figure 3J**). These findings provide direct evidence that Alb-TAC-induced ICD promotes macrophage-mediated phagocytosis of tumor cells, thereby complementing the observed increases in DC maturation and M1 polarization. Together, these results demonstrate that Alb-TAC not only degrades BRD4 and suppresses oncogenic signaling but also effectively engages the BRD4-DR5 mechanistic axis to elicit immunogenic cell death. By inducing this immunogenic mode of death, Alb-TAC primes antigen-presenting cells toward a pro-inflammatory phenotype, thereby transforming the tumor microenvironment into an 'immunologically hot' state.

### *In vivo* tumor targeting ability of Alb-TAC in CT26 tumor-bearing tumor models

**Figure 4:**
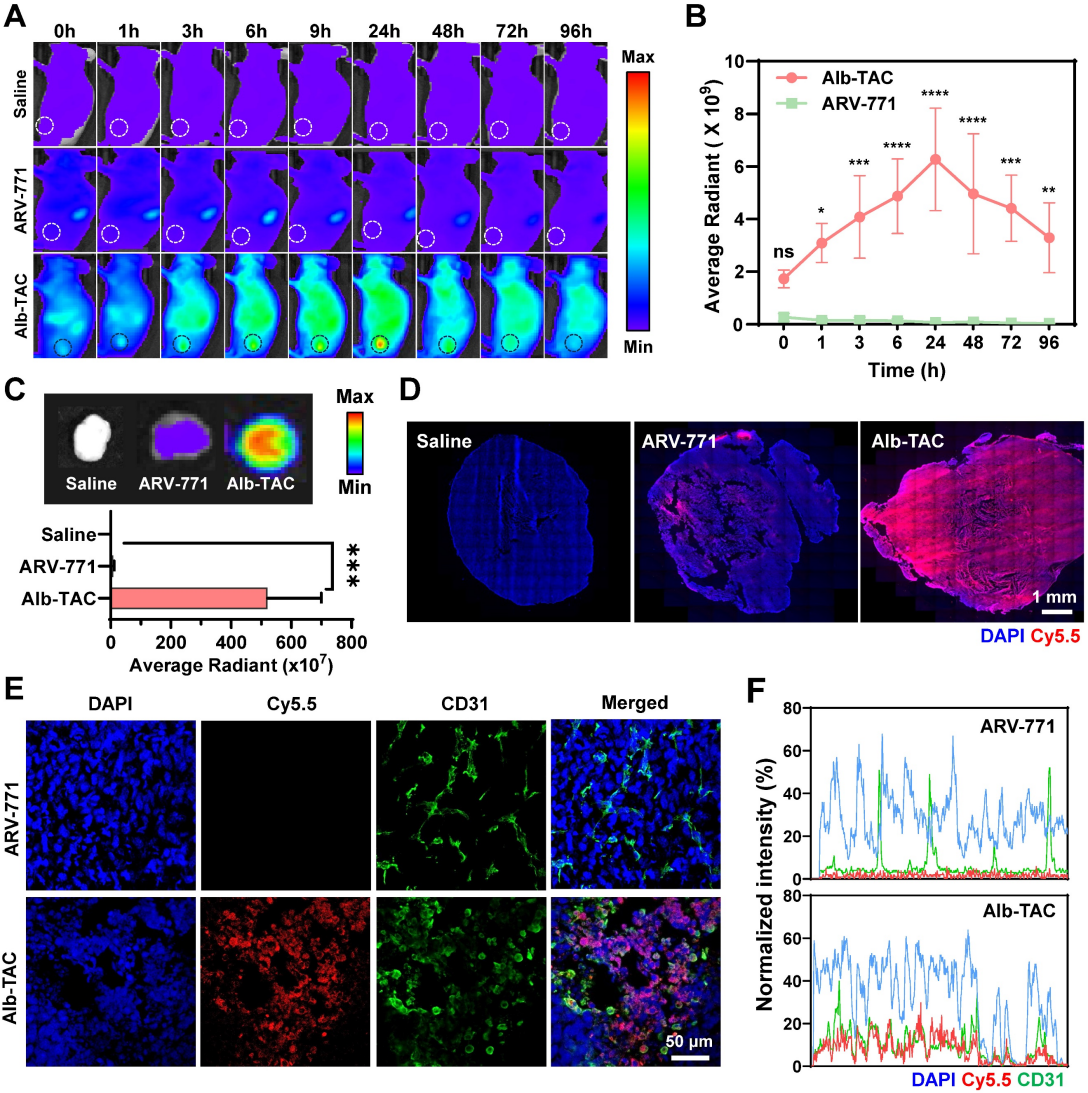
***In vivo* pharmacokinetics of Alb-TAC.** (A) Non-invasive NIRF images of CT26 tumor-bearing mice (n = 3 per group) intravenously injected with Cy5.5-labeled Alb-TAC (3 mg/kg, equiv. to Alb-TAC) or Cy5.5-labeled ARV-771 (3 mg/kg, equiv. to ARV-771) at different time points. Dotted circles indicate the tumor region. (B) Quantitative analysis of fluorescence intensity in the tumor region obtained from (A). (C) *Ex vivo* fluorescence images of excised tumors collected 24 h post-injection and corresponding quantitative analysis of fluorescence intensity. (D) Representative fluorescence images of whole-tumor tissues collected 24 h post-injection showing intratumoral distribution of Cy5.5-labeled ARV-771 and Alb-TAC (scale bar, 1 mm). (E) CLSM images of tumor section co-stained with DAPI (nuclei) and CD31 (endothelial marker) (scale bar, 50 μm). (F) Cross-section fluorescence intensity profiles corresponding to (E). Data are presented as mean ± SD. Statistical significance was determined by two-way ANOVA with Tukey's post-hoc test for (B) (ns, not significant; *p < 0.05; **p < 0.01; ***p < 0.001; ****p < 0.0001).

The *in vivo* biodistribution of Alb-TAC was carefully evaluated in CT26 tumor-bearing BALB/c mice and directly compared with that of ARV-771. To assess the tumor targeting ability of Alb-TAC, CT26 tumor-bearing mice with tumor volumes of approximately 150-200 mm^3^ were intravenously administered Cy5.5-labeled Alb-TAC or Cy5.5-labeled ARV-771 at an equivalent dose (3 mg/kg for each compound, n = 3). Time-dependent tumor targeting efficiency of Alb-TAC was monitored using non-invasive NIRF imaging over a 96h period (**Figure 4A**). Following intravenous administration, Cy5.5-labeled Alb-TAC exhibited sustained systemic fluorescence signals over time, indicating prolonged blood circulation. In contrast, Cy5.5-labeled ARV-771 showed a rapid signal decay consistent with fast systemic clearance. Notably, Cy5.5-labeled Alb-TAC progressively accumulated at the tumor site, reaching maximal fluorescence intensity at 24 h post-injection. Quantitative analysis of tumor-associated fluorescence revealed that Alb-TAC displayed an 87.4-fold higher fluorescence intensity at 24 h compared with ARV-771 (**Figure 4B**), demonstrating markedly enhanced tumor accumulation. In contrast, Cy5.5-labeled ARV-771 exhibited weak fluorescence with minimal retention in major organs (except for the kidneys), consistent with rapid renal clearance. This observation aligns with the known pharmacokinetic limitation of ARV-771, which undergoes rapid elimination due to its poor stability under physiological conditions 14. Collectively, these results indicate that the albumin binding mechanism of Alb-TAC extends the systemic circulation and enhances the tumor targeting ability *in vivo*.

*Ex vivo* fluorescence imaging was performed at 24 h post-injection using an additional cohort of CT26 tumor-bearing mice to further corroborate the *in vivo* biodistribution findings. For this analysis, mice (n = 3) were independently administered either Cy5.5-labeled Alb-TAC or Cy5.5-labeled ARV-771. Subsequently, after 24 h post-injection, tumors and major organs (liver, lung, spleen, kidney, and heart) were harvested for *ex vivo* imaging. Excised tumors from Cy5.5-labeled Alb-TAC-treated mice exhibited significantly stronger fluorescence signals than those from Cy5.5-labeled ARV-771-treated mice (**Figure 4C and Figure S21**). Quantitative analysis revealed that tumor fluorescence intensity in the Alb-TAC group was 59.8-fold higher than that of the ARV-771-treated group, confirming superior tumor-specific delivery mediated by albumin binding. To assess intratumoral penetration, tumor tissues excised at 24 h post-injection were immediately cryosectioned and analyzed by confocal microscopy. Fluorescence imaging of the cryosectioned tumor tissues revealed that Cy5.5-labeled Alb-TAC was broadly distributed throughout the tumor parenchyma, extending well beyond perivascular regions. In contrast, Cy5.5-labeled ARV-771 exhibited sparse signals that were largely restricted to areas proximal to tumor blood vessels (**Figure 4D**). To further delineate the spatial relationship between drug distribution and tumor vasculature, the same cryosectioned tumor tissues were subsequently subjected to immunofluorescent staining with an anti-CD31-FITC antibody to label endothelial cells, alongside DAPI counterstaining to visualize nuclei. Co-localization analysis confirmed that Cy5.5-labeled Alb-TAC efficiently diffused beyond CD31-positive vascular regions into the tumor interstitium. In contrast, Cy5.5-labeled ARV-771 fluorescence remained predominantly confined to areas near CD31-positive vasculature (**Figure 4E**). Cross-sectional fluorescence intensity profiling further confirmed that Cy5.5-labeled Alb-TAC efficiently diffused beyond CD31-positive regions, demonstrating enhanced interstitial distribution and tissue penetration (**Figure 4F**). Together, these results demonstrate that Alb-TAC exhibits superior pharmacokinetic behavior, enhanced tumor accumulation, and improved intratumoral penetration compared with ARV-771, validating *in situ* albumin binding as an effective strategy for improving PROTAC delivery to solid tumors *in vivo*.

### *In vivo* therapeutic efficiency and immune remodeling of Alb-TAC in CT26 tumor-bearing mice

**Figure 5:**
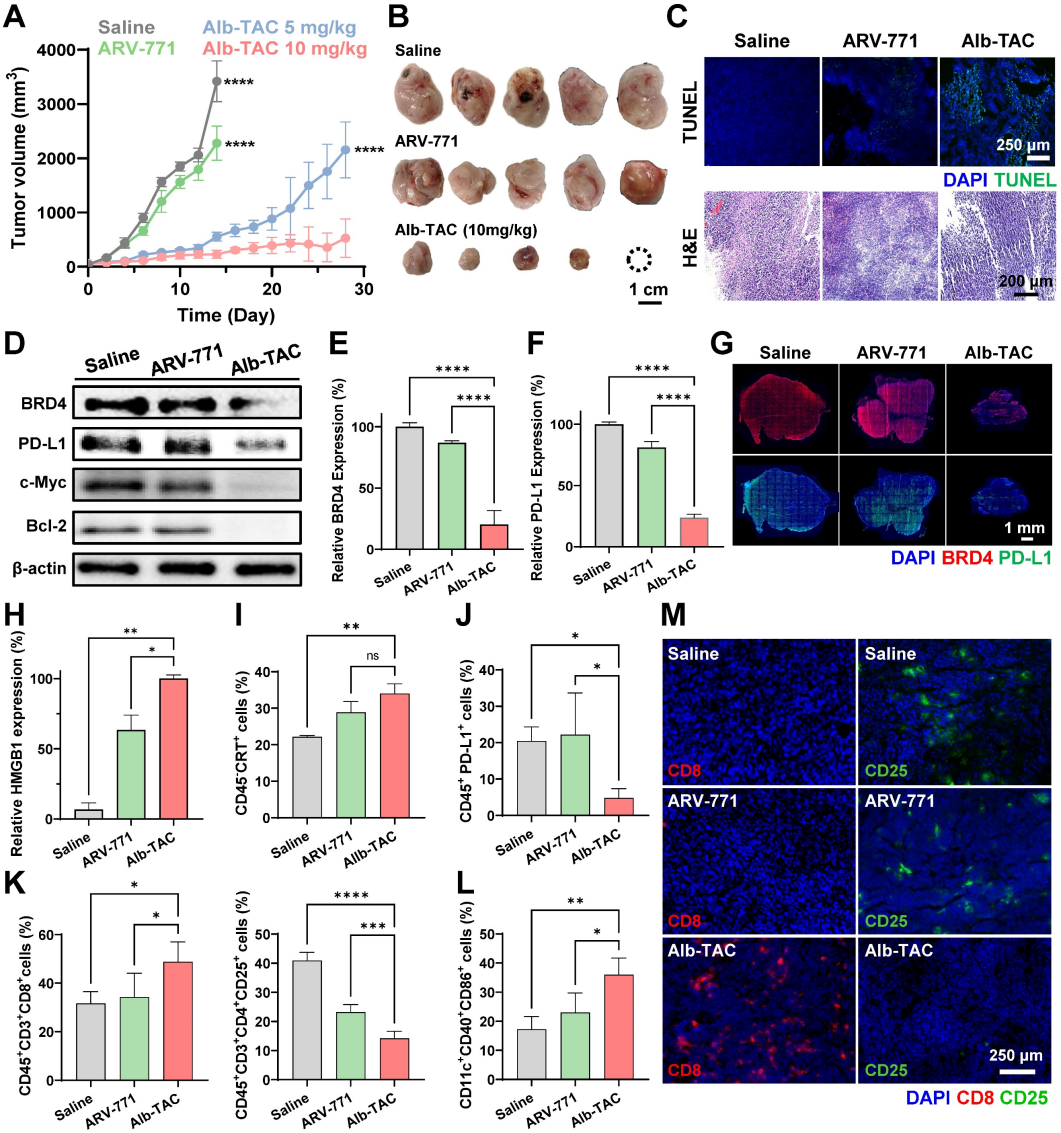
***In vivo* therapeutic efficacy and antitumor immunity of Alb-TAC.** (A) Tumor growth curves of CT26 tumor-bearing mice treated intravenously with saline, ARV-771 (10 mg/kg), Alb-TAC (5 mg/kg), or Alb-TAC (10 mg/kg, equiv. to ARV-771) every three days for four doses (days 0, 3, 6, and 9). (B) Representative photographs of excised tumors collected on day 15 from each treatment group (n=5). (C) Terminal deoxynucleotidyl transferase dUTP nick and labeling (TUNEL; top) staining and hematoxylin and eosin (H&E; bottom) of tumor sections on day 15 (scale bars, 250 μm for TUNEL and 200 μm for H&E). (D) Western blot analysis of tumor lysates from each group showing expression of BRD4 and its downstream proteins (PD-L1, c-Myc, and Bcl-2). (E, F) Quantitative analysis of BRD4 and PD-L1 band intensities from (D). (G) Immunofluorescence staining of tumor sections for BRD4 and PD-L1 (scale bar, 1 mm). (H) Western blot analysis of tumor lysates collected 7 days after treatment showing the expression of high-mobility group box 1 (HMGB1). (I) Flow-cytometry quantification of surface calreticulin (CRT) expression on tumor cells (CD45^-^ CRT^+^). (J) Flow-cytometric analysis of PD-L1-positive immune cells (CD45^+^ PD-L1^+^) within tumor cells. (K) Flow-cytometric analysis of cytotoxic T lymphocytes (CD45^+^ CD3^+^ CD8^+^) infiltrating tumor tissues (left) and Tregs (CD45^+^ CD3^+^ CD4^+^ CD25^+^) isolated from tumors (right). (L) Flow-cytometric analysis of mature dendritic cells (DCs; CD11c^+^ CD40^+^ CD86^+^) within tumors. (M) Immunofluorescence staining of tumor sections co-stained with CD8 (red) and CD25 (green) to visualize intratumoral infiltration of cytotoxic T cells and regulatory T cells, respectively (scale bar, 50 μm). Data are presented as mean ± SD. Statistical significance was determined by two-way ANOVA with Tukey's post-hoc test for (A), and one-way ANOVA with Tukey's post-hoc test for (E, F, H-L) (ns, not significant; *p < 0.05; **p < 0.01; ***p < 0.001; ****p < 0.0001).

Following the confirmation of Alb-TAC's improved pharmacokinetic properties and tumor-targeting ability, its *in vivo* therapeutic efficacy and antitumor immunity were evaluated in CT26 tumor-bearing BALB/c mice. When tumor volumes reached approximately 80 ± 20 mm^3^, mice were randomized into saline (n = 5), ARV-771 (10 mg/kg; n = 5), and Alb-TAC (10 mg/kg, equiv. to ARV-771; n = 5) treatment groups. These groups were used to assess early antitumor response, and mice were euthanized on day 15 for *ex vivo* analysis. An additional cohort, comprising Alb-TAC (5 mg/kg; n = 5) and Alb-TAC (10 mg/kg; n = 5), was included to assess dose-dependent therapeutic efficiency and extended therapeutic durability up to day 28. All treatments were administered intravenously every three days, and tumor volumes and body weight were monitored once every two days. Quantitative tumor volume analysis revealed pronounced and dose-dependent antitumor efficacy of Alb-TAC relative to both saline and ARV-771 controls (**Figure 5A**). Specifically, on day 14, the average tumor volumes in the saline and ARV-771 groups reached 3418.15 ± 376.15 mm^3^ and 2277.47 ± 316.86 mm^3^, respectively. In contrast, Alb-TAC treatment significantly restricted tumor growth, resulting in volumes of 552.23 ± 83.39 mm^3^ (5 mg/kg) and 229.97 ± 75.53 mm^3^ (10 mg/kg). Alb-TAC 5 mg/kg also produced substantial tumor inhibition, yielding tumors 6.48-fold smaller than saline and 4.32-fold smaller than ARV-771. Extended monitoring of the Alb-TAC cohorts revealed sustained antitumor effects even after treatment cessation. By day 28, tumors in the Alb-TAC 10 mg/kg group remained significantly suppressed, with mean volumes of 526.81 ± 353.07 mm^3^. These were 3.90-fold smaller than those observed in the Alb-TAC 5 mg/kg group (2156.57 ± 515.49 mm^3^), indicating durable therapeutic control at the higher dose. Consistent with quantitative tumor volume measurements, excised tumors collected on day 15 confirmed reduced mass in Alb-TAC-treated mice, with some individuals exhibiting near-complete regression (**Figure 5B**). To further assess the generalizability of these findings beyond the CT26 model, Alb-TAC (10 mg/kg) was evaluated in the 4T1 breast tumor model, where it demonstrated marked tumor growth suppression through day 14. The mean tumor volume in the Alb-TAC group (163.30 ± 115.24 mm^3^) was substantially lower than that of both the saline (838.53 ± 39.24 mm^3^) and ARV-771 (763.49 ± 170.90 mm^3^) groups (**Figure S22**). Collectively, these results demonstrate that Alb-TAC achieves superior *in vivo* antitumor efficacy and durable tumor suppression compared with ARV-771. This enhanced therapeutic performance is consistent with prolonged systemic exposure and efficient tumor accumulation mediated by the *in situ* albumin binding mechanism, rather than from increased intrinsic PROTAC activity alone. Following tumor volume measurement, tumors excised on day 15 were next subjected to histological and molecular analyses to assess treatment-induced cellular responses. Consistent with the pronounced tumor growth inhibition, histological analyses revealed enhanced apoptotic activity in Alb-TAC-treated tumors. TUNEL staining revealed widespread apoptosis in Alb-TAC-treated tumors, evidenced by intense and diffuse TUNEL-positive nuclei. In contrast, ARV-771 and saline groups showed minimal apoptotic signals (**Figure 5C, top**). These results indicate that Alb-TAC elicits a robust apoptotic response *in vivo*, aligning with its enhanced therapeutic efficacy. Hematoxylin and eosin (H&E) staining further corroborated these findings. Tumors from the Alb-TAC group displayed extensive cellular disruption and markedly reduced tumor cell density, in contrast to the dense, viable architecture observed in the ARV-771 and saline groups (**Figure 5C, bottom**). To confirm molecular mechanisms underlying this enhanced antitumor activity, Western blot analysis was conducted on tumor lysates. Alb-TAC treatment resulted in significant downregulation of BRD4 and its downstream oncogenic effectors, including PD-L1, c-Myc, and Bcl-2 (**Figure 5D**). Densitometric quantification showed that BRD4 levels decreased 4.93- and 4.29-fold relative to saline and ARV-771, respectively, while PD-L1 levels decreased 4.22- and 3.42-fold relative to saline and ARV-771, respectively (**Figure 5E and 5F**). These molecular changes are consistent with the enhanced apoptosis and tumor regression observed in Alb-TAC-treated mice. Immunofluorescence staining further corroborated these molecular findings, revealing pronounced reductions in BRD4 and PD-L1 expression in Alb-TAC-treated tumors (**Figure 5G**). In contrast, ARV-771 elicited minimal changes. These observations, combined with the apoptotic and histological analyses, suggested that Alb-TAC not only suppresses oncogenic signaling but may also modulate the TME.

The immunosuppressive TME represents a major barrier to effective cancer therapy. Hence, therapeutics capable of inducing tumor cell death while simultaneously enhancing antitumor immunity are of significant clinical interest. Given that Alb-TAC induced immunogenic cell death (ICD) and DAMP release *in vitro*, its ability to remodel the TME *in vivo* was further investigated. For this purpose, immune profiling of tumor-infiltrating leukocytes was performed by multicolor flow cytometry using single-cell suspensions isolated seven days after treatment initiation. Western blot and quantitative analysis demonstrated substantial upregulation of HMGB1, a canonical DAMP associated with ICD-driven immune activation. HMGB1 levels increased 15.11-fold relative to saline and 1.58-fold relative to ARV-771 (**Figure 5H and Figure S23**). Surface CRT on tumor cells (CD45^-^CRT^+^) was similarly elevated, increasing 1.54-fold over saline and 1.18-fold over ARV-771. This further confirmed the immunogenic nature of Alb-TAC-induced tumor cell death (**Figure 5I**). In parallel, flow cytometric analysis of tumor-infiltrating immune subsets revealed that Alb-TAC significantly reduced the proportion of PD-L1-positive immune cells within the tumor (**Figure 5J**). The detailed gating strategy used to identify these immune and tumor cell population is provided in **Figure S24-S27**. The frequency of CD45^+^PD-L1^+^ cells decreased to roughly one-third of that in saline-treated tumors and was markedly lower than in ARV-771-treated tumors. This indicates suppression of PD-L1 expression in tumor-associated immune cells and alleviation of immunosuppressive signaling within the TME. Moreover, Alb-TAC promoted a substantial increase in cytotoxic CD8^+^ T cells (CD45⁺CD3⁺CD8⁺), with 1.54-fold and 1.42-fold enhancements relative to saline and ARV-771, respectively (**Figure 5K**). These increased CD8^+^ T cells indicate enhanced infiltration and activation of effector T cells. Conversely, the population of regulatory T cells (Tregs; CD45⁺CD3⁺CD4⁺CD25⁺) decreased 2.89-fold compared to saline and 1.64-fold compared to ARV-771, thus shifting the immune balance toward a more immunostimulatory phenotype. Alb-TAC treatment also enhanced dendritic cell (DC) maturation, as reflected by increased expression of the co-stimulatory marker CD86 (CD11c⁺CD40⁺CD86⁺), which was 2.08-fold above saline and 1.56-fold above ARV-771 (**Figure 5L**). To visualize intratumoral immune remodeling, immunofluorescence staining of tumor sections was performed (**Figure 5M**). Consistent with flow cytometry, Alb-TAC-treated tumors displayed a markedly higher density of infiltrating CD8^+^ T cells (red) and a pronounced reduction in CD25^+^ Tregs, with CD8^+^ T cells uniformly dispersed throughout the tumor parenchyma. These spatial patterns reinforce the enhanced effector T cell recruitment and reduced immunosuppressive signaling induced by Alb-TAC. To further elucidate the functional status of the TME, we quantified the expression of key cytokines profiles in the plasma of CT26 tumor-bearing mice (**Figure S28**). Alb-TAC treatment induced significant changes in the intratumoral cytokine profile. The expression of TGF-β, a potent immunosuppressive cytokine, was reduced by 0.55-fold compared to the saline group and by 0.57-fold compared to the ARV-771 group, consistent with the observed decrease in Treg infiltration. Notably, IFN-γ levels, a hallmark of active Th1-type antitumor immunity, were significantly increased by 5.16-fold compared to the saline group and by 4.40-fold compared to the ARV-771 group. Interestingly, IL-6 levels also increased by approximately 1.55-fold compared to the saline and ARV-771 groups. Given the simultaneous increase in IFN-γ and the robust inductin of ICD, the elevation of IL-6 reflects a heightened inflammatory state within the TME, which further promotes the recruitment and activation of immune cells. These functional cytokine data, coupled with the flow cytometric results, provide comprehensive evidence that Alb-TAC effectively remodels the TME from an immunosuppressive cold state to a pro-inflammatory hot environment. Together, these cytometric and histological findings demonstrate that Alb-TAC acts not only as a potent BRD4 degrader and tumor-killing agent but also as an effective ICD inducer, actively reshaping the TME. Alb-TAC promotes DAMP release, reduces PD-L1-mediated immune suppression, enhances CD8^+^ T cell infiltration, suppresses Treg prevalence, and stimulates DC maturation. These actions collectively drive the TME toward a pro-inflammatory, immunostimulatory state supportive of durable antitumor immunity.

### Systemic toxicity of Alb-TAC in CT-26 tumor-bearing mice

**Figure 6:**
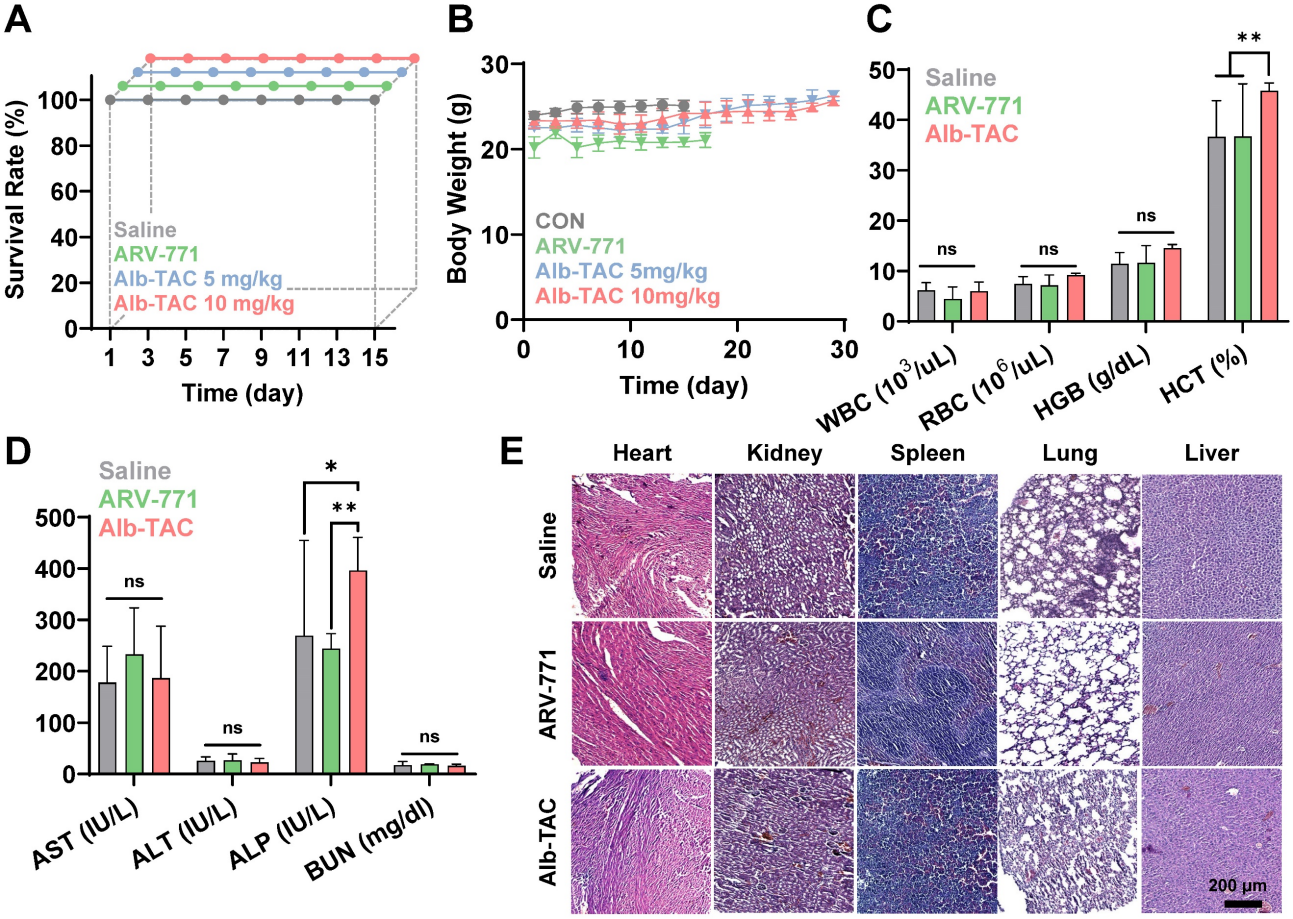
***In vivo* safety profile of Alb-TAC.** (A,B) Survivor rate (A) and body weight (B) changes of mice during the treatment and follow-up periods. (C) Hematological analysis of total white blood cells (WBC), red blood cells (RBC), hemoglobin (HGB), and hematocrit (HCT) after the final dose. (D) Serum biochemical analysis of hepatic (AST, ALT, ALP) and renal (BUN) function markers in each treatment group. (E) Histological examination (H&E staining) of major organs (liver, lung, spleen, kidney, and heart) collected from mice after treatment (scale bar, 100 μm). Data are presented as mean ± SD. Statistical significance was determined by one-way ANOVA with Tukey's post-hoc test for (C, D) (ns, not significant; *p < 0.05; **p < 0.01).

To assess the *in vivo* safety profile of Alb-TAC, systemic toxicity was monitored throughout the treatment period. Survival rate and body weight were first evaluated as general indicators of treatment tolerance. No treatment-relative mortality was observed in any group during the 15-day observation period. All mice treated with ARV-771 (10 mg/kg), Alb-TAC (5 mg/kg), or Alb-TAC (10 mg/kg) survived throughout the study, comparable to the saline-treated group (**Figure 6A**). In addition, no significant body weight loss or abnormal behavior was detected in any treatment group during the treatment period (days 0-15) (**Figure 6B**). Notably, Alb-TAC-treated mice, including both the 5 mg/kg and 10 mg/kg dose groups, maintained stable body weight without signs of systemic distress during extended monitoring up to day 28. This indicated good tolerability even at the higher dose and after repeated administration. Consistent with these observations, no significant body weight loss was observed in Alb-TAC-treated mice (10 mg/kg) in the 4T1 model throughout the 14-day monitoring period, further supporting its tolerability across distinct solid tumor settings (**Figure S29**). To further investigate potential systemic toxicity, comprehensive hematological and serum biochemical analyses were performed on mice treated with Alb-TAC at the highest dose (10 mg/kg) at the study endpoint (day 15) following repeated intravenous administration. As shown in **Figure 6C**, no significant alterations were observed in key hematological parameters, including total white blood cell count (WBC), red blood cell count (RBC), hemoglobin (HGB), and hematocrit (HCT). This confirmed that Alb-TAC administration did not induce hematologic toxicity. Similarly, serum biochemical profiles revealed that the levels of hepatic function markers (AST, ALT, and ALP) and the renal function marker (BUN) in the Alb-TAC (10 mg/kg)-treated group remained within normal physiological ranges and were comparable to those in the control group (**Figure 6D**). Finally, histological examination of major organs (liver, lung, spleen, kidney, and heart) *via* H&E staining was conducted to detect any tissue-level damage. Consistent with the biochemical findings, no noticeable tissue damage, apoptosis, or inflammation was detected in any treatment group (**Figure 6E**). Notably, despite previous fluorescence imaging suggesting hepatic exposure, the absence of liver toxicity implies selective pharmacological activity in tumors. Collectively, these findings demonstrate that Alb-TAC exhibits a favorable systemic safety profile with minimal off-target toxicity.

## Conclusion

While PROTACs have demonstrated significant therapeutic potential, their clinical translation has been hindered by rapid systemic clearance, suboptimal pharmacokinetic stability, and insufficient tumor selectivity, challenges particularly pronounced in solid tumors. To overcome these limitations, we rationally designed, synthesized, and systematically compared two albumin-binding BRD4-degrading PROTAC candidates (Alb-TAC#1 and Alb-TAC#2). Each candidate incorporated a Cys34-reactive maleimide moiety for *in situ* albumin conjugation and an esterase-cleavable linker for tumor-localized activation. Through comparative evaluation, systematic structure-function analysis identified Alb-TAC#2 as an optimized lead candidate, featuring a sterically minimized and spatially accessible maleimide-linker architecture. It demonstrated that Alb-TAC#2 showed the superior albumin-binding stability, accelerated esterase-mediated cleavage, and enhanced tumor accumulation compared to both Alb-TAC#1 and ARV-771. By leveraging endogenous albumin as a physiological carrier, Alb-TAC#2 achieved prolonged systemic exposure and efficient tumor localization *via* EPR- and GP60/SPARC-mediated transport pathways. Once internalized into tumor cells, esterase-responsive cleavage of Alb-TAC#2 facilitated the rapid release of the active PROTAC. This Alb-TAC#2 elicited potent BRD4 degradation and suppression of downstream oncogenic factors, including c-Myc, Bcl-2, and PD-L1. Beyond direct oncogenic suppression, Alb-TAC#2 also initiated a robust immunomodulatory cascade by triggering immunogenic cell death (ICD). This was characterized by extracellular ATP release, surface CRT exposure, and HMGB1 translocation. These DAMP-mediated signals facilitated dendritic cell maturation and antigen presentation, leading to enhanced infiltration of cytotoxic CD8^+^ T cells and reduced regulatory T cells within the tumor. Together with PD-L1 downregulation, these coordinated changes reshaped the tumor immune microenvironment toward a more immunostimulatory state, capable of supporting durable antitumor responses. Collectively, Alb-TAC#2 represents a next-generation immune-PROTAC strategy that integrates pharmacokinetic optimization, tumor-selective activation, targeted protein degradation, and immunological engagement. Its chemically programmable design, which includes albumin-binding motifs, esterase-cleavable linkers, and PROTAC warheads, provides a generalizable framework for engineering degraders with improved systemic stability, selective activation, and immunological synergy. This modular architecture is potentially extensible to other PROTAC architectures containing accessible hydroxyl groups, such as reported EGFR- and CDK4/6-targeting degraders 65, 70. These findings expand the translational potential of TPD-based therapeutics, especially for solid tumors, and underscore albumin-binding immune-PROTACs as a promising avenue for future clinical advancement.

## Supplementary Material

Supplementary figures.

## Data Availability

The data that support the findings of this study are available from the corresponding author upon reasonable request.

## Abbreviations

ACN: Acetonitrile
AE-Mal: N-(2-aminoethyl)maleimide
Alb-TACs: albumin-binding BRD4-degrading PROTACs
ALP: Alkaline phosphatase
ALT: Alanine aminotransferase
AST: Aspartate aminotransferase
ATP: adenosine triphosphate
BCL2: B-cell lymphoma 2
BCN-PEG2-Mal: bicyclononyne-polyethylene glycol-maleimide
BET: bromodomain and extra-terminal
BMDCs: bone marrow-derived dendritic cells
BMDMs: bone marrow-derived macrophages
BRD4: bromodomain-containing protein 4
BSA: bovine serum albumin
BUN: blood urea nitrogen
CCK-8: Cell Counting Kit-8
CLSM: confocal laser scanning microscopy
CRT: calreticulin
Cy5.5: Cyanine 5.5
DAMPs: damage-associated molecular patterns
DAPI: 4',6-diamidino-2-phenylindole
DCM: dichloromethane
DCs: dendritic cells
DIPEA: N,N-diisopropylethylamine
DLS: dynamic light scattering
DMAP: 4-dimethylaminopyridine
DMF: N,N-dimethylformamide
DMSO: dimethyl sulfoxide
EDC-HCl: N-(3-dimethylaminopropyl)-N'-ethylcarbodiimide hydrochloride
EPR: enhanced permeation and retention
FBS: fetal bovine serum
H&E: Hematoxylin and eosin
HATU: 1-[Bis(dimethylamino)methylene]-1H-1,2,3-triazolo[4,5-b]pyridinium 3-oxide hexafluorophosphate
HCT: hematocrit
HGB: hemoglobin
HMGB1: high-mobility group box 1
HPLC: high-performance liquid chromatography
IACUC: Institutional Animal Care and Use Committee
ICD: immunogenic cell death
LC-MS: liquid chromatography-mass spectrometry
MALDI-TOF: Matrix-assisted laser desorption/ionization-time of flight
MWCO: molecular weight cut-off
MYC: MYC proto-oncogene, bHLH transcription factor
NIRF: near-infrared fluorescence
PAGE: polyacrylamide gel electrophoresis
PBS: phosphate-buffered saline
PD-L1: Programmed death-ligand 1
PROTACs: Proteolysis-targeting chimeras
RBC: red blood cell count
RP-HPLC: Reverse-phase high-performance liquid chromatography
RPMI: Roswell Park Memorial Institute
RT: room temperature
SPF: specific-pathogen-free
TEA: triethylamine
TEM: transmission electron microscopy
TME: tumor microenvironment
TPD: Targeted protein degradation
Tregs: regulatory T cells
TUNEL: terminal deoxynucleotidyl transferase dUTP nick and labeling
UPS: ubiquitin-proteasome system
VHL: Von Hippel-Lindau
WBC: white blood cell count
