# Supplementary material for: Esterase-responsive albumin-binding PROTAC-mediated BRD4 degradation for cancer immunotherapy
Source: Theranostics. 2026 Apr 23;16(11):6240–65. doi: 10.7150/thno.130510 (PMC13142674; doi:10.7150/thno.130510)
Supplement: Supplementary file 1 — Supplementary figures. [file thnov16p6240s1.pdf]

Supporting Informations for Theranostics

Esterase-responsive albumin-binding PROTAC-mediated BRD4 degradation for cancer immunotherapy

Hoyeon Lee[1,2,#], Sojin Jeong[1,#], Juwon Park[1,#], Hye-jeong Son[3], Seho Kweon[3], Steve Seung-Young Lee[4], Nayeon Shim[1], Yoojeong Oh[1], Hyein Kang[1,2], Chaerin Lee[1,2], Jihyeon Lee[1,2], Jinseong Kim[1], Hanhee Cho[1,5,*], Kwangmeyung Kim[1,2,*]

[1]College of Pharmacy, Graduate School of Pharmaceutical Sciences, Ewha Womans University, Seoul, 03760, Republic of Korea.

[2]Graduate Program in Innovative Biomaterials Convergence, Ewha Womans University, Seoul 03760, Korea.

[3]College of Pharmacy, Chonnam National University, Gwangju 61186, Republic of Korea.

[4]Department of Pharmaceutical Sciences, University of Illinois Chicago, Chicago, IL, USA

[5]Noxpharm Co., LTD., Seoul 03759, Republic of Korea

[#]These authors contributed equally to this work.

*Correspondence and requests for materials should be addressed to **H. Cho** (E-mail: hanheecho31@gmail.com) and **K. Kim** (E-mail: kimkm@ewha.ac.kr)

Supporting Figures

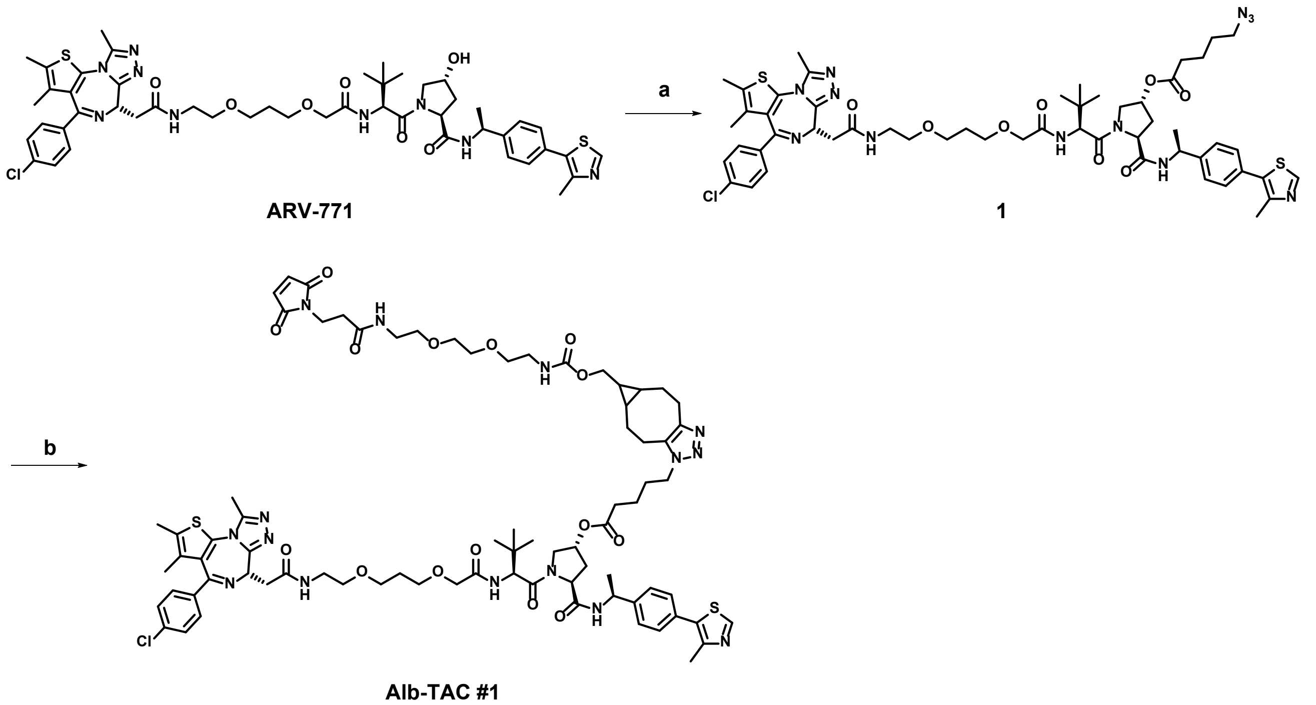

Figure S1. Synthetic scheme of Alb-TAC #1. Schematic representation of the multi-step synthesis of the esterase-responsive, albumin-binding BRD4 PROTAC (Alb-TAC #1). Reagents and conditions: (a) 5-azidovaleric acid, EDC, DMAP, CH_2Cl_2, RT, 1 h; (b) BCN-PEG_2-Mal, DMF, RT, 1 h.

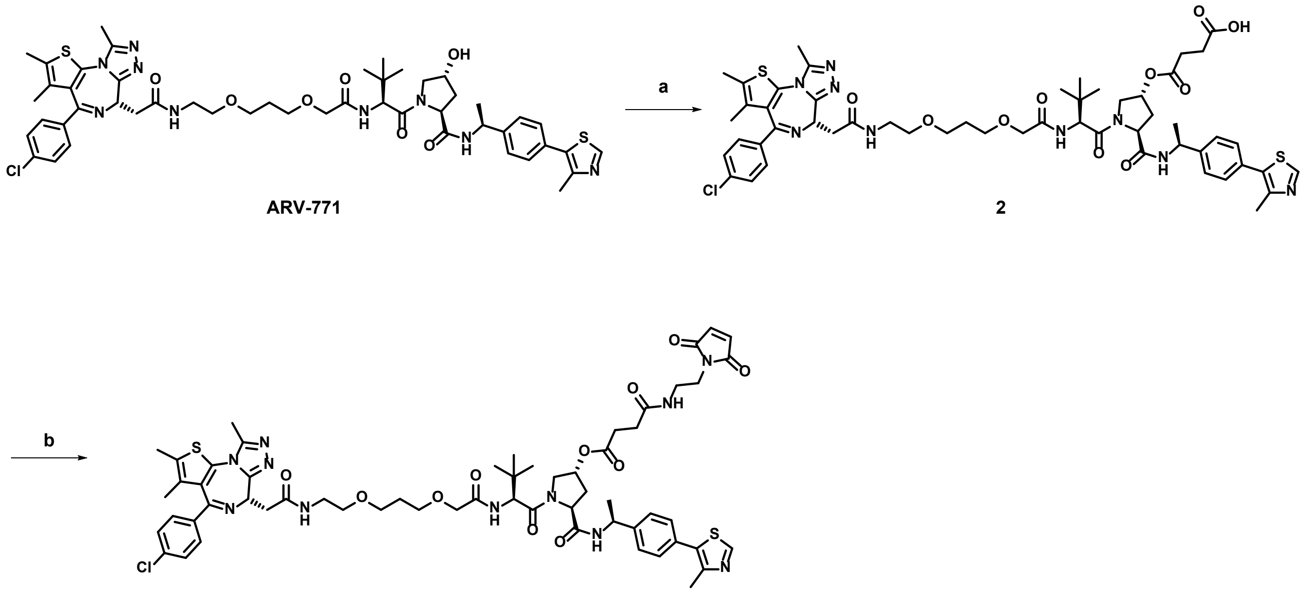

ARV-771 → 2 → Alb-TAC #2

Figure S2. Synthetic scheme of Alb-TAC #2. Schematic representation of the multi-step synthesis of the Alb-TAC #2. Reagents and conditions: (a) succinic acid, EDC, DMAP, CH_2Cl_2, THF, RT, 1 h; (b) N-(2-aminoethyl)maleimide, HATU, DIPEA, DMF, RT, 1 h.

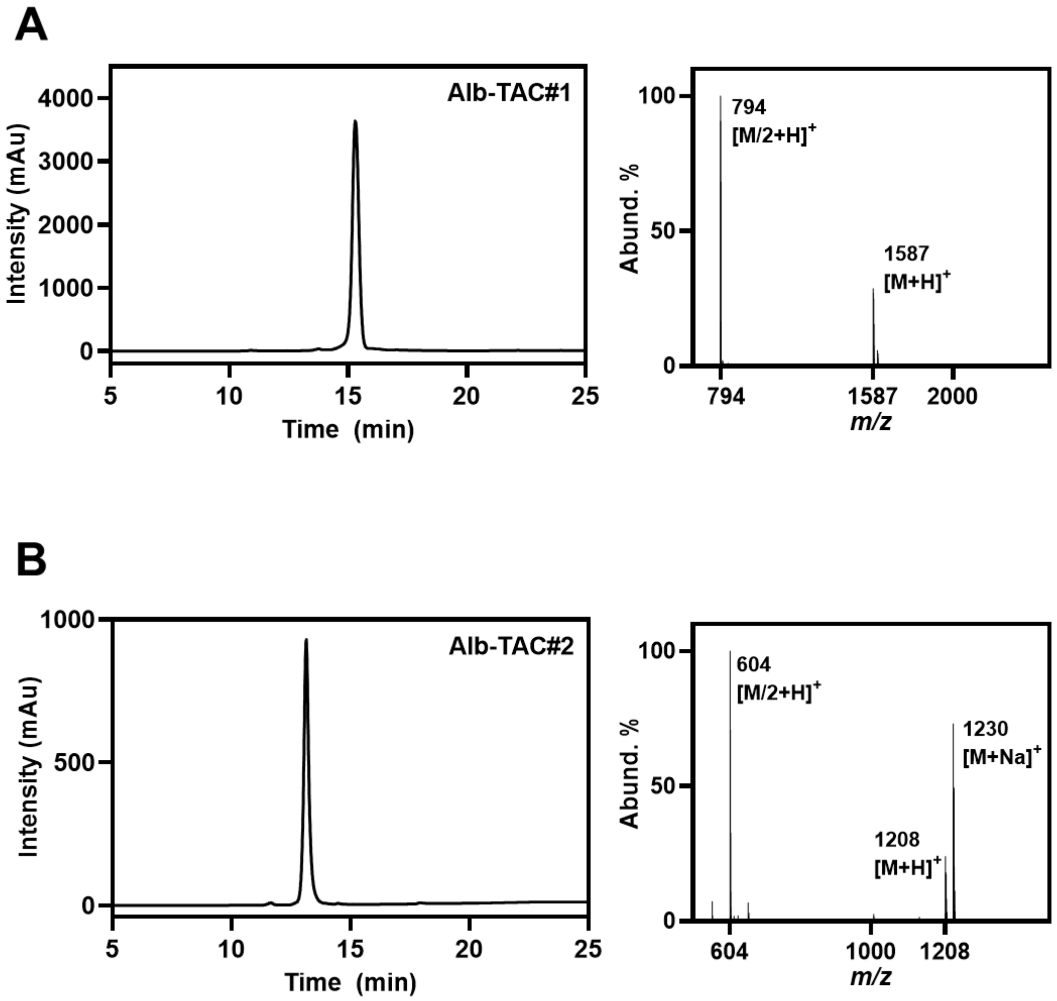

Figure S3. Characterization of Alb-TACs. (A) Analytical HPLC chromatogram and LC-MS spectra of (a) Alb-TAC#1 (*m/z* 1587 $[M + H]^{+}$ and 794 $[M+2H]^{2+}$) and (B) Alb-TAC#2 (*m/z* 1208 $[M + H]^{+}$, 1230 $[M + Na]^{+}$ and 604 $[M+2H]^{2+}$) . The presence of a single sharp peak and the observed mass confirm the high purity and identity of the synthesized conjugates.

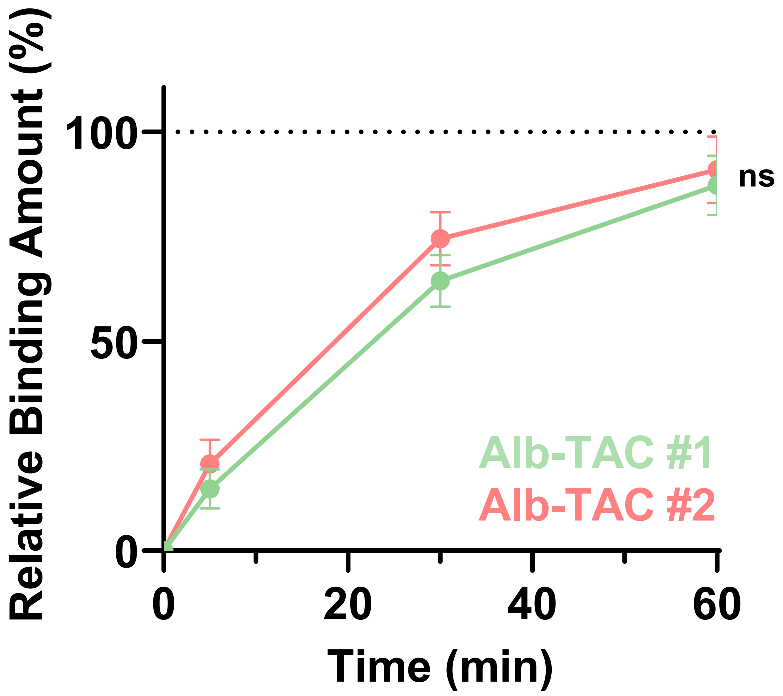

Figure S4. Quantitative evaluation of albumin-binding kinetics. Time-dependent relative binding amounts of Alb-TAC#1 and Alb-TAC#2. Each Alb-TAC (70 μM) was incubated with serum albumin (100 μM) in saline at 37 °C with constant stirring. The binding percentage was quantified based on the decrease in free Alb-TACs peak areas observed *via* HPLC. Data are presented as mean ± SD. Statistical significance was determined by Student's t-test (ns, no significant).

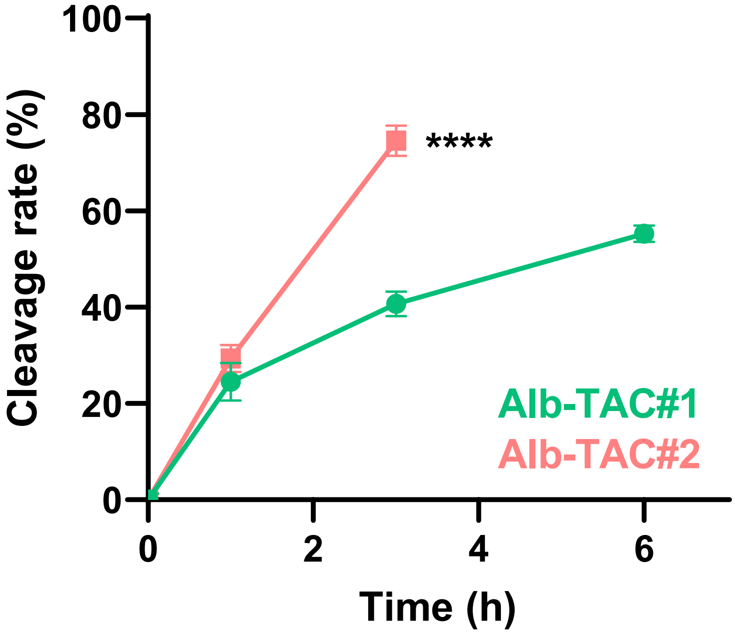

Figure S5. Quantitative cumulative hydrolysis profiles of Alb-TACs. Quantitative cumulative hydrolysis profiles of Alb-TAC#1 and Alb-TAC#2 over 12 h of incubation with esterase (30 U/mL) at 37 °C. The percentage of hydrolysis was calculated by quantifying the peak area of the released ARV-771 relative to the initial Alb-TAC peak using RP-HPLC. Data are presented as mean ± SD. Statistical significance was determined by Student's t-test (****p < 0.0001).

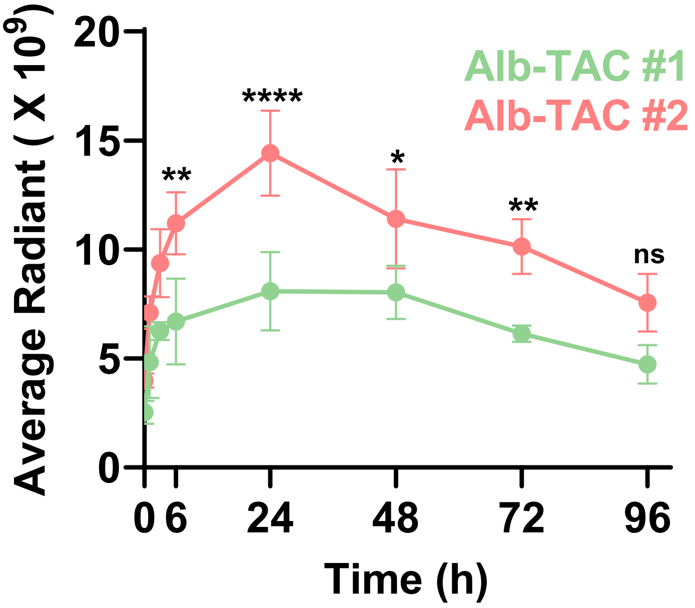

Figure S6. Quantitative analysis of *in vivo* biodistribution of Alb-TAC #1 and Alb-TAC #2. Time-dependent fluorescence intensity profiles of tumor regions obtained from NIRF imaging (Figure 1G) following intravenous administration of Cy5.5-labeled Alb-TAC #1 or Alb-TAC #2 (10 mg/kg) in CT26 tumor-bearing mice (n = 3 per group). Fluorescence intensities were quantified from tumor-localized regions of interest (ROIs) at each time point. Data are presented as mean ± SD. Statistical significance was determined by two-way ANOVA with Tukey's post-hoc test for (B) (ns, not significant; *$p < 0.05$; **$p < 0.01$; ****$p < 0.0001$).

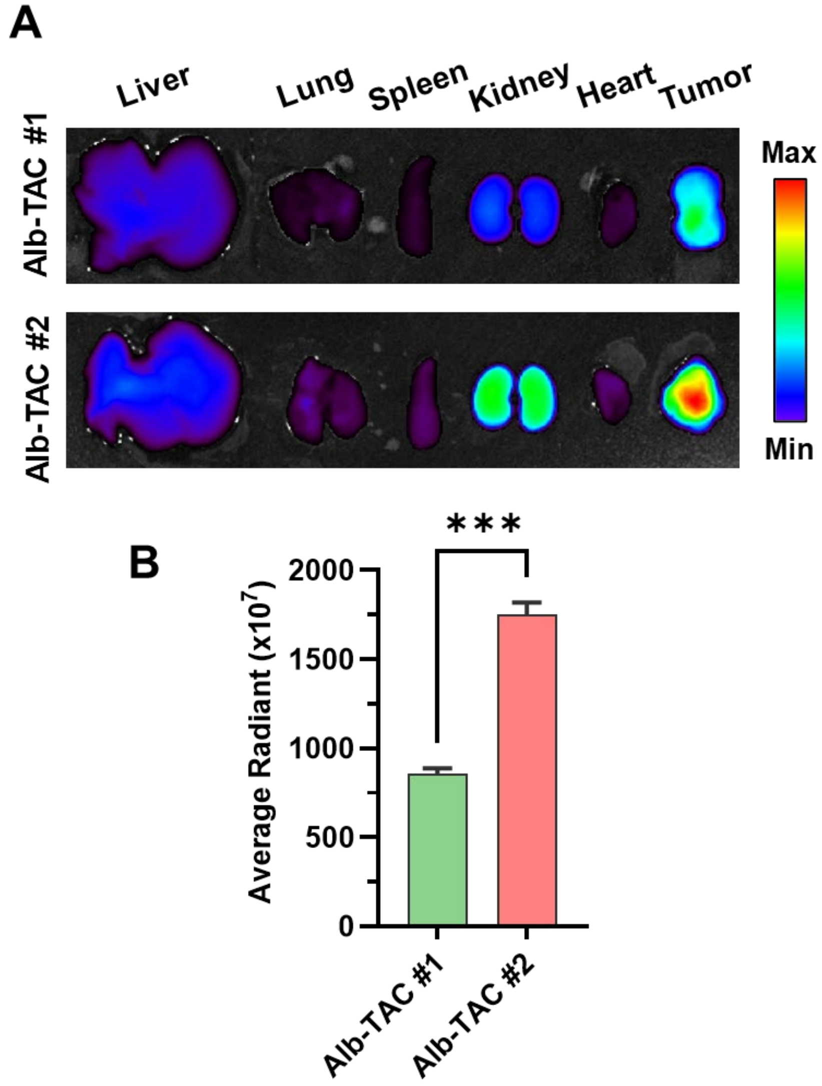

Figure S7. ***Ex vivo*** **biodistribution analysis of Cy5.5-labeled Alb-TAC #1 and Alb-TAC #2.** (A) Fluorescence images of major organs (liver, lung, spleen, kidney, and heart) and tumor tissues collected 24 h after intravenous injection of Cy5.5-labeled Alb-TAC #1 or Alb-TAC #2 (10 mg/kg) in CT26 tumor-bearing mice (n = 3 per group). (B) Quantification of tumor-associated fluorescence signals shown in (A), derived from *ex vivo* imaging of excised tumors collected 24 h post-injection. Data are presented as mean ± SD. Statistical significance was determined by Student's t-test (***$p <$ 0.001).

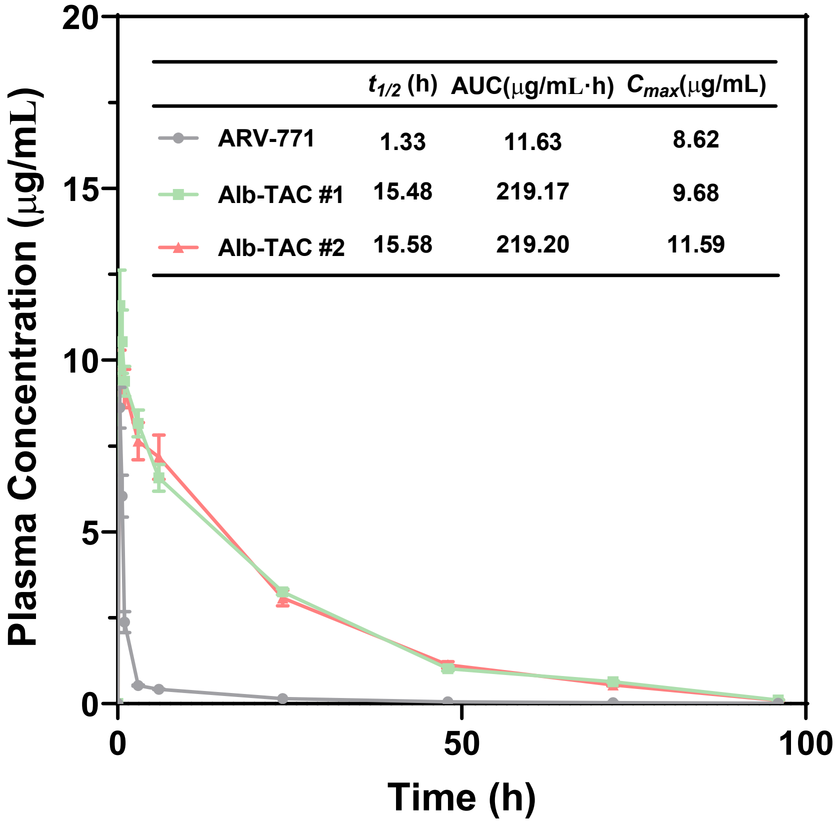

Figure S8. ***In vivo*** **pharmacokinetic profiles of Alb-TACs in normal mice.** Plasma concentration-time curves of Cy5.5-conjugated ARV-771 (10 mg/kg, equiv. to ARV-771), Cy5.5-conjugated Alb-TAC#1 (10 mg/kg, equiv. to ARV-771), and Cy5.5-conjugated Alb-TAC#2 (10 mg/kg, equiv. to ARV-771) following a single intravenous injection in BALB/c mice. The inserted table summarizing key pharmacokinetic parameters, including half-life ($t_{1/2}$), area under the curve (AUC), and maximum plasma concentration (C_{max}). Data represent mean ± SD (n = 3).

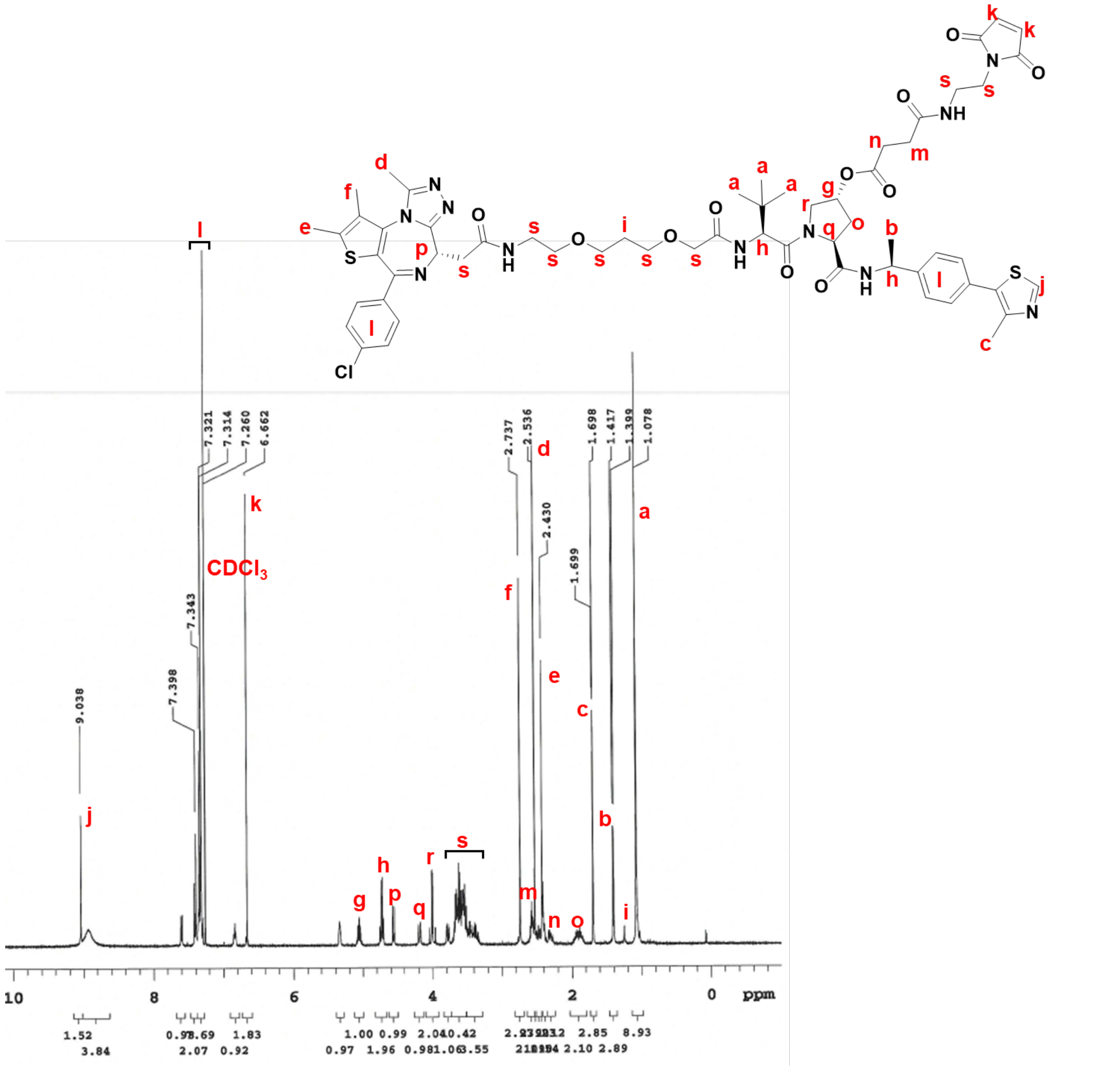

Figure S9. ^{1}H NMR spectrum of Alb-TAC#2. ^{1}H NMR (600 MHz) spectrum of Alb-TAC#2 measured in $CDCl_3$ (10 mg/mL). The chemical structure is labeled with letters corresponding to the assigned peaks in the spectrum.

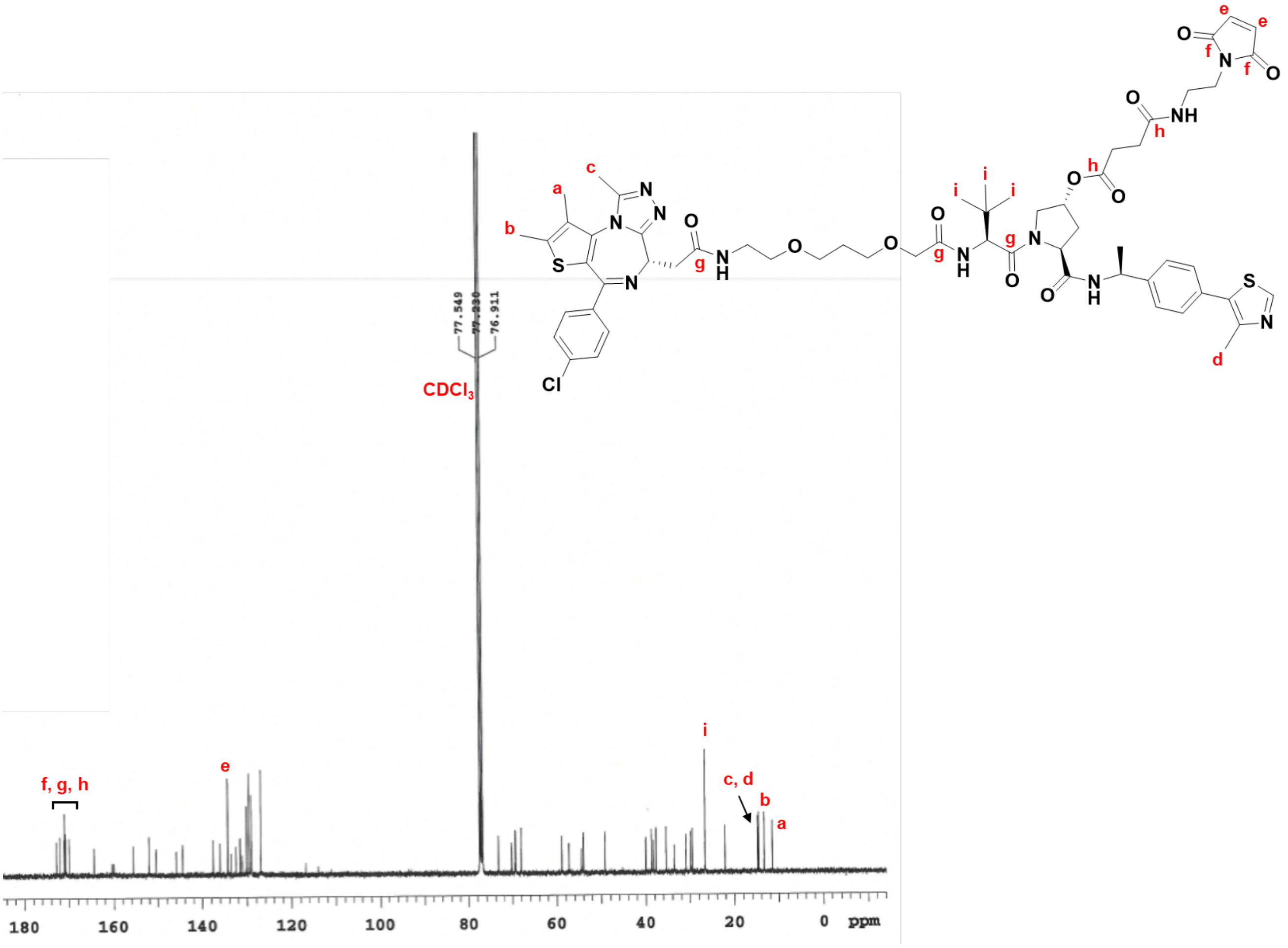

Figure S10. ^{13}C NMR spectrum of Alb-TAC#2. ^{13}C NMR (600 MHz) spectrum of Alb-TAC#2 measured in $CDCl_3$ (10 mg/mL). The chemical structure is labeled with letters corresponding to the assigned peaks in the spectrum.

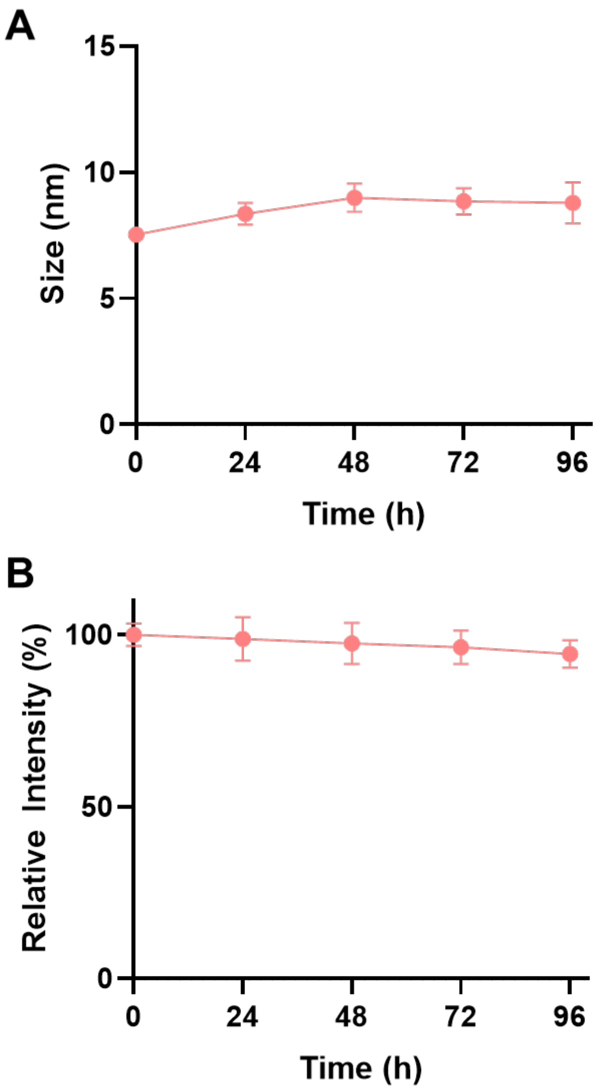

Figure S11. Long-term stability of Alb-TAC in mouse serum. (A) Hydrodynamic size distribution of Alb-TAC measured by DLS during incubation in mouse serum at 37 °C for 96 h. (B) LC-MS analysis of Alb-TAC purity, expressed as the percentage of Alb-TAC peak area relative to total peak area, in mouse serum at 37 °C over 96 h.

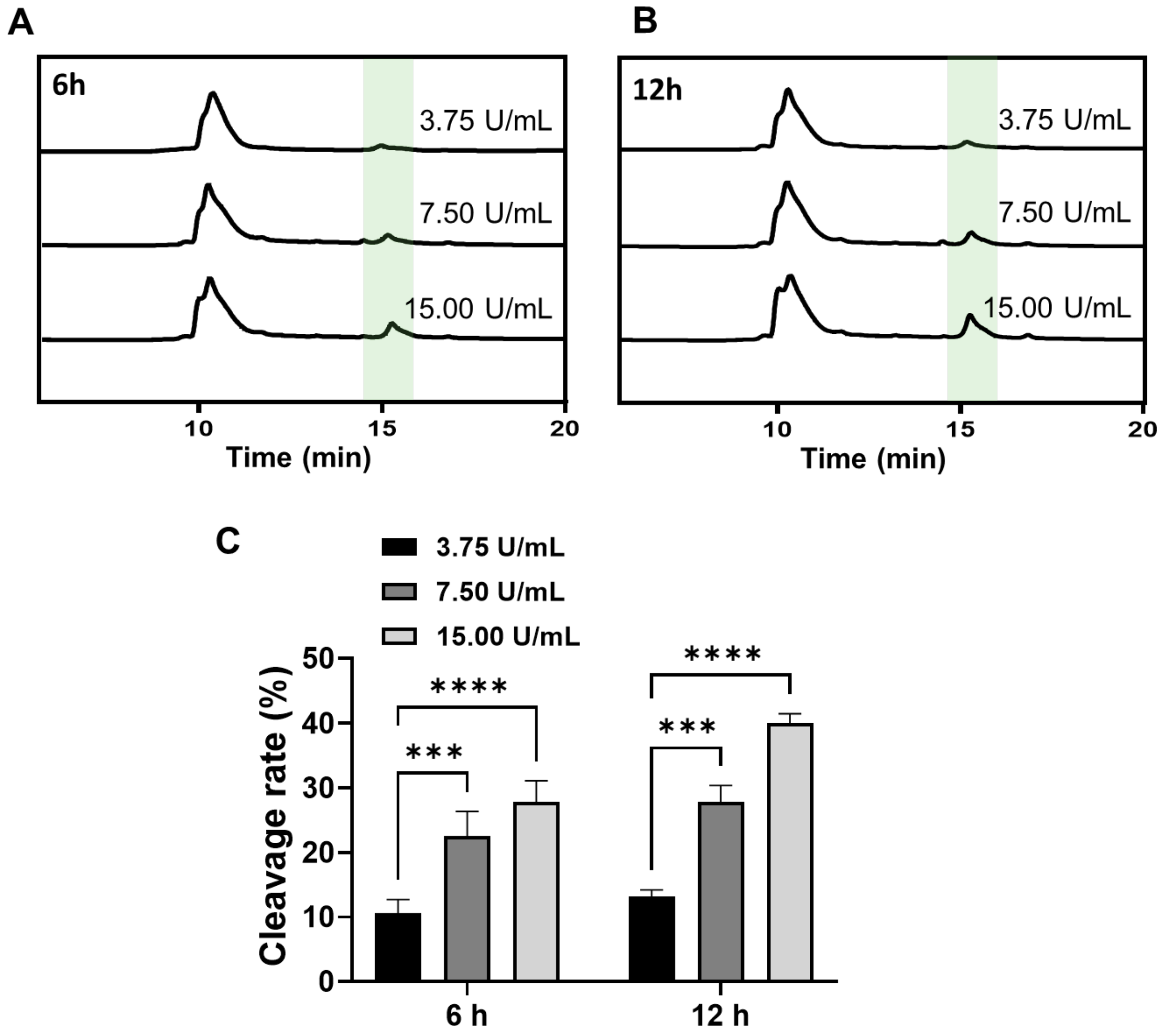

Figure S12. Esterase concentration-dependent cleavage and release kinetics of Alb-TAC. (A, B) RP-HPLC chromatograms of Alb-TAC following incubation with varying concentrations of esterase (3.75, 7.50, and 15.00 U/mL) for 6 h and 12 h. The shaded green area indicates the peak corresponding to released ARV-771. (C) Quantitative analysis of Alb-TAC cleavage rate (%) as a function of esterase concentration and incubation time, calculated as the peak area of released ARV-771 relative to total peak area. Data are presented as mean ± SD. Statistical significance was determined by two-way ANOVA with Tukey's post-hoc test for (C) (***$p < 0.001$; ****$p < 0.0001$).

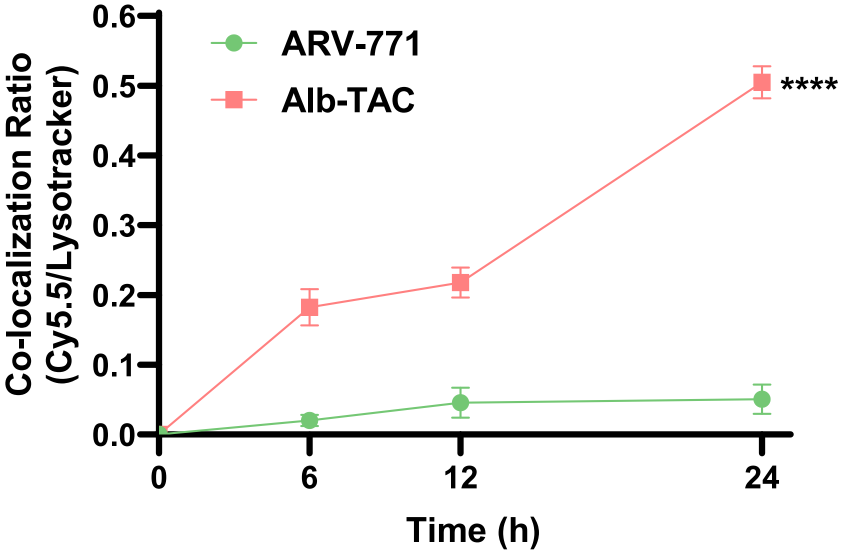

Figure S13. Quantitative co-localization analysis of Alb-TAC with LysoTracker. Quantitative co-localization analysis of Cy5.5-labeled ARV-771 and Cy5.5-labeled Alb-TAC with LysoTracker in CT26 cells, expressed as co-localization ratio over 24 h. Data are presented as mean ± SD. Statistical significance was determined by Student's t-test (****p < 0.0001).

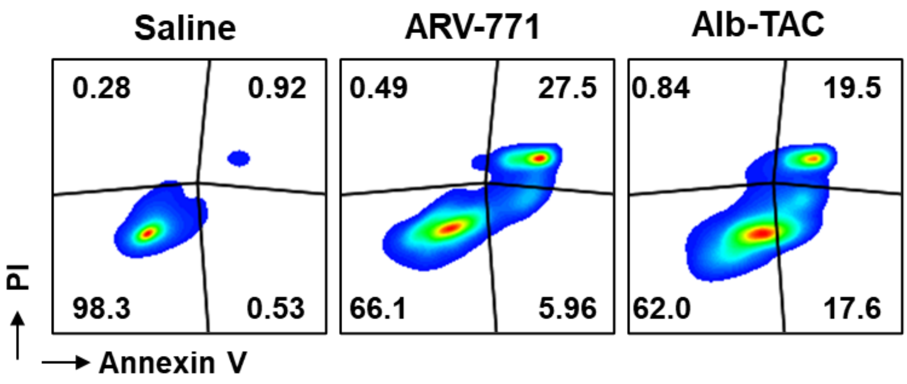

Figure S14. Flow cytometric analysis of cell death mode induced by Alb-TAC. Representative flow cytometry scatter plots of CT26 cells stained with Annexin V-FITC and PI after 48 h of treatment with 5 μM ARV-771 or 5 μM Alb-TAC. The quadrants represent viable (Annexin V−/PI−), early apoptotic (Annexin V+/PI−), and late apoptotic/necrotic (Annexin V+/PI+) cell populations. Data are presented as mean ± SD (n = 3).

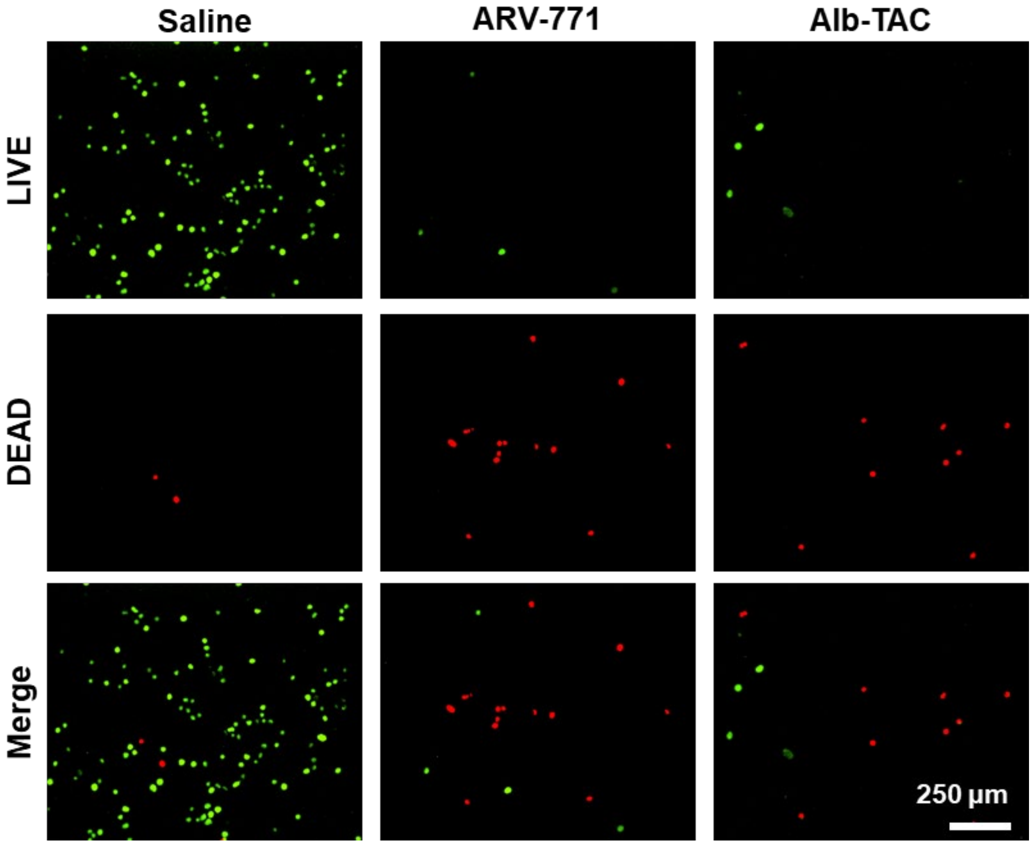

Figure S15. Results of live/dead viability assay of CT26 cells treated with Alb-TAC and ARV-771. Representative fluorescence microscopy images of CT26 cells treated with 5 µM ARV-771 or Alb-TAC for 48 h, followed by staining with calcein-AM (live, green) and 7-aminoactinomycin D (7-AAD; dead, red) (scale bar, 250 µm).

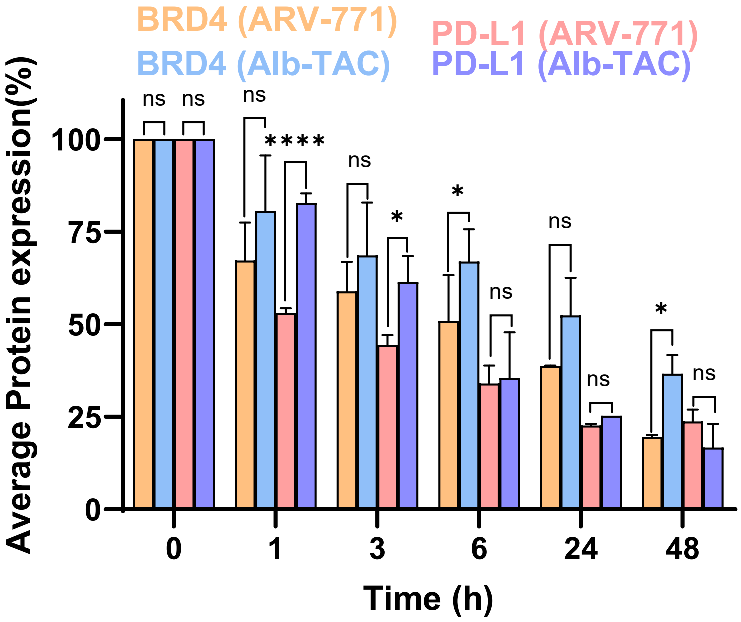

Figure S16. Quantitative analysis of BRD4 and PD-L1 band intensities from Figure 3C. Western blot analysis of CT26 cells treated with 10 μM ARV-771 or Alb-TAC for different incubation times (0, 1, 3, 6, 24, and 48 h) to assess time-dependent degradation kinetics of BRD4 and its downstream proteins (PD-L1, c-Myc, and Bcl-2). Data are presented as mean ± SD. Statistical significance was determined by two-way ANOVA with Tukey's post-hoc test (ns, not significant; $*p < 0.05$; $****p < 0.0001$).

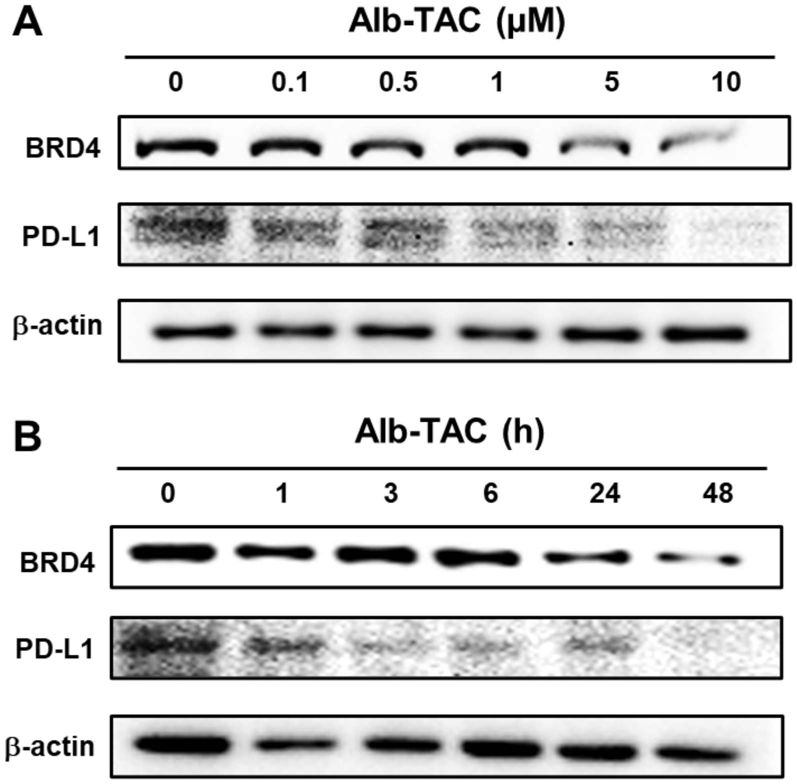

Figure S17. ***In vitro*** **BRD4 and PD-L1 degradation of Alb-TAC in 4T1 murine mammary carcinoma model.** (A) Western blot analysis of 4T1 cells treated with increasing concentrations (0, 0.1, 0.5, 1, 5, and 10 μM) of Alb-TAC for 24 h to evaluate BRD4 and PD-L1 degradation. (B) Western blot analysis of 4T1 cells treated with 10 μM Alb-TAC for different incubation times (0, 1, 3, 6, 24, and 48 h) to assess time-dependent degradation kinetics of BRD4 and PD-L1.

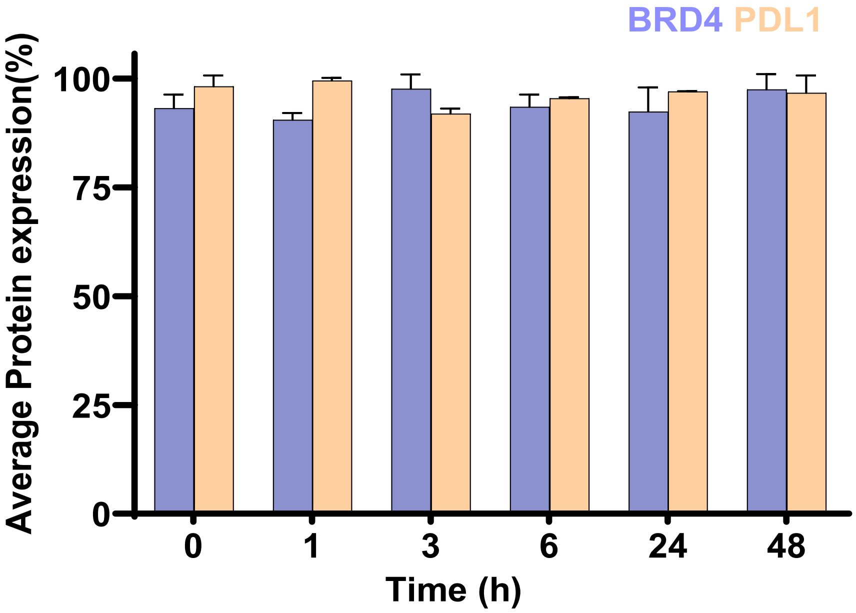

Figure S18. Quantitative analysis of BRD4 and PD-L1 band intensities from Figure 3D. Western blot analysis of CT26 cells co-treated with Alb-TAC (10 μM) and the VHL inhibitor VH298 to verify VHL-dependent BRD4 degradation over time.

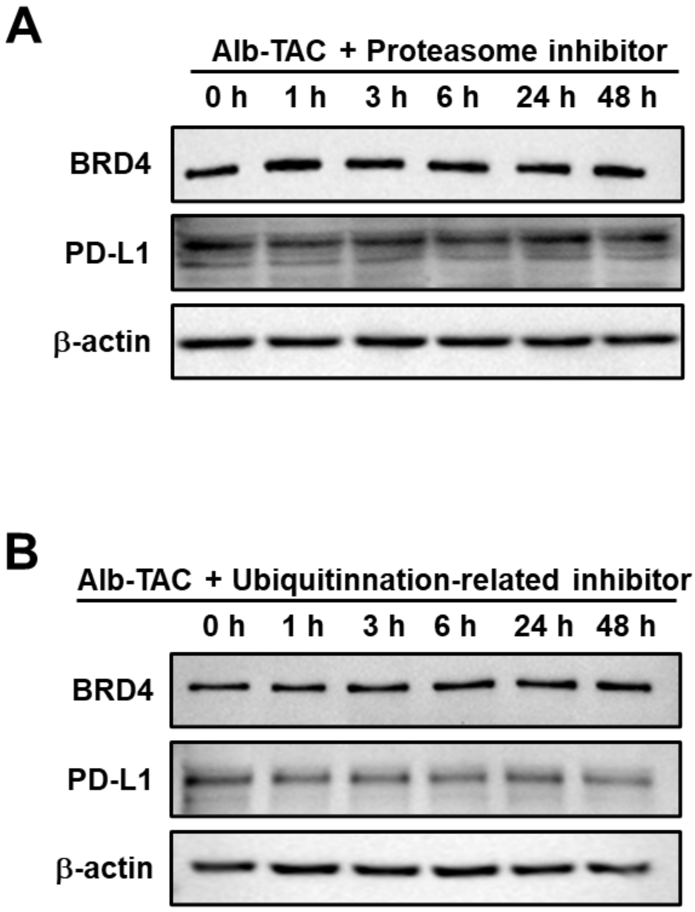

Figure S19. Confirmation of the ubiquitin-proteasome-dependent degradation pathway of Alb-TAC. Western blot analysis of BRD4 and PD-L1 levels in CT26 cells co-treated with Alb-TAC and (A) NAE inhibitor Pevonedistat (5 μM) or (B) proteasome inhibitor Bortezomib (10 nM) over 48 h.

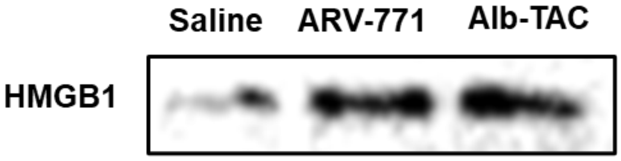

Figure S20. Western blot analysis of extracellular HMGB1 release following Alb-TAC treatment. Representative Western blot of HMGB1 levels in culture supernatants collected from CT26 cells treated with 10 μM ARV-771 or 10 μM Alb-TAC for 24 h.

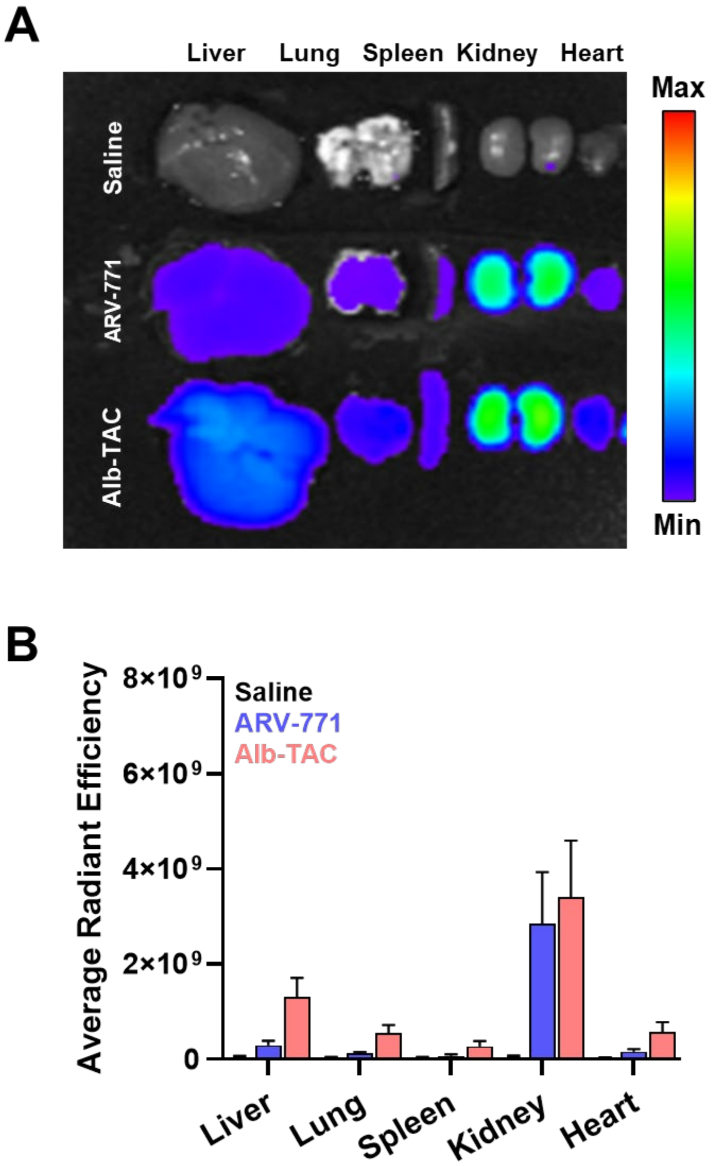

Figure S21. ***Ex vivo*** **biodistribution of Cy5.5-labeled ARV-771 and Alb-TAC in CT26 tumor-bearing mice.** (A) Representative *ex vivo* near-infrared fluorescence (NIRF) images of major organs (liver, lung, spleen, kidney, and heart) collected 24 h after intravenous injection of Cy5.5-labeled ARV-771 or Cy5.5-labeled Alb-TAC (3 mg/kg; n = 3 per group). (B) Quantitative analysis of fluorescence intensity of major organs.

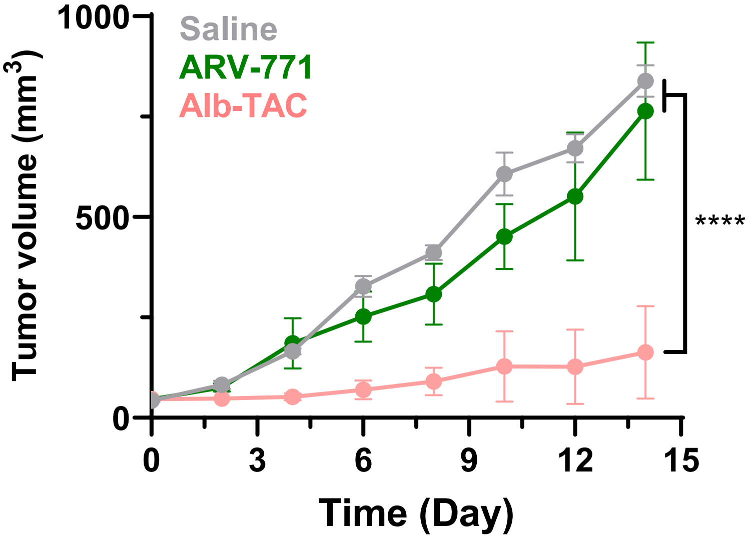

Figure S22. ***In vivo*** **anti-tumor efficacy of Alb-TAC in 4T1 murine mammary carcinoma model.** Tumor growth curves of 4T1 tumor-bearing mice treated intravenously with saline, ARV-771 (10 mg/kg), or Alb-TAC (10 mg/kg, equiv. to ARV-771) every three days for four doses (days 0, 3, 6, 9 and 12). Data are presented as mean ± SD. Statistical significance was determined by two-way ANOVA with Tukey's post-hoc test (****$p < 0.0001$).

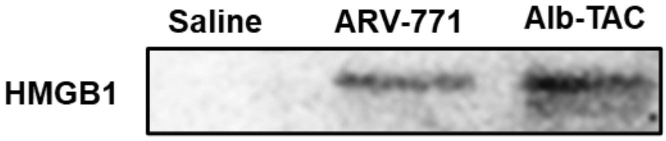

Figure S23. Western blot analysis of HMGB1 expression in tumor tissues following Alb-TAC treatment. Representative Western blot of HMGB1 levels in tumor lysates collected from CT26 tumor-bearing mice treated with saline (control), ARV-771 (10 mg/kg), or Alb-TAC (10 mg/kg) for 7 days.

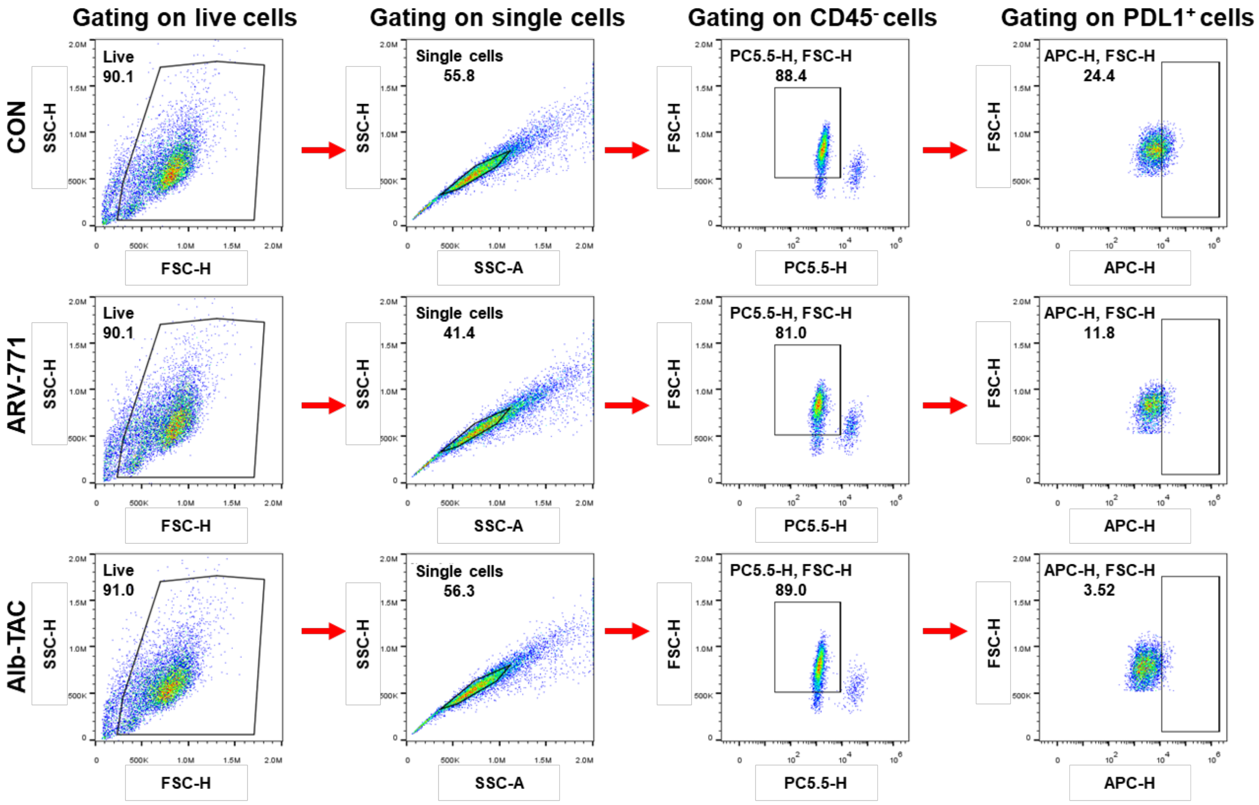

Figure S24. Gating strategy for PD-L1-expressing immune cells. Representative flow cytometry plots illustrating the gating sequence for PD-L1 expression on tumor cells.

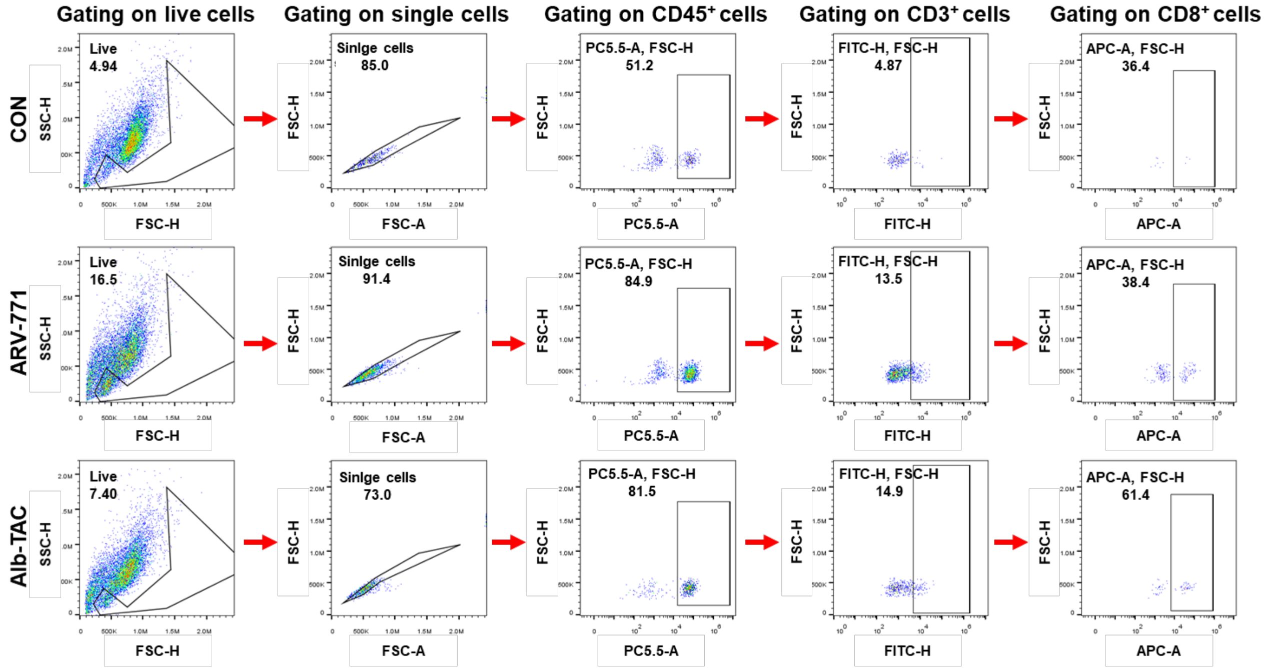

Figure S25. Gating strategy for CD8$^+$ T cells. Representative flow cytometry plots illustrating the gating sequence for CD8$^+$ expression on T cell subsets.

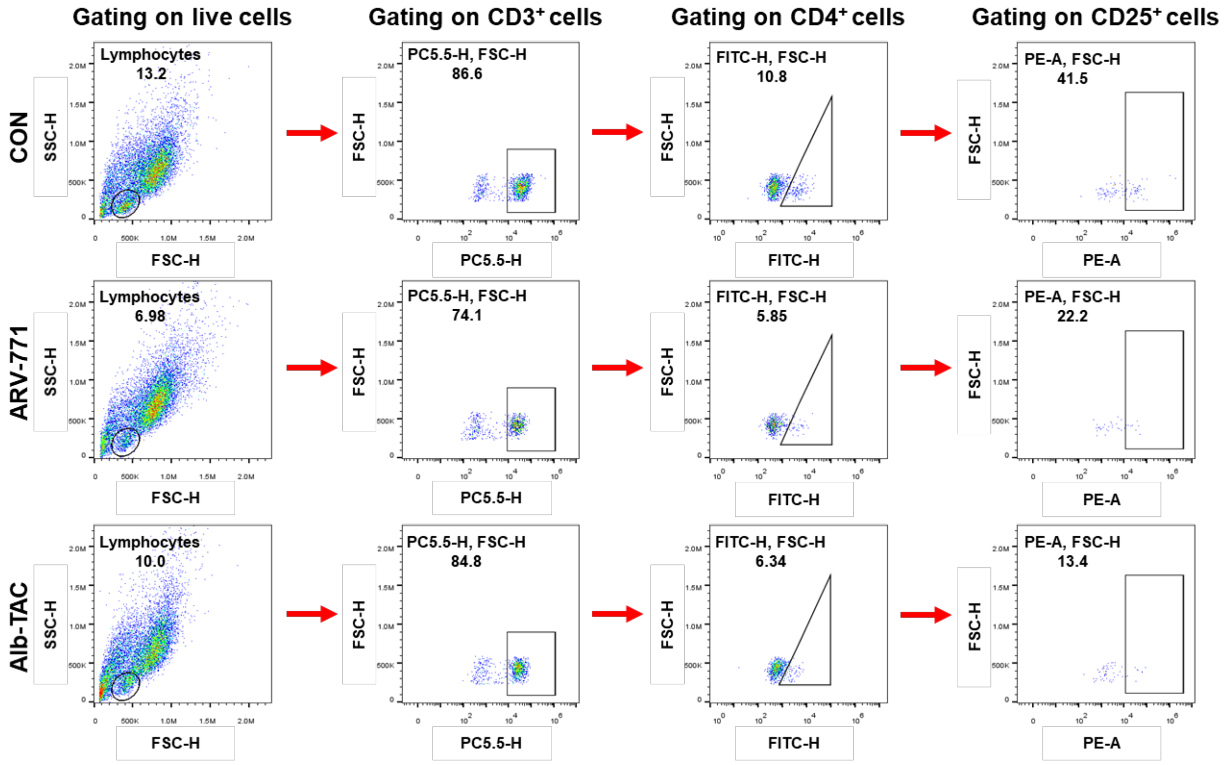

Figure S26. Gating strategy for regulatory T cells. Representative flow cytometry plots illustrating the gating sequence for $CD25^+$ expression on T cell subsets.

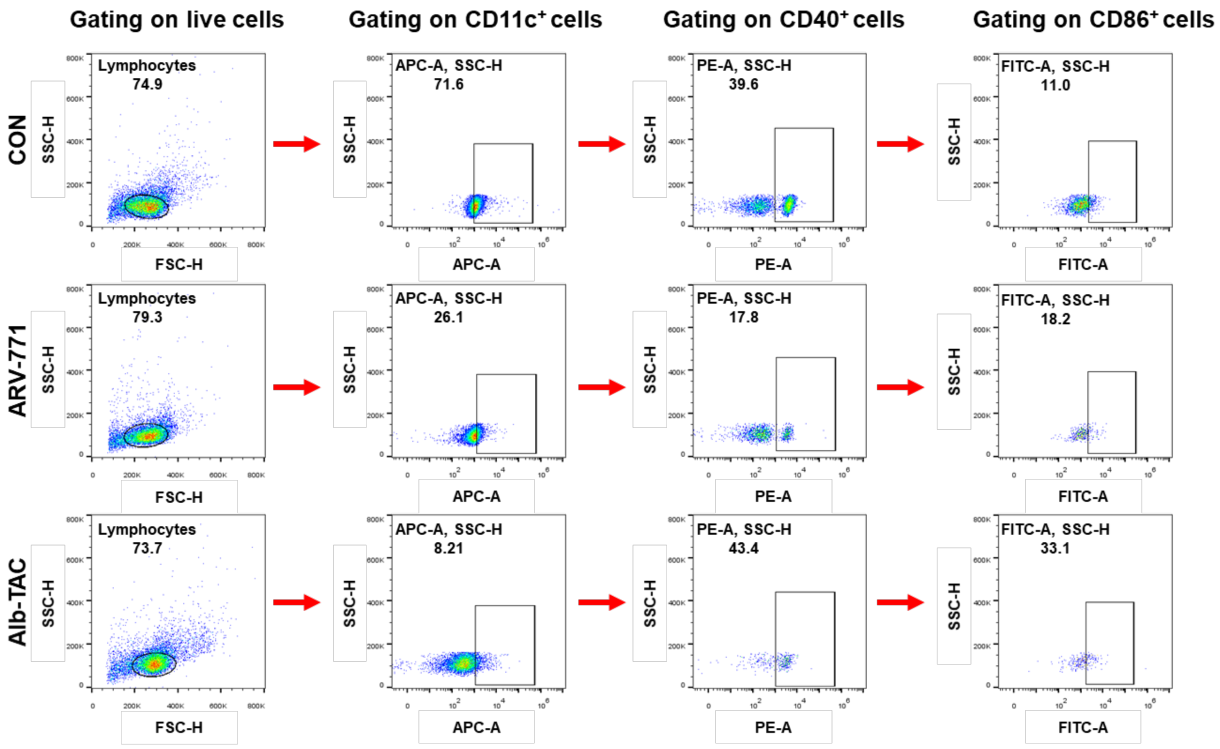

Figure S27. Gating strategy for mature dendritic cells (DCs). Representative flow cytometry plots illustrating the gating sequence for CD86^{+} expression on DC subsets.

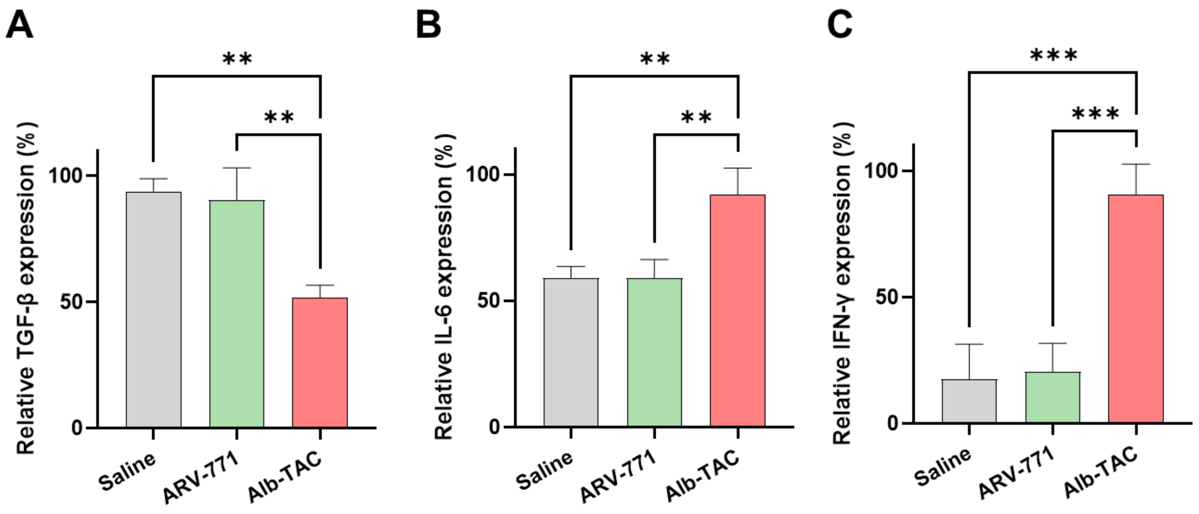

Figure S28. Functional cytokine profiling within the TME. Relative expression levels of (A) TGF-β, (B) IL-6, and (C) IFN-γ in the plasma of CT26 tumor-bearing mice. Data are presented as mean ± SD (n = 3). Statistical significance was determined by one-way ANOVA with Tukey's post-hoc test for (C, D) (**p < 0.01; ***p < 0.001).

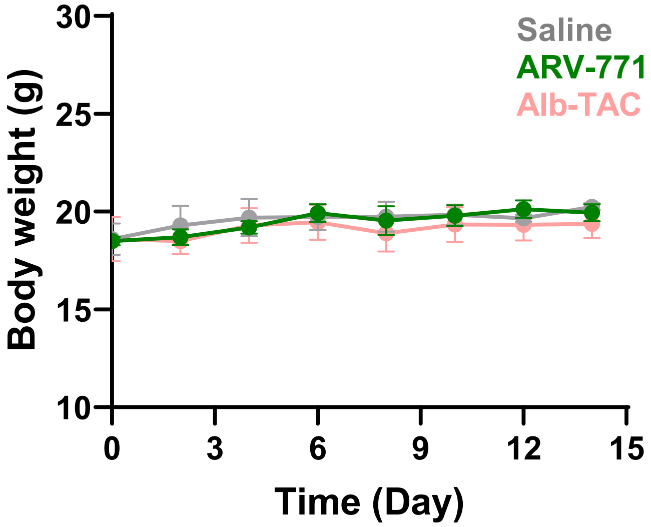

Figure S29. *In vivo* safety of Alb-TAC in 4T1 murine mammary carcinoma model. Body weight changes of 4T1 tumor-bearing mice treated intravenously with saline, ARV-771 (10 mg/kg), or Alb-TAC (10 mg/kg, equiv. to ARV-771) every three days for four doses (days 0, 3, 6, 9 and 12).

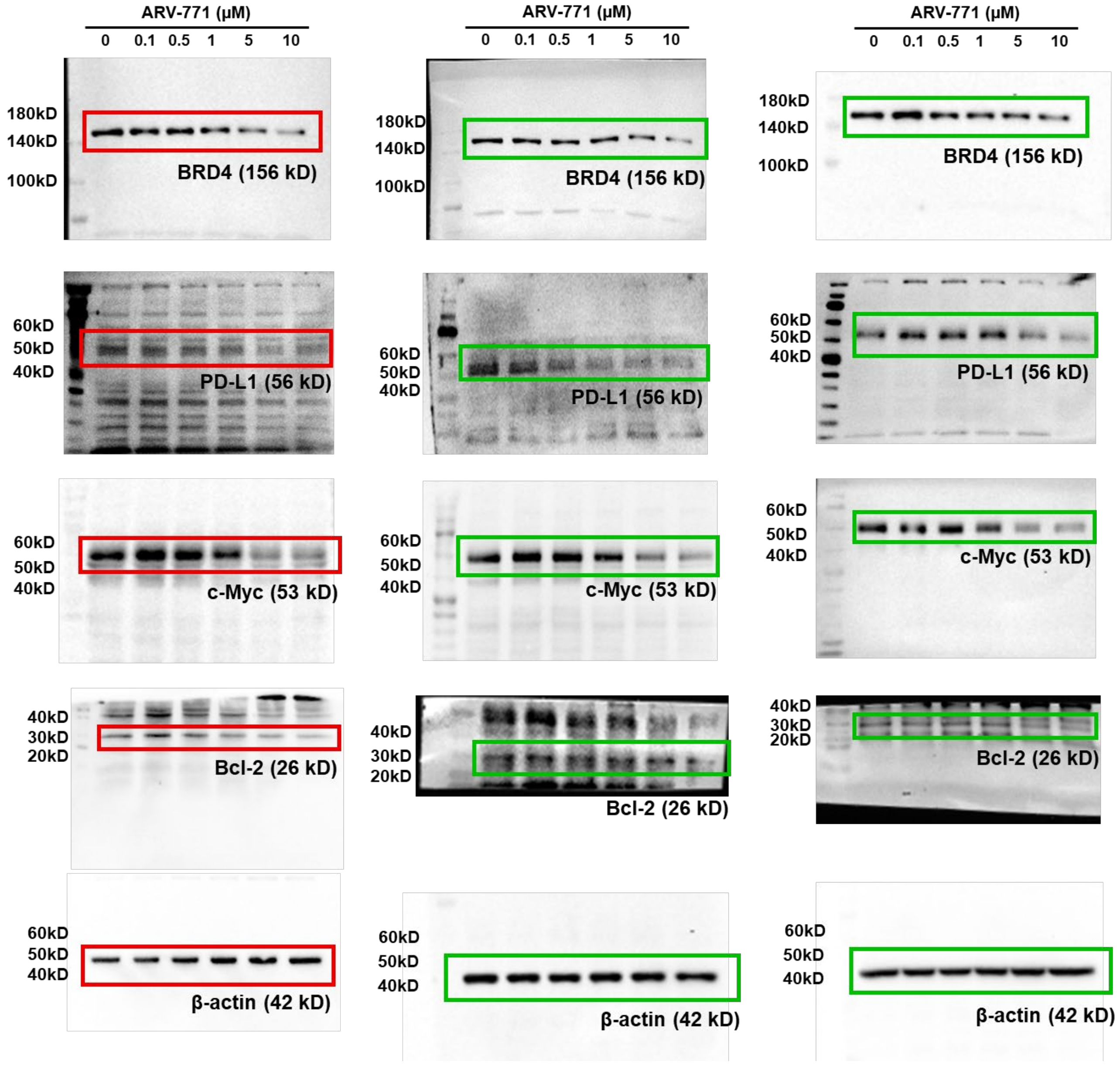

Figure S30. Uncropped western blot results of ARV-771-treated samples in Figure 3A. Western blot analysis of CT26 cells treated with increasing concentrations (0, 0.1, 0.5, 1, 5, and 10 μM) of ARV-771 for 24 h to evaluate BRD4, PD-L1, c-Myc, Bcl-2, and β-actin protein levels. Red boxes indicate the cropped regions presented in **Figure 3A**. Green boxes indicate the remaining two independent biological replicates. All data represent three independent biological replicates.

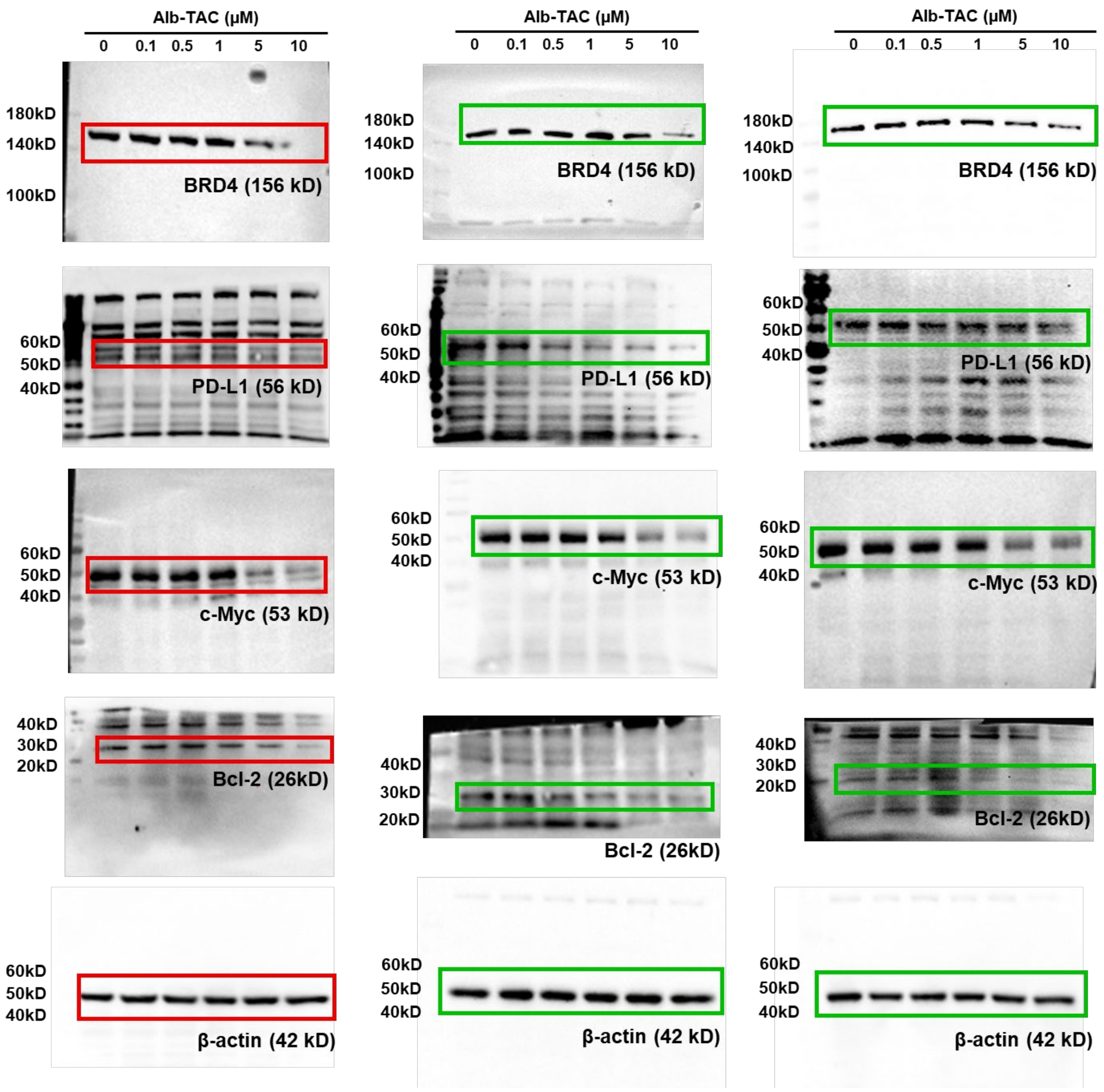

Figure S31. Uncropped western blot results of Alb-TAC-treated samples in Figure 3A. Western blot analysis of CT26 cells treated with increasing concentrations (0, 0.1, 0.5, 1, 5, and 10 µM) of Alb-TAC for 24 h to evaluate BRD4, PD-L1, c-Myc, Bcl-2, and β-actin protein levels. Red boxes indicate the cropped regions presented in **Figure 3A**. Green boxes indicate the remaining two independent biological replicates. All data represent three independent biological replicates.

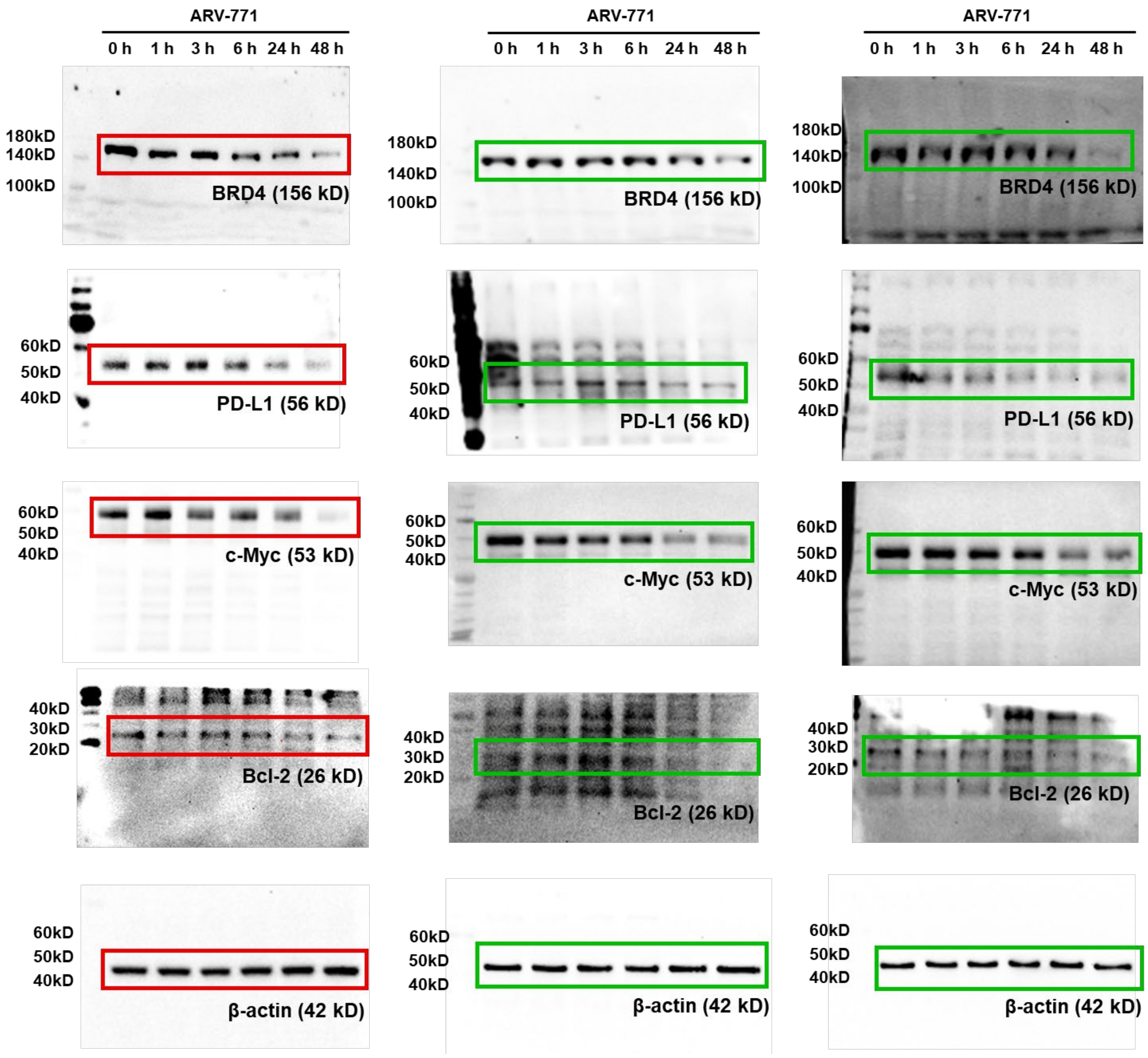

Figure S32. Uncropped western blot results of ARV-771-treated samples in Figure 3C. Western blot analysis of CT26 cells treated with 10 μM ARV-771 for different incubation times (0, 1, 3, 6, 24, and 48 h) to evaluate BRD4, PD-L1, c-Myc, Bcl-2, and β-actin protein levels. Red boxes indicate the cropped regions presented in **Figure 3C**. Green boxes indicate the remaining two independent biological replicates. All data represent three independent biological replicates.

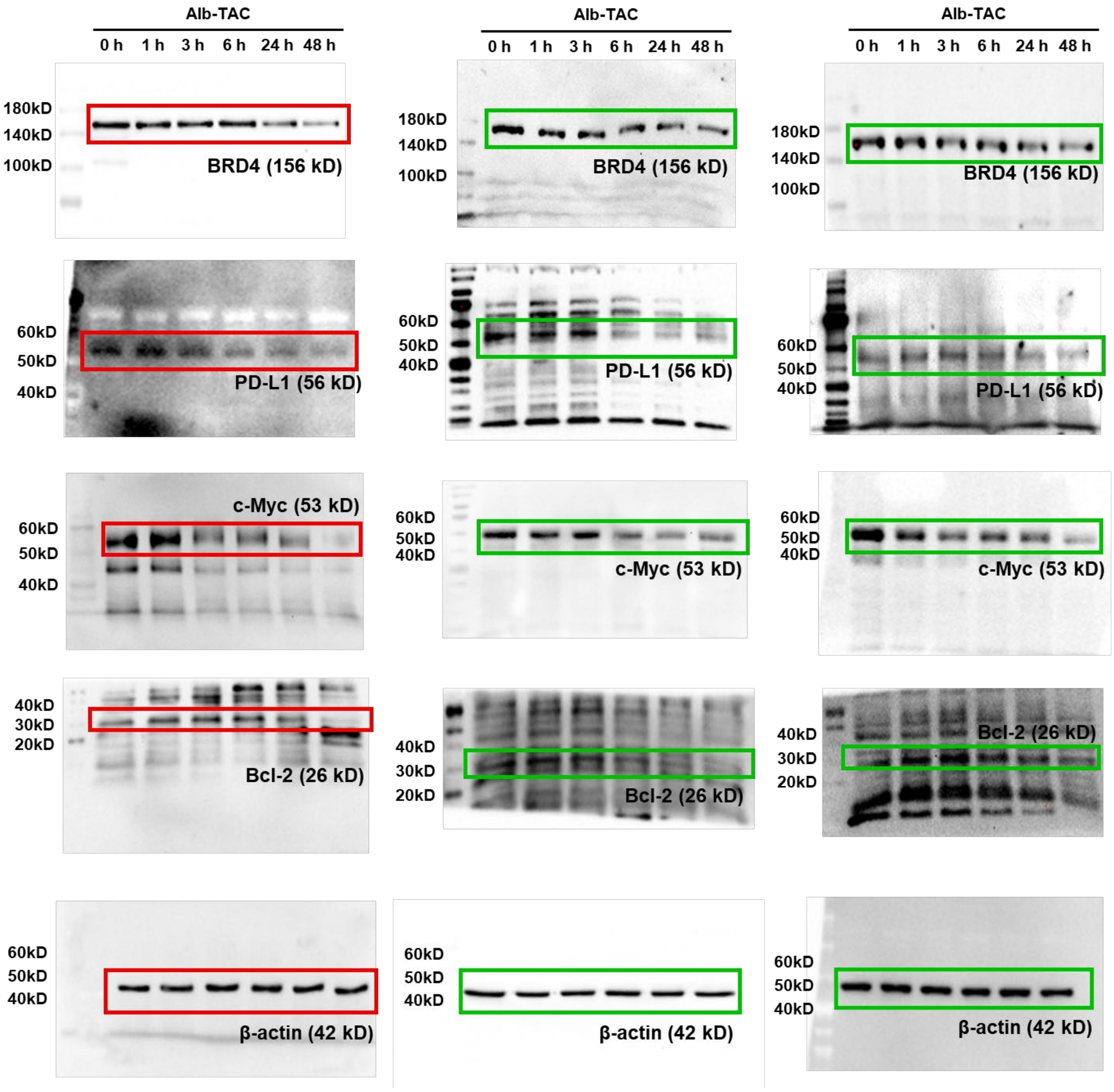

Figure S33. Uncropped western blot results of Alb-TAC-treated samples in Figure 3C. Western blot analysis of CT26 cells treated with 10 μM Alb-TAC for different incubation times (0, 1, 3, 6, 24, and 48 h) to evaluate BRD4, PD-L1, c-Myc, Bcl-2, and β-actin protein levels. Red boxes indicate the cropped regions presented in **Figure 3C**. Green boxes indicate the remaining two independent biological replicates. All data represent three independent biological replicates.

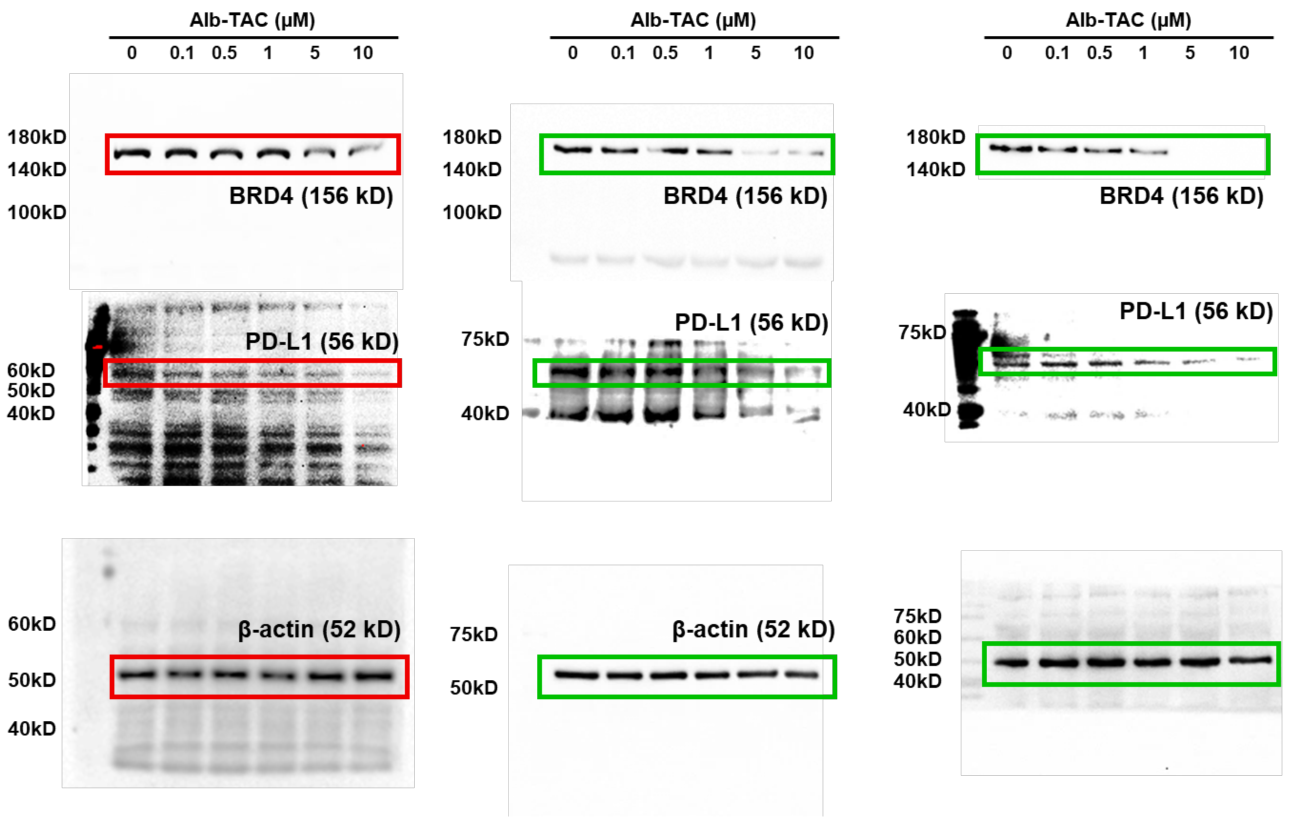

Figure S34. Uncropped western blot results of Alb-TAC-treated samples in Figure S17A. Western blot analysis of 4T1 cells treated with increasing concentrations (0, 0.1, 0.5, 1, 5, and 10 μM) of Alb-TAC for 24 h to evaluate BRD4, PD-L1, and β-actin protein levels. Red boxes indicate the cropped regions presented in **Figure S17A**. Green boxes indicate the remaining two independent biological replicates. All data represent three independent biological replicates.

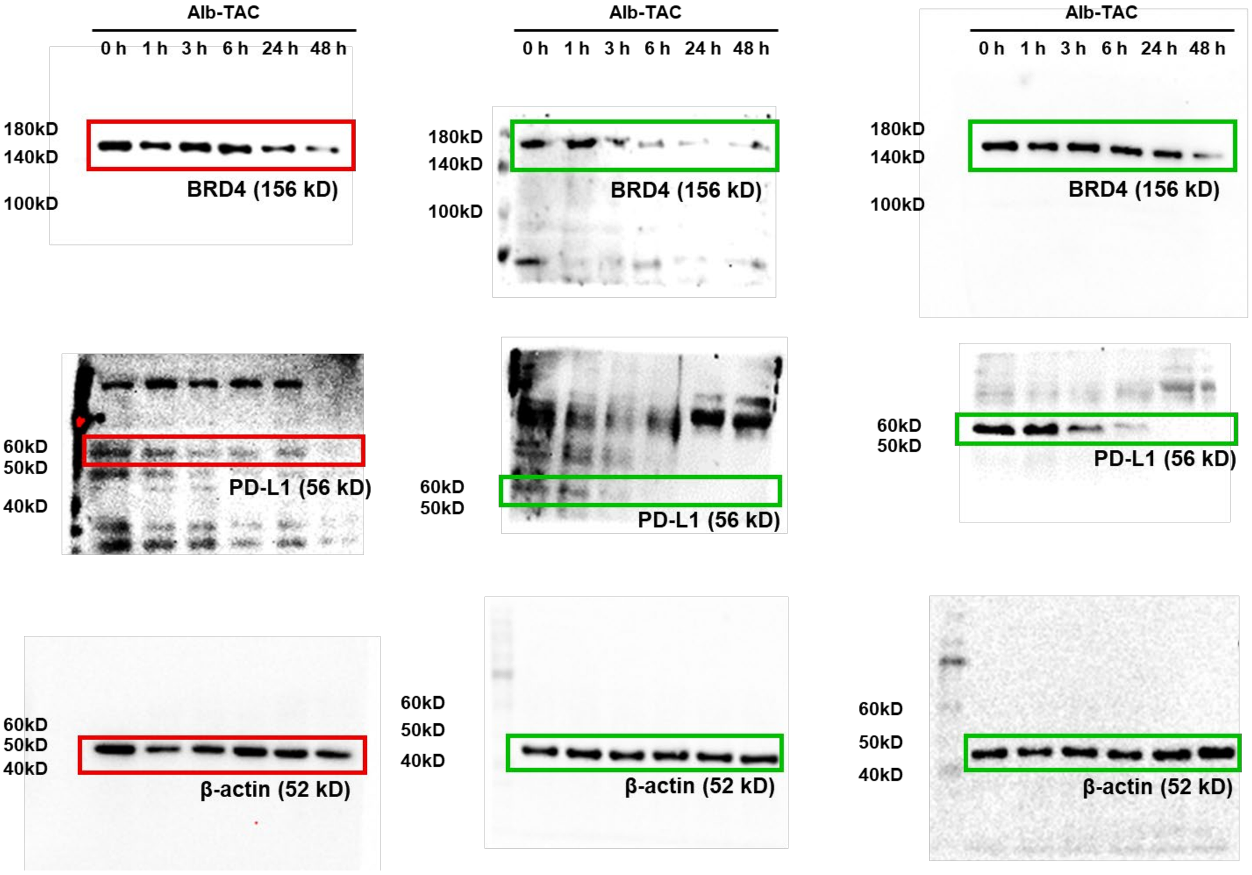

Figure S35. Uncropped western blot results of Alb-TAC-treated samples in Figure S17B. Western blot analysis of 4T1 cells treated with 10 μM Alb-TAC for different incubation times (0, 1, 3, 6, 24, and 48 h) to evaluate BRD4, PD-L1, and β-actin protein levels. Red boxes indicate the cropped regions presented in **Figure S17B**. Green boxes indicate the remaining two independent biological replicates. All data represent three independent biological replicates.

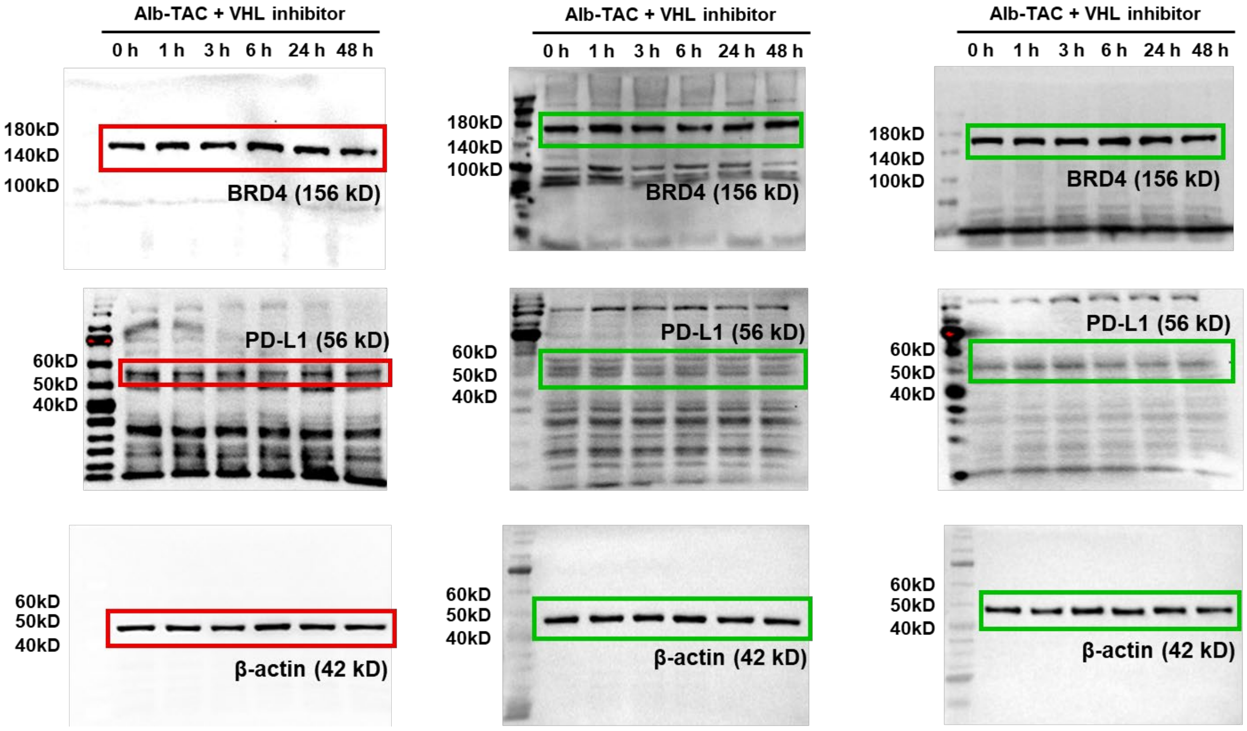

Figure S36. Uncropped western blot results of Figure 3D. Western blot analysis of CT26 cells treated with 10 µM Alb-TAC with 50 µM VHL inhibitor for different incubation times (0, 1, 3, 6, 24, and 48 h) to evaluate BRD4, PD-L1, and β-actin protein levels. Red boxes indicate the cropped regions presented in **Figure 3D**. Green boxes indicate the remaining two independent biological replicates. All data represent three independent biological replicates.

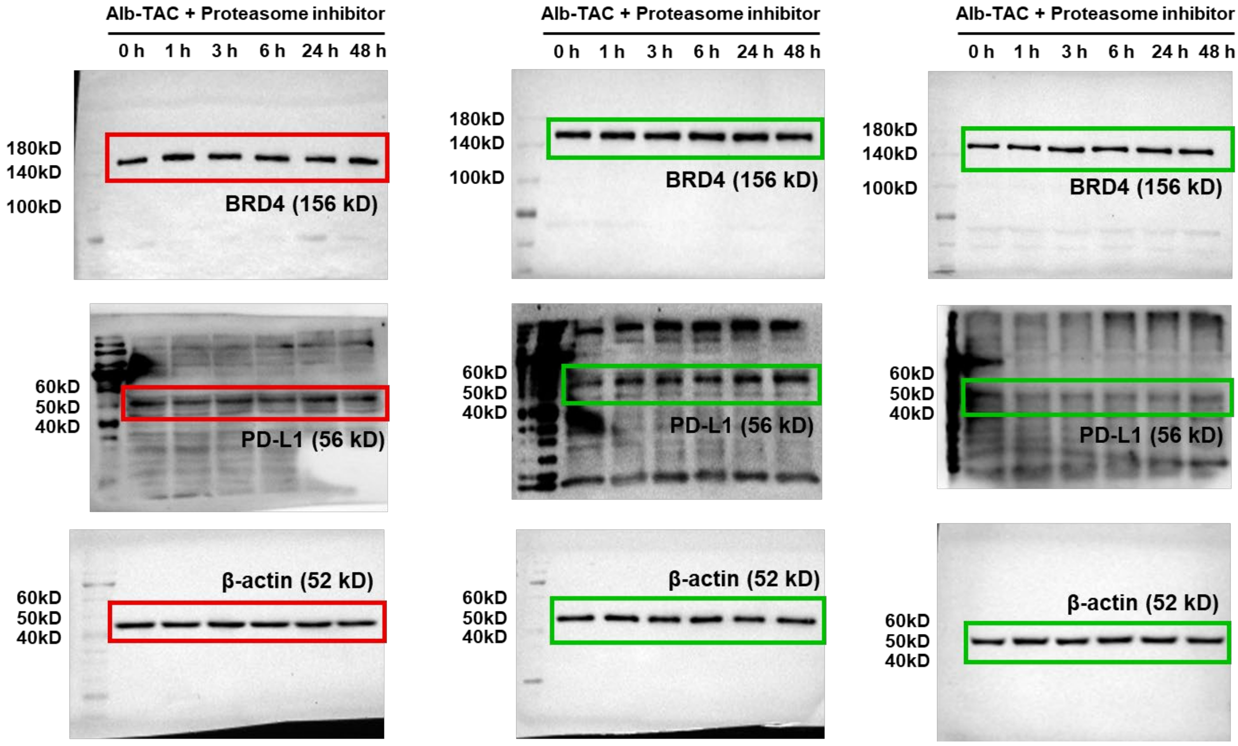

Figure S37. Uncropped western blot results of Figure S19A. Western blot analysis of CT26 cells treated with 10 μM Alb-TAC with 10 nM proteasome inhibitor for different incubation times (0, 1, 3, 6, 24, and 48 h) to evaluate BRD4, PD-L1, and β-actin protein levels. Red boxes indicate the cropped regions presented in **Figure S19A**. Green boxes indicate the remaining two independent biological replicates. All data represent three independent biological replicates.

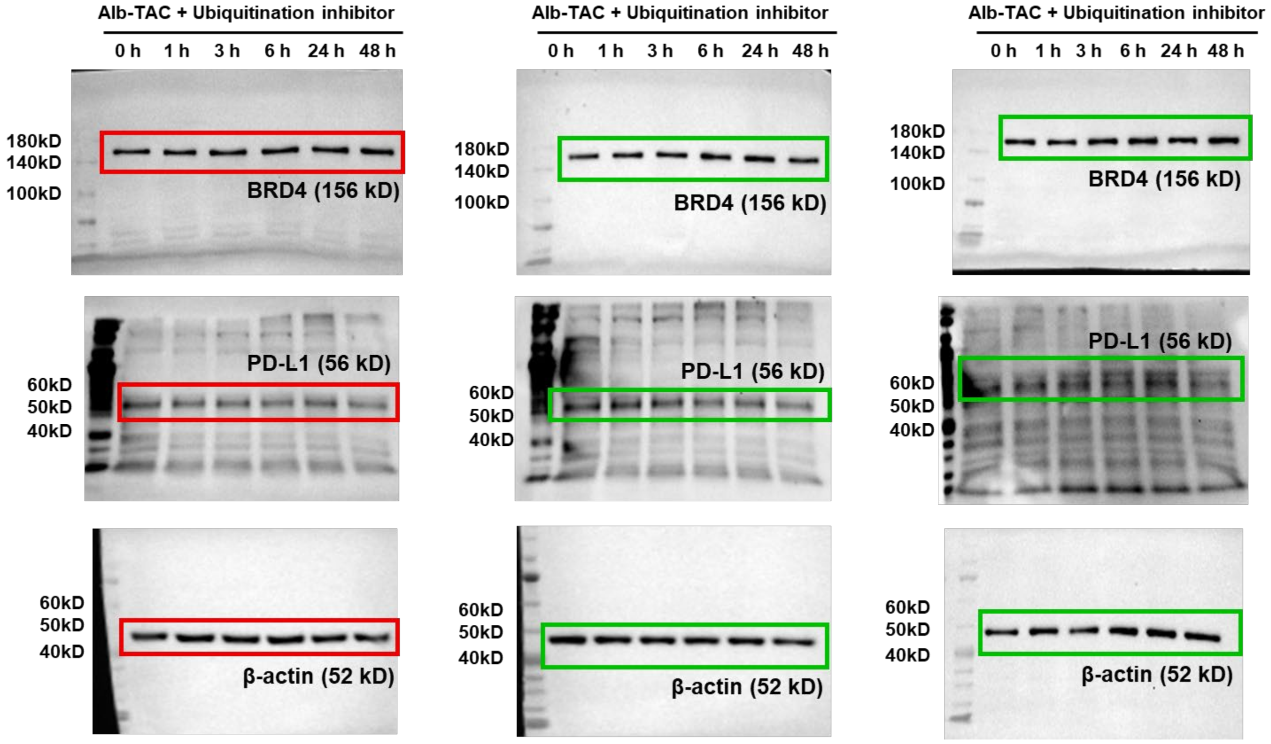

Figure S38. Uncropped western blot results of Figure S19B. Western blot analysis of CT26 cells treated with 10 μM Alb-TAC with 5 μM ubiquitination inhibitor for different incubation times (0, 1, 3, 6, 24, and 48 h) to evaluate BRD4, PD-L1, and β-actin protein levels. Red boxes indicate the cropped regions presented in **Figure S19B**. Green boxes indicate the remaining two independent biological replicates. All data represent three independent biological replicates.

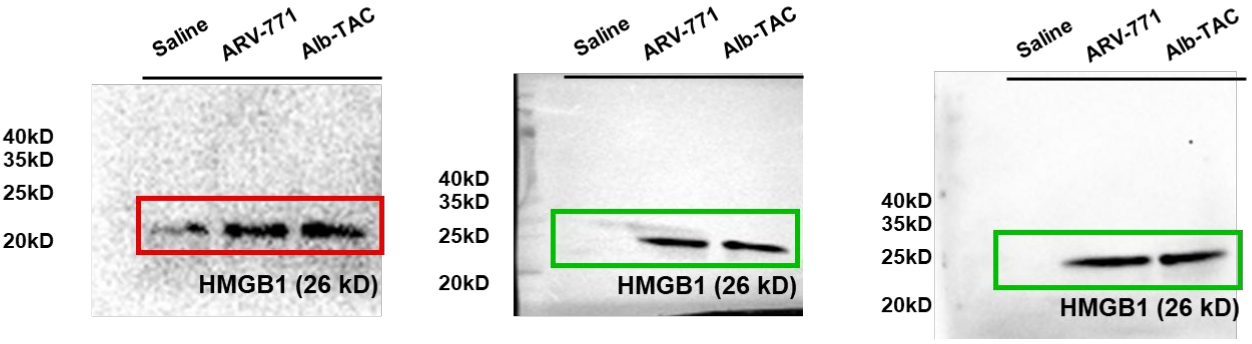

Figure S39. Uncropped western blot results of Figure S20. Western blot analysis of HMGB1 levels in culture supernatants collected from CT26 cells treated with saline, 10 µM ARV-771, or 10 µM Alb-TAC for 24 h. Red boxes indicate the cropped regions presented in **Figure S20**. Green boxes indicate the remaining two independent biological replicates. All data represent three independent biological replicates.

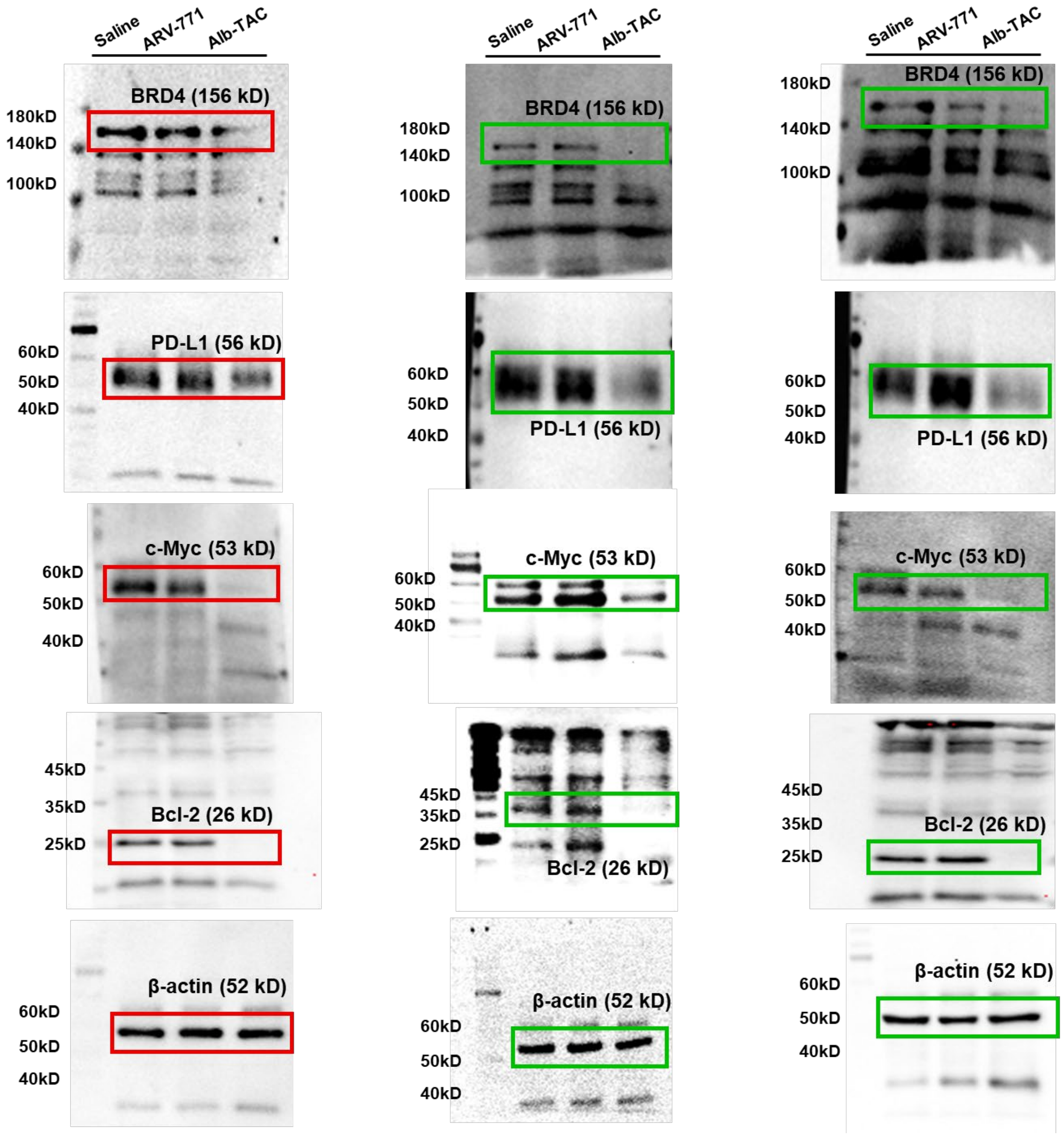

Figure S40. Uncropped western blot results of Figure 5D. Western blot analysis of tumor lysates from CT26 tumor-bearing mice in each group (saline, ARV-771 (10 mg/kg), and Alb-TAC (10 mg/kg)) showing expression of BRD4, PD-L1, c-Myc, Bcl-2, and β-actin protein levels. Red boxes indicate the cropped regions presented in **Figure 5D**. Green boxes indicate the remaining two independent biological replicates. All data represent three independent biological replicates.

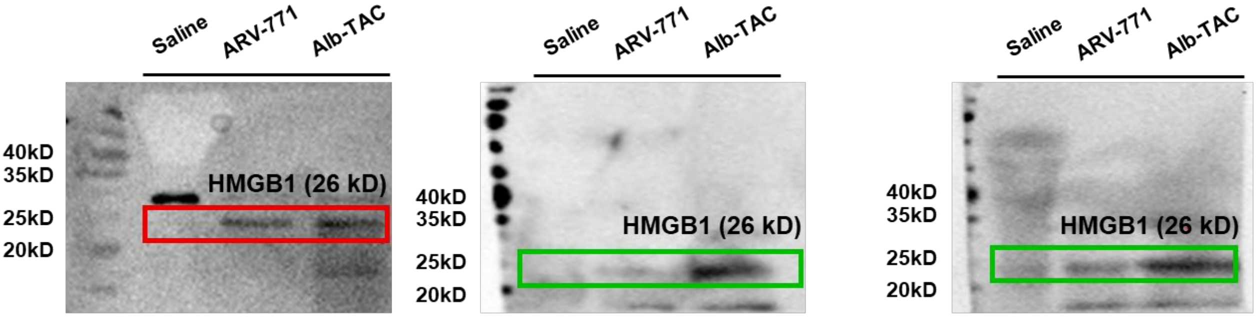

Figure S41. Uncropped western blot results of Figure S23. Western blot analysis of HMGB1 levels in tumor lysates from CT26 tumor-bearing mice in each group (saline, ARV-771 (10 mg/kg), and Alb-TAC (10 mg/kg)). Red boxes indicate the cropped regions presented in **Figure S23**. Green boxes indicate the remaining two independent biological replicates. All data represent three independent biological replicates.
